# Supplementary material for: Sprouty2 loss‐induced IL6 drives castration‐resistant prostate cancer through scavenger receptor B1
Source: EMBO Mol Med. 2018 Mar 14;10(4):e8347. doi: 10.15252/emmm.201708347 (PMC5887544; doi:10.15252/emmm.201708347)
Supplement: Supplementary file 1 — Appendix [file EMMM-10-e8347-s001.pdf]

APPENDIX

TITLE

Sprouty2 loss induced IL6 drives castration-resistant prostate cancer through scavenger receptor B1

AUTHORS

Rachana Patel*, Janis Fleming, Ernest Mui, Carolyn Loveridge, Peter Repiscak, Arnaud Blomme, Victoria Harle, Mark Salji, Imran Ahmad, Katy Teo, Freddie C. Hamdy, Ann Hedley, Niels van den Broek, Gillian Mackay, Joanne Edwards, Owen J. Sansom and Hing Y. Leung*

* Corresponding authors

TABLE OF CONTENT

1.	Appendix Information (detailed methods)
2.	Appendix Figures (S1, S2 & S3)
3.	Appendix Legends
4.	Appendix Tables S1, S2

Appendix Supplementary Information:

Study approval- All the animal experiments conducted for this study were carried out with ethical approval from University of Glasgow under the revised Animal (Scientific Procedures) Act 1986 and the EU Directive 2010/63/EU (PPL 30/3185). Animals were housed in individual ventilated cages in a barrier facility proactive in environmental enrichment. Tissue Microarrays (TMAs) were already available for analyses from a previous study. In brief, patients were retrospectively identified from Greater Glasgow and Clyde NHS Trust and retrieved from the pathology archives. Patients were diagnosed between 1993 and 2002 and were only included if samples were surplus to diagnostic need and sufficient material was available for TMA construction. Areas of low and high Gleason score were identified by pathologist to address tumour heterogeneity and 0.6 mm^2 cancer cores were taken from each area for TMA construction. TMAs were constructed in triplicate resulting in 6 cores being available for analysis for each patient. Ethical approval was gained from the West of Scotland Research Ethics Committed (05/S0704/94). An informed consent was obtained from all subjects and that the experiments conformed to the principles set out in the WMA Declaration of Helsinki and the Department of Health and Human Services Belmont Report.

Cell lines- All the human normal (RWPE-1) and prostate cancer (CWR22Res, CWR22RV1, LNCaP, LNCaP AI and VCaP) cell lines were mycoplasma negative, authenticated by LGC standards. CWR22Res and CWR22RV1 (22RV1) cells are cell lines derived from CWR-22 xenografts (Nagabhushan et al, 1996). CWR22Res cells (hormone responsive variant of CWR22 cells) were obtained from Case Western Reserve University, Cleveland, Ohio. RWPE-1 (ATCC® CRL-11609™), LNCaP (ATCC® CRL-1740™), VCaP (ATCC® CRL-

2876™) and CWR22RV1 (22RV1) (ATCC® CRL-2505™; the CRPC variant of CWR22) (Sramkoski et al, 1999) were obtained from ATCC. RWPE-1 cells were maintained in Keratinocyte Serum Free Medium (K-SFM) (Invitrogen GIBCO: 17005-042) with (bovine pituitary extract (BPE) and human recombinant epidermal growth factor (EGF). CWR22Res, LNCaP and VCaP prostate cancer cells were grown in RPMI with glutamine containing 10% FBS. The androgen independent LNCaP AI cell line and CWR22RV1 were used as a model to study the effects of chronic androgen deprivation treatment. LNCaP AI cells (generated from LNCaP cells through chronic steroid deprivation over a period of 9 months mimicking androgen deprivation therapy were donated by Professor Craig Robson, University of Newcastle) were grown in phenol red free RPMI with glutamine containing 10% charcoal stripped (steroid depleted) serum. CWR22RV1 cells were also maintained in phenol red free RPMI medium with glutamine containing 10% charcoal stripped (steroid depleted) serum. The STR cell line authentication of CWR22Res and CWR22RV1 was done using Qiagen Puregene Core Kit B for gDNA extraction with Promega Geneprint 10 kit for PCR and Genemapper v4 software for gene fragment analysis on 3130xl Genetic Analyser (Applied Biosystems) (Appendix table 1).

LNCaP-SPRY2 cells were generated by transfecting LNCaP cells with human SPRY2 expressing plasmid (pCDNA3.1) as described previously (Patel et al, 2013). Stable Sprouty2 (SPRY2) knockdown in CWR22Res cells was achieved using pTER$^+$ plasmid with hairpin targeting SPRY2 as previously described (Patel et al, 2013) with nucleofection (Lonza-kit V). CWR22Res cells stably expressing shSPRY2 (Clones- CL3 and Pool) and shNsi (non-targeting scrambled shRNA) were selected using zeocin (300 μg/ml; Invitrogen). Stable shHER2 knockdown clones were generated by transfecting shHER (SantaCruz Biotechnology- sc-94978-SH) and shscram (SantaCruz Biotechnology- sc-108060) in CWR22Res cells stably expressing shNsi or shSPRY2 (Pool). The HSD3B1 and SRB1 knock

out CWR22Res cells were generated using CRISPR-Cas9 technology from Santa Cruz Biotechnology: HSD3B1 CRISPR/Cas9 KO Plasmid (sc-400825), HSD3B1 HDR Plasmid (sc-400825-HDR), SR-B1 CRISPR/Cas9 KO Plasmid (sc-400990) and SR-B1 HDR Plasmid (sc-400990-HDR). The PTEN siRNA (6251) and control siRNA (6568) was obtained from Cell Signaling. siRNA transfection was carried out with nucleofection (Lonza-kit V).

The pre-adipocyte 3T3-L1 cells (gift from Dr Ian Salt, Institute of Cardiovascular and Medical Sciences, University of Glasgow) were differentiated into adipocytes using adipocyte differentiation protocol. Briefly, to differentiate the 3T3-L1 fibroblast cells into adipocytes, the cells were grown to confluency and fed at confluency in DMEM with 10% newborn calf serum (NCS). At 48 hr post-confluence, cell medium was aspirated and replaced with differentiation medium consisting of DMEM with 10% fetal calf serum (FCS) containing dexamethasone, methyl isobutylxanthine (IBMX), troglitazone and insulin. After a further three days, this medium was aspirated and replaced with DMEM containing 10% FCS containing troglitazone and insulin only. The cells were incubated in this medium for a further three days, and then the medium was aspirated and replaced with DMEM containing 10% FCS (no other additions). At 8-12 days, post-induction of differentiation, cells were used for experimentation.

For growth rate assays, equal number of cells was seeded and counted using the CASY®cell counter (Innovatis) at two and four days post treatment. The treatments were initiated 12 hr after seeding the cells. The growth rate was calculated relative to the cell count at time 0 (T0, Day 0) i.e., cell number obtained at the time of treatment. The IC50 of ITX5061 was calculated using WST-1 reagent (Roche) to assay cell viability after 48 hr of treatment. The ITX5061 dose response was analysed relative to respective DMSO. IC50 was calculated

using log (inhibitor) vs. response -- Variable slope (four parameters) with Bottom constraint=0.0 with GaphPad Prism software.

Immunohistochemistry and Immunoblotting – Immunohistochemical (IHC) and immunoblotting was performed as previously described (Patel et al, 2013). The additional antibodies used for IHC were SPRY2 (Abcam, ab60719- 1:1000 dilution for immunoblotting), AR (Santa Cruz Biotechnology, sc-816- - 1:1000 dilution for immunoblotting and 1:100 dilution for IHC), IL6 (Abcam, ab6672- 1:60 dilution for IHC), HSD3B1 (Abcam, ab55268 (for human samples- 1:1000 dilution); ab65156 (for mouse samples- 1:1000 dilution), Perilipin (Cell Signaling Technology, 9349- 1:1000 dilution), Phospho-(Ser/Thr) PKA substrate (Cell Signaling Technology, 9621- 1:1000 dilution), cleaved caspase 3 (Cell Signaling Technology, 9661- 1:50 dilution for IHC), HSC70 (Santa Cruz Biotechnology, sc-7298- 1:1000 dilution), p-p38 (Cell Signaling Technology, 4511- 1:1000 dilution for immunoblotting and 1:50 dilution for IHC), HER2 (Cell Signaling Technology, 2165- 1:50 dilution for IHC), EGFR (Cell Signaling Technology, 4405- 1:1000 dilution for immunoblotting), SRB1 (Novus Biologicals, NB400-104- 1:50 dilution for IHC and 1:1000 dilution for immunoblotting), HSC70 (Santa Cruz Biotechnology, sc- sc-7298- 1:1000 dilution for immunoblotting) and GAPDH (Sigma, G9295- 1:10,000 dilution for immunoblotting).

Human tissue microarray- The matched tissue microarray (TMA) comprised of formalin-fixed, paraffin-embedded (FFPE) prostate cancer sections from 35 hormone naïve prostate cancer (HNPC) at diagnosis and castration-resistant prostate cancer (CRPC) matched prostate cancer patients (Tan et al, 2011). Hormone naïve samples are primary prostate tumour tissues

before the patients received ADT and the matched CRPC samples are primary prostate tumour tissue from the same individuals after biochemical evidence (rise in serum PSA) of CRPC. In this TMA, the patients have received only ADT treatment. Ethical approval was acquired from the Multicentre Research Ethics Committee for Scotland (MREC/01/0/36) and Local Research and Ethics Committees. These patients were selected based on their initial response to hormone treatment (sub capsular bilateral orchidectomy or LHRH agonist +/- antiandrogens) and their subsequent relapse (2 rises in PSA >10%). CRPC samples were gained by TURP as a result of relieving bladder outflow obstruction. Clinico-pathological data available included age (69.5, IQR 64.5-74.8), Gleason sum score at diagnosis (median 8, range 6-9), Gleason sum score at relapse (median 9, range 8-9), serum prostate specific antigen (PSA) levels at diagnosis (34.5 ng/mL, IQR 9.9-130.5), and PSA at relapse (20 ng/mL, IQR 4.5-39). All patients relapsed, with a median time to disease recurrence being 2.6 years (IQR 1.7-4.8 years) and median overall survival being 5.5 years (IQR 3.4-7.2 years). The CRPC samples from this TMA were used for correlation analyses of PTEN and SPRY2.

For assaying the effects of SPRY2 and SRB1 levels on response to treatment and survival, SRB1 IHC was performed in prostate cancer TMA which included diagnostic biopsies from 90 prostate cancer patients treated with ADT, radiotherapy and chemotherapy. To assess the effects of SPRY2 on overall survival, patients treated with hormone therapy and evidence of biochemical relapse were selected (n=19). Patients were segregated into SPRY2 high (n=12) with IHC scores above the average median IHC score and SPRY2 low (n=7) with IHC scores below the average median IHC score. To assess the effects of SRB1 levels on treatment resistance and survival, 37 patients treated with ADT (n=37) were selected for further analyses. Out of these, 15 patients had prior surgeries and/or radiotherapy. To assess the time to biochemical relapse, patients with only ADT treatment (n=22) and no prior surgeries and/or radiotherapy were selected for analyses. To investigate the effects of SR-B1 levels on

the overall survival post-diagnosis, patients with biochemical evidence of CRPC (n=27) were selected for analyses. The CRPC samples from this TMA were used for IL6 correlations with HER2 and SPRY2.

The stained slides were scored by two independent observers blinded to clinical parameters, using a weighted histoscore method, also known as the H-score, at 40X magnification. For each stained section, the staining intensity and percentage of cells stained were scored. For semi-quantitative classification of staining intensity, the histoscore was calculated as (1 × % cells staining weakly positive) + (2 × % cells staining moderately positive) + (3 × % cells staining strongly positive). The TMA cores with insufficient tissue to attribute IHC score were not included in further analyses.

Clinical datasets for gene alterations and survival analyses- Relapse-free survival based on SPRY2 expression was queried using cBioPortal platform (Cerami et al, 2012; Gao et al, 2013) for cancer genomics using TCGA, provisional prostate cancer dataset. The data was extracted and Kaplan-Meier survival curves were plotted in GraphPad Prism.

Microarray- mRNA was harvested from three biological replicates of CWR22Res prostate cancer cells with Nsi (non-silencing vector control) expression or stable SPRY2 knockdown (Pool). Samples for microarray were generated using Illumina sequencing reagents and HumanHT-12 v4 Expression BeadChip Kit. Microarray hybridisation was performed at BHF Glasgow Cardiovascular Research Centre (Taurino et al, 2010) and chips were scanned a BeadArray Reader (Illumina). Bioinformatics analyses were carried out at CRUK Beatson Institute, Glasgow, UK. The Illumina microarray data is deposited in Geo Gene Expression Omnibu: GEO Submission (GSE108456).

Gene Set Enrichment Analyses (GSEA)- GSEA was carried out using microarray data obtained from Nsi (non- silencing vector control) and stable SPRY2 deficient (Pool) CWR22Res cells. The GSEA v2.2.2 tool (Mootha et al, 2003; Subramanian et al, 2005) was used for analysis and GSEA was performed as follows: - All genes assessed by microarray were ranked using the difference of means scaled by the standard deviation (option Signal2Noise in the GSEA program) and weighted scoring scheme was used. A null distribution was created using the gene set permutation type and 1000 permutations. The ranked datasets were tested against gene sets termed 'C2: curated gene sets' and 'C5: GO gene sets' from Molecular Signatures Database (MSigDB) v5.1 as well as specifically against cytokine, lipase, lipid, lipoprotein and steroid related genesets extracted from MSigDB v5.1.

Studies using clinical serum samples- Serum samples from 172 patients with HNPC and 129 patients with CRPC were obtained from ProMPT study (ethics committee approval: UK MREC number 01/4/61). All clinical information including serum PSA levels were obtained from patient medical records. IL6 was assayed in these serum samples in duplicates using R&D (S6050) human IL6 ELISA kits as per the kit protocol. Serum free fatty acid levels were assayed in duplicates using Abcam (ab65341) FFA colorimetric detection kit per protocol provided. Serum levels of PSA, FFA and IL6 levels were carried out in the same serum samples from individual patients. Data analyses were carried out using Graph Pad Prism software. The HN patient cohort (n=172) included patients with localised prostate cancer. Most of these HN patients (n=123) were untreated when the serum samples were collected for the analyses presented here. A subset of HN patients were treated with radical prostatectomy (n=41) and EBRT (external beam radiation therapy) (n=8). All patients in

CRPC cohort (n=129) showed clinical or biochemical disease progression post ADT treatment. Majority of CRPC patients (n=121) were treated with ADT alone while few were treated with EBRT (n=3) or radical prostatectomy (n=5) prior to ADT treatment. The retrospective patient records were used to acquire the data on PSA levels. For IL6 and PSA correlations, PSA values were obtained from the same serum samples (matched for sample collection date) from individual patients (n=42). Same criteria were applied for IL6 and free fatty acid (FFA) correlations. For this, FFA was measured in a subset of 18 ProMPT serum samples (9 each of HNCP and CRPC cases) previously used for IL6 analyses. For survival analyses, CRPC patients with no recorded evidence of metastases (M0) were selected.

Genetically engineered mouse models (GEMM)- *Nkx 3.1-Cre* (Hsieh et al, 2010) mice were crossed to those harbouring *Spry2*$^{fl/+}$(Shim et al, 2005) and *Pten*$^{fl/+}$(Lesche et al, 2002) , and mice were genotyped by PCR by Transnetyx™. After approximately 50 weeks, the *Nkx 3.1-Cre Pten*$^{fl/+}$ *Spry2*$^{fl/+}$ (NPS) developed palpable prostate tumours (Gao et al, 2012). The mice with palpable prostate tumours were randomised into two groups- mock (sham surgical incision) and androgen deprivation therapy (ADT) which was achieved by orchiectomy. A subset of mice (n=6) was euthanised at 1 month after (Mock or ADT) treatment, and prostates and lymph nodes were placed in formalin for 24 hr fixation before paraffin embedding. Rest the Mock (n=7) and ADT (n=6) treated mice were aged to clinical end point. The clinical end point, based on the Animal licence (PPL 30/3185), comprised of either loss of more than 20% of the pre-treatment body weight, sluggish behaviour, loss of mobility or excessive haematuria.

For anti-IL6 treatment, ADT treated mice were injected intraperitoneally twice weekly with a 500 µg rat anti-murine IL-6 mAb (R&D- MAB406) or control antibody in 200 µl PBS for

four weeks after which the mice were euthanised, with the prostates collected and formalin fixed. For ITX5061 treatment, ADT treated mice were gavaged with 25 mg/kg/day ITX5061 in 20% hydroxypropyl-Beta-cyclodextrin in 20 mM citric acid for a month after which the mice were euthanised, with the prostates collected and formalin fixed.

Orthograft prostate cancer model- Prostate orthograft model using CWR22Res cells was established. For prostate orthograft animal experiments, CD1-nude male mice (6 to 8 weeks old) were obtained from Charles River Research Models & Services (UK). A midline lower abdominal incision was made on the mice anesthetized by isoflurane inhalation. Using a 1-cc syringe with a 27-gauge needle, $14x10^6$ CWR22Res cells in 50 µl of serum free phenol red free glutamine containing RPMI were injected in one of the anterior prostate lobes to form a localized bleb within the injected prostatic lobe. The mice with injections forming an intact prostatic bleb were retained for the study. The abdominal cavity was closed with surgical sutures and the outer skin was closed using surgical staples. Mice were treated with Carprofen (dose defined by the assigned veterinarian) as an analgesic agent prior to and 24 hr post-surgery. After 30 days when a palpable tumour was felt with 100% tumour incidence, the mice were randomised into two groups: Mock (sham surgical incision) and ADT (orchiectomy). For characterisation of the ADT treatment on CWR22Res cells, ultrasound based analysis was carried out at 30 days post intra-prostatic injections and at 60 days (i.e., 30 days post Mock or ADT treatment). The ultrasound *in vivo* imaging was carried out using Vevo 3100 Imaging System and prostate orthograft volume analyses was carried out using FUJIFILM VisualSonics, Inc software.

To study the effects of SPRY2 deficiency on treatment response, a pilot experiment with 5 mice per treatment was performed. Here, approximately 14 million cells (Nsi control or

SPRY2 deficient clones –CL3 and Pool) were injected in one of the anterior prostate lobes of CD-1 nude mice. Ten mice per clone were used for this experiment. The orthografts were palpable around 30 days (4 weeks) post intra-prostatic injections with 100% incidence. These mice were then randomised to receive Mock or ADT treatment (n=5 per treatment). All mock-treated animals achieved maximum permitted tumour burden around 73 days post-implantation. ADT treated mice with SPRY2 deficient orthografts showed adverse clinical signs such as weight loss around 60 days post implantation. Based on this we used a refined 60 days timed protocol to carry out further detailed investigations. Since the tumour incidence was 100% without considerable variation for Mock treated mice, we used n=5 per group in all our 60 days timed experiments.

The VCaP HN orthografts were generated in a similar manner by injecting $10x10^6$ cells in 50 µl of serum free phenol red free glutamine containing RPMI in one of the anterior prostate lobes of CD-1 nude mice. For VCaP CRPC orthografts, following orthotopic implantation of VCaP cells, the mice were castrated at the same time of intra-prostatic injections. The orthografts were collected at clinical endpoints.

Experimental treatments include Tocilizumab (100 µg in PBS given as I.P. injection 3 times a week for 3 weeks), Simvastatin (80 mg/kg/day in 30% PEG400 + 0.5% Tween80 + 5% Propylene glycol in water, gavaged daily for 1 month) and ITX5061 (25 mg/kg/day in 20% hydroxypropyl-Beta-cyclodextrin made in 20mM citric acid, gavaged once daily for 1 month). Individual treatments were initiated 3 days post-ADT. The mice were euthanised 60 days after drug or vehicle treatments; the prostates, epididymal adipose tissue, blood and other organs of interest including liver were collected for further analyses. The prostate orthografts were weighted and divided into two halves. One half was placed in formalin for 48 hr fixation before paraffin embedding. The rest of the orthograft was snapped frozen on

dry ice. The snap frozen prostate orthografts were pulverised for protein, RNA and steroid extraction and homogenised using micro-homogenizer for cholesterol (free and esterified) extraction. Adipose tissue collected was weighted and snap frozen for protein extraction (approximately 30 mg of adipose tissue was pulverised and used for protein extraction). Livers were excised and one of the lobes (largest) was snap frozen and rest of the tissue was formalin fixed for 48 hr before paraffin embedding. The frozen liver was further used for cryo-sectioning followed by Oil-O staining and pulverised for RNA extraction. The blood collected was allowed to stand (clot) for 15 min and then spun down at 4°C at 3000 r.p.m. for 15 min. The serum was collected and stored for further analyses of testosterone, alanine transaminase activity (ALT), triglycerides, cholesterol, IL6 and free fatty acids.

Treatment response in prostate cancer orthografts – The prostate orthografts were weighted and their dimensions (length & height) were measured using Vernier calliper. The tumour volume was calculated based as volume cm^3 = (length X height X width)/2.

To assess treatment response on established orthografts, we considered two aspects: (i) Treatment induced cell death within tumour and (ii) Treatment induced decrease in tumour growth. Assessment of a number of orthografts and treatments, both macroscopically and microscopically, revealed treatment induced tumour necrosis as a consistent factor in addition to tumour weight. After comparing different types of analyses to assess treatment response on established orthografts, relative tumour necrosis was found to be a consistent measure of response to treatment. Using Leica image analyser software the total tumour area and central necrotic area was calculated. This was used to calculate the % necrosis. Since the size and growth of the tumour may also result in central tumour necrotic core, the % necrosis was

normalised with the weight of the tumour. Thus, the data was represented as % necrosis/tumour weight.

Visceral Metastases – The visceral metastases burden was assessed based on both macroscopic and histological evidence of metastasis. The metastatic sites were broadly classified as proximal and distal metastases based on the localisation of metastatic foci. The proximal sites included lumbar lymph nodes (draining lymph nodes for prostate) and epididymal adipose tissue (present at a close proximity to prostate tumours). The distal sites included area that were not in immediate proximity to prostate tumours such as diaphragm and thoracic area which included lungs and thoracic lymph nodes. The metastatic foci were confirmed based on histopathological (H&E) and evidence of AR by immunohistochemistry analyses. For contingency analyses, each animal was analyses and scored for presence of proximal or distal metastases. For cumulative visceral metastases, both proximal and distal scores were assayed together i.e. 2 sites per mouse. Therefore, for experimental groups with 5 mice the contingency analyses is done based on 10 metastatic sites (2 sites x 5 mice= 10 sites).

Steroid Measurements- Serum and intra-tumoural testosterone levels were measured using Testosterone EIA kit (Cayman Chemicals, 582701). For intra-tumoural testosterone measurement, approximately 30 mg of tumour was pulverized and the fine powder was suspended in 100 µl of PBS. The steroids were extracted from tissue homogenate or serum using five times the volume of diethyl ether twice. After thorough mixing and vortexing the upper layer was transferred to clean glass tube and allowed to dry at 30°C under gentle

stream of N_2. These tubes were left overnight for further drying so that the residue was completely free of any organic solvent. The remainder residue was dissolved in 500 µl of EIA buffer provided in the kit. The testosterone levels were measures by as per the protocol provided in the kit.

For LC-MS based steroid detections, the steroid extraction was carried out as described before (Weng et al, 2010). Briefly, prostate orthografts were weighted and homogenised using Polytron model in sterile PBS at 100 mg/ml. 20 µl of homogenate was saved for protein estimation by BCA method. To the rest of the homogenate for each biological sample, a mix of internal standard was added. The internal standard was a mix of Androstenedione-$^{13}C_3$, Dehydroepiandrosterone-D5, Testosterone $^{13}C_3$ and 5 alpha- Dihydrotestosterone-D3 steroids (at 100 ng each). The fortified homogenates were extracted with 8 ml of 60% hexane/40% ethyl acetate mix by shaking the tubes for 1 hour at room temperature. After centrifugation at 1000 g for 10 min, the organic phase was transferred to another tube and evaporated to dryness under a steady stream of N_2. The residues were dissolved in 20 µl of methanol followed by addition of 150 µl of steroid depleted charcoal stripped serum. All samples were precipitated with 300 µl of a solution of 4:1 methanol: 89 g/l $ZnSO_4$. After thoroughly mixing to ensure complete precipitation, the samples were centrifuged at 3220 g for 10 min. To the 300 µl of resultant supernatant, 900 µl of 4% H_3PO_4 was added and the samples were thoroughly mixed. The resultant samples were then directly applied to the solid phase extraction plate, Oasis PRiME HLB µElution plate (Waters), in 2 aliquots. All used columns in the plate were washed twice with 200 µl of 25% methanol. The samples were then eluted twice with 25 µl of 90:10 acetonitrile:methanol and diluted with 25 µl of double distilled water. 5 µl was injected into the mass spectrometer. A Q-Exactive Plus Orbitrap mass spectrometer was used together with an UltiMate 3000 HPLC system (Thermo Scientific, Waltham, MA, USA), with a HSS T3 UPLC column (50 x 2.1 mm, 1.8 µm, Waters). The

initial mobile phase was 70% aqueous (0.1% formic acid in water) and 30% organic (0.1% formic acid in acetonitrile) and steroids were separated over a 2.5 minute gradient, increasing the organic mobile phase content to 95%. The flow rate of 0.6 ml/min and a column temperature of 40°C were used throughout, and the total analysis time was 4.5 minutes. The steroids of interest and their labelled internal standards were detected using a targeted SIM (Selected Ion Monitoring) method, where the accurate mass of each steroid ion across its known retention time range were specifically measured. Electrospray (ESI) ionization in positive polarity was used with a mass resolution of 17,500. Data were acquired with Thermo Xcalibur software and peak areas for the steroids were determined using Thermo TraceFinder software. Overall, androstenedione and testosterone were detected consistently in all the samples. Using the same set up we could detect Dihydrotestosterone (DHT) in the standard mix but failed to detect it consistently in the orthograft samples. Our set up was unable to detect Dehydroepiandrosterone (DHEA) with confidence even in standard mix. The relative peak ratio was obtained by normalising the biological sample peak area to the peak area obtained from the corresponding fortified internal standard.

Lipid staining- For detecting lipid accumulation, Oil Red-O (Sigma) and BODIPY (Life Technologies) staining was used. The frozen sections of tissues were fixed with 4% paraformaldehyde (PFA) in PBS for 30 min at room temperature. For Oil Red-O staining, the stock solution of 1% dye in isopropanol was diluted with D.W. in 3:2 (dye: water) ratio and filtered. The sections were washed three times with PBS after 4% PFA fixation and stained with Oil-Red O for 5-10 min. The sections were then washed with water and counter stained with haematoxylin. For BODIPY fluorescence staining, the sections or glass bottom plates, after 4% PFA fixation, were washed with PBS followed by 10 min room temperature

incubation with 1.5 mg/ml glycine in PBS to quench the PFA. The sections were then stained with BODIPY (10 µg/ml in PBS) for 2 hr at room temperature. The sections were then washed with PBS and counterstained with DAPI. Confocal imaging was carried out using Nikon confocal microscope using 488 nm as the excitation wavelength and 505 nm as the emission wavelength.

Lipoprotein uptake assay- Lipoprotein uptake was carried up by treating the cells for 5 hr in respective culture medium containing 10 µg/ml of Dil-HDL (high density lipoprotein) or Dil-LDL (low density lipoprotein). Both Dil-LDL and Dil-HDL were obtained from Biomedical Technologies Inc. HDL and LDL uptake assays were also carried out using HDL Uptake Assay Kit (Fluorometric) (Abcam, ab204717) and LDL Uptake Assay Kit (Fluorometric) (Abcam, ab204716) kits, respectively. Briefly, the cells were seeded at sub-confluent density on glass bottom 96 well white plates. Next day, the cells were either treated with medium containing 10% FBS or 10% CSS (steroid depleted charcoal stripped serum mimicking ADT conditions). After two hours, the cells were washed with wash buffer provided in the respective kits and treated with HDL or LDL containing respective media with and without drugs (e.g. ITX5061). The cells were incubated for another 4 hours and washed with wash buffer provided in the kit. The fluorescence was measured using Safire machine as indicated in the kit. The standard curve was generated simultaneously and the amount of lipoprotein uptake was analysed from the standard curve using Graph Pad Prism software. The protein content in each well was measured using BCA protein estimation kit and amount of lipoprotein taken up by cells in each well was normalised to amount of total protein in respective wells.

Cholesterol efflux assay- The effects of ITX5061 on cholesterol efflux were measures in CWR22Res stable clones (as indicated) using Cholesterol Efflux Assay Kit (Cell-based) (Abcam, ab196985). Briefly, the cells were loaded with labelled cholesterol as per the protocol provided in the kit. The human serum was used as cholesterol acceptors. To assay the effects of drugs on cholesterol efflux, the cells were treated with ITX5061 after cholesterol loading along with serum for active efflux.

Real Time PCR- The RNA was extracted from ~70 mgs of pulverised orthografts using RNeasy mini kit (Qiagen). For cell lines, RNA was extracted from sub-confluent plates using RNeasy mini kit (Qiagen). 10 ug of RNA was used for cDNA synthesis using High capacity cDNA reverse transcription kit (Applied Biosystems). Real time PCR was carried out using TaqMan® Gene Expression Master Mix (Life Technologies) in 7500 Fast Real-Time PCR System (Applied Biosystems). The primers were designed using Universal Probe Library Assay Design Center (Roche) and the respective probes were used from the universal probe library. For all RT-PCR reactions, CACS3 was used as the house-keeping gene and the relative quantities for each assay were obtained by normalising to one biological control sample (as indicated in the figures). All results are presented as relative quantities (R.Q.) for hSPRY2 (Fwd- TTTGCACATCGCAGAAAGAA & Rev-TCAGGTCTTGGAAGTGTGGTC; hIL6 (Fwd-GATGAGTACAAAAGTCCTGATCCA & Rev-CTGCAGCCACTGG TTCTGT); hHSD3B1 (Fwd-TCTTCGGTGTCACTCACAGAG & Rev-GGCACACT AGCTTGGACACA); hHER2 (Fwd- GGGAAACCTGGAACTCACCT & Rev-CCCTGCACCTCCTGGATA); hPSA (Fwd-GTGCTTGTGGCCTCTCGT & Rev-CAGCAAGATCACGCTTTTGT); hSCARB1/SRB1 (Fwd-CCTGAGGACACCGTGAGC & Rev-GGTGTGCAACAGGCACAT); hLDLR (Fwd-CTACAAGTGGGTCTGCGATG &

Rev-TTTGCAGGTGACAGACAAGC); hCYP17A1 (Fwd-TCACCGTCAGTAAGCTATTTGC & Rev- GGGCCAGGATCTCACCTATAC); hHMGCR (Fwd- GTTCGGTGGCCTCTAGTGAG & Rev-TGCATTCGAAAAAGTCTTGACA); mHmgcr (Fwd- TGCGTAAGCGCAGTTCCT& Rev- TTGTAGCCTCACAGTCCTTGG); hAR full length (Fwd-CATGTGGAAGCTGCAAGGTCT & Rev- TCTGTTTCCCTTCAGCGGC) and hAR-V7 (Fwd- AACAGAAGTACCTGTGCGCC & Rev- TCAGGGTCTGGTCATTTTGA). The AR full length and AR-V7 variant (Jones et al, 2015) were assayed using BIO-RAD SYBR® Green method.

Tissue Cholesterol Quantification- Free and esterified cholesterol quantification was carried out using Cholesterol/Cholesteryl Ester quantitation assay kit (Abcam ab65359). For the prostate orthografts, pre-weighed orthografts were thawed and homogenised using micro-homogenizer. The cholesterol was extracted in 200 µl (per 10 mg of tissue) of chloroform: isopropanol: NP-40 (7:11:0.1) by mixing thoroughly and vortexing. The organic phase was collected in a separate tube after centrifugation for 10 min at 15,000x g. The samples were air dried at 50°C to remove chloroform. The samples were further vacuum dried for 30 min to remove trace organic solvent. The residue obtained was dissolved by sonication and vortexing with 200 µl of assay buffer provided in the kit. The quantitation assay was carried out with and without cholesterol esterase to assay free and esterified cholesterol levels.

Serum Analyses- Murine serum IL6 was measured using Interleukin-6 Mouse ELISA Kit (Abcam, ab100712). The triglycerides from murine sera were measured using Triglyceride Quantification Kit (Abcam, ab65336). Serum free fatty acids were measured using Free Fatty Acid Quantification Kit (Abcam, ab65341). The serum cholesterol was measured using HDL

and LDL/VLDL Cholesterol Assay Kit (Abcam, ab65390). Cholesterol from both fractions (HDL and LDL/VLDL) was added and presented as total serum cholesterol. Liver function was measured in the form of serum alanine transaminase activity (ALT) using Alanine Transaminase Activity Assay Kit (Colorimetric) (Abcam, ab105134).

References:

Cerami E, Gao J, Dogrusoz U, Gross BE, Sumer SO, Aksoy BA, Jacobsen A, Byrne CJ, Heuer ML, Larsson E, Antipin Y, Reva B, Goldberg AP, Sander C, Schultz N (2012) The cBio cancer genomics portal: an open platform for exploring multidimensional cancer genomics data. Cancer discovery 2**:** 401-404

Gao J, Aksoy BA, Dogrusoz U, Dresdner G, Gross B, Sumer SO, Sun Y, Jacobsen A, Sinha R, Larsson E, Cerami E, Sander C, Schultz N (2013) Integrative analysis of complex cancer genomics and clinical profiles using the cBioPortal. Science signaling 6**:** pl1

Gao M, Patel R, Ahmad I, Fleming J, Edwards J, McCracken S, Sahadevan K, Seywright M, Norman J, Sansom O, Leung HY (2012) SPRY2 loss enhances ErbB trafficking and PI3K/AKT signalling to drive human and mouse prostate carcinogenesis. EMBO molecular medicine 4**:** 776-790

Hsieh AC, Costa M, Zollo O, Davis C, Feldman ME, Testa JR, Meyuhas O, Shokat KM, Ruggero D (2010) Genetic dissection of the oncogenic mTOR pathway reveals druggable addiction to translational control via 4EBP-eIF4E. Cancer cell 17**:** 249-261

Jones D, Wade M, Nakjang S, Chaytor L, Grey J, Robson CN, Gaughan L (2015) FOXA1 regulates androgen receptor variant activity in models of castrate-resistant prostate cancer. Oncotarget 6**:** 29782-29794

Lesche R, Groszer M, Gao J, Wang Y, Messing A, Sun H, Liu X, Wu H (2002) Cre/loxP-mediated inactivation of the murine Pten tumor suppressor gene. Genesis 32**:** 148-149

Mootha VK, Lindgren CM, Eriksson KF, Subramanian A, Sihag S, Lehar J, Puigserver P, Carlsson E, Ridderstrale M, Laurila E, Houstis N, Daly MJ, Patterson N, Mesirov JP, Golub TR, Tamayo P, Spiegelman B, Lander ES, Hirschhorn JN, Altshuler D et al. (2003) PGC-1alpha-responsive genes involved in oxidative phosphorylation are coordinately downregulated in human diabetes. Nature genetics 34**:** 267-273

Nagabhushan M, Miller CM, Pretlow TP, Giaconia JM, Edgehouse NL, Schwartz S, Kung HJ, de Vere White RW, Gumerlock PH, Resnick MI, Amini SB, Pretlow TG (1996) CWR22: the first human prostate cancer xenograft with strongly androgen-dependent and relapsed strains both in vivo and in soft agar. Cancer research 56**:** 3042-3046

Patel R, Gao M, Ahmad I, Fleming J, Singh LB, Rai TS, McKie AB, Seywright M, Barnetson RJ, Edwards J, Sansom OJ, Leung HY (2013) Sprouty2, PTEN, and PP2A interact to regulate prostate cancer progression. The Journal of clinical investigation 123**:** 1157-1175

Shim K, Minowada G, Coling DE, Martin GR (2005) Sprouty2, a mouse deafness gene, regulates cell fate decisions in the auditory sensory epithelium by antagonizing FGF signaling. Developmental cell 8**:** 553-564

Sramkoski RM, Pretlow TG, 2nd, Giaconia JM, Pretlow TP, Schwartz S, Sy MS, Marengo SR, Rhim JS, Zhang D, Jacobberger JW (1999) A new human prostate carcinoma cell line, 22Rv1. In vitro cellular & developmental biology Animal 35**:** 403-409

Subramanian A, Tamayo P, Mootha VK, Mukherjee S, Ebert BL, Gillette MA, Paulovich A, Pomeroy SL, Golub TR, Lander ES, Mesirov JP (2005) Gene set enrichment analysis: a knowledge-based approach for interpreting genome-wide expression profiles. Proceedings of the National Academy of Sciences of the United States of America 102**:** 15545-15550

Tan SS, Ahmad I, Bennett HL, Singh L, Nixon C, Seywright M, Barnetson RJ, Edwards J, Leung HY (2011) GRP78 up-regulation is associated with androgen receptor status, Hsp70-Hsp90 client proteins and castrate-resistant prostate cancer. The Journal of pathology 223**:** 81-87

Taurino C, Miller WH, McBride MW, McClure JD, Khanin R, Moreno MU, Dymott JA, Delles C, Dominiczak AF (2010) Gene expression profiling in whole blood of patients with coronary artery disease. Clinical science 119**:** 335-343

Weng Y, Xie F, Xu L, Zagorevski D, Spink DC, Ding X (2010) Analysis of testosterone and dihydrotestosterone in mouse tissues by liquid chromatography-electrospray ionization-tandem mass spectrometry. Analytical biochemistry 402**:** 121-128

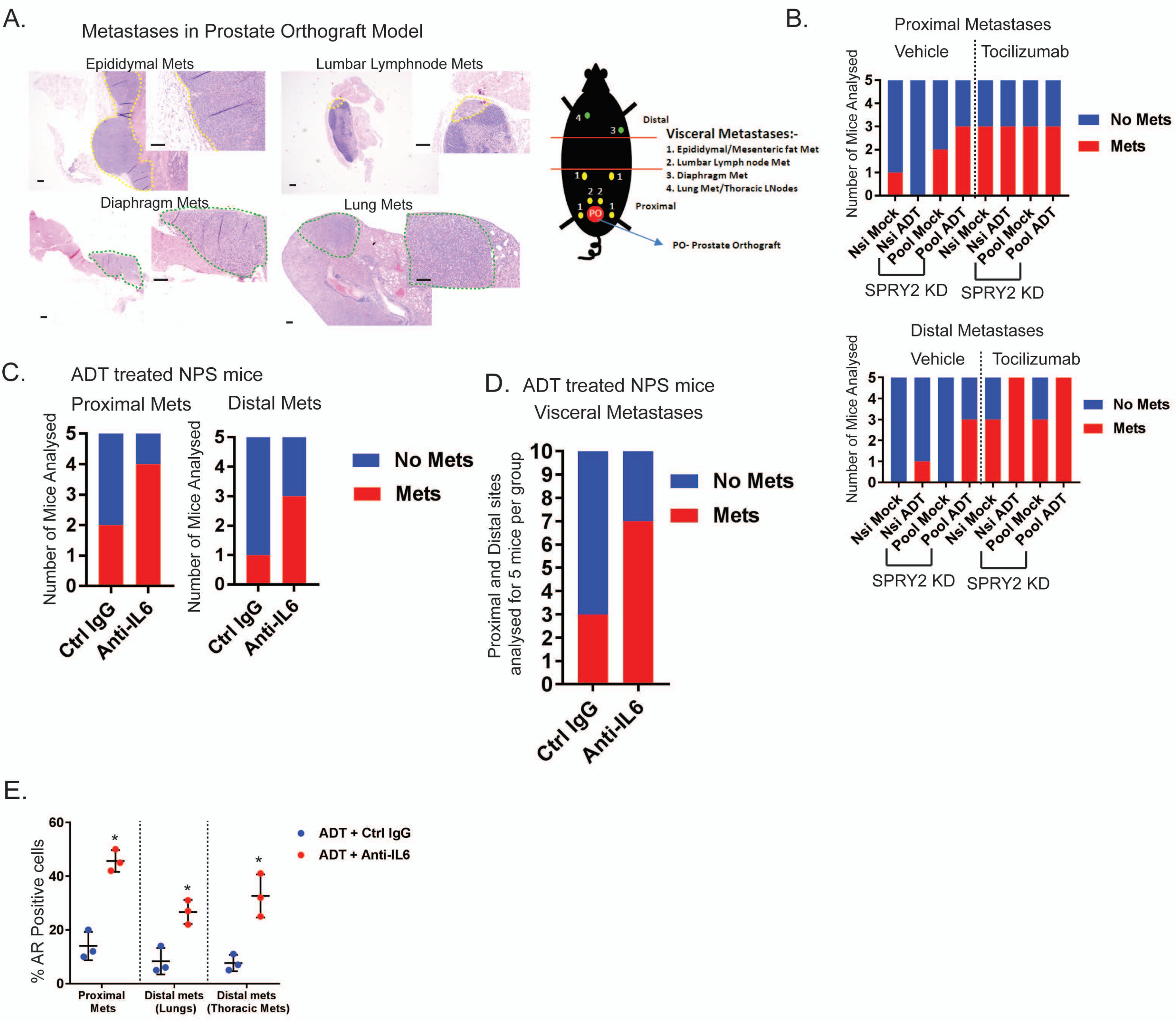

Appendix Figure S1

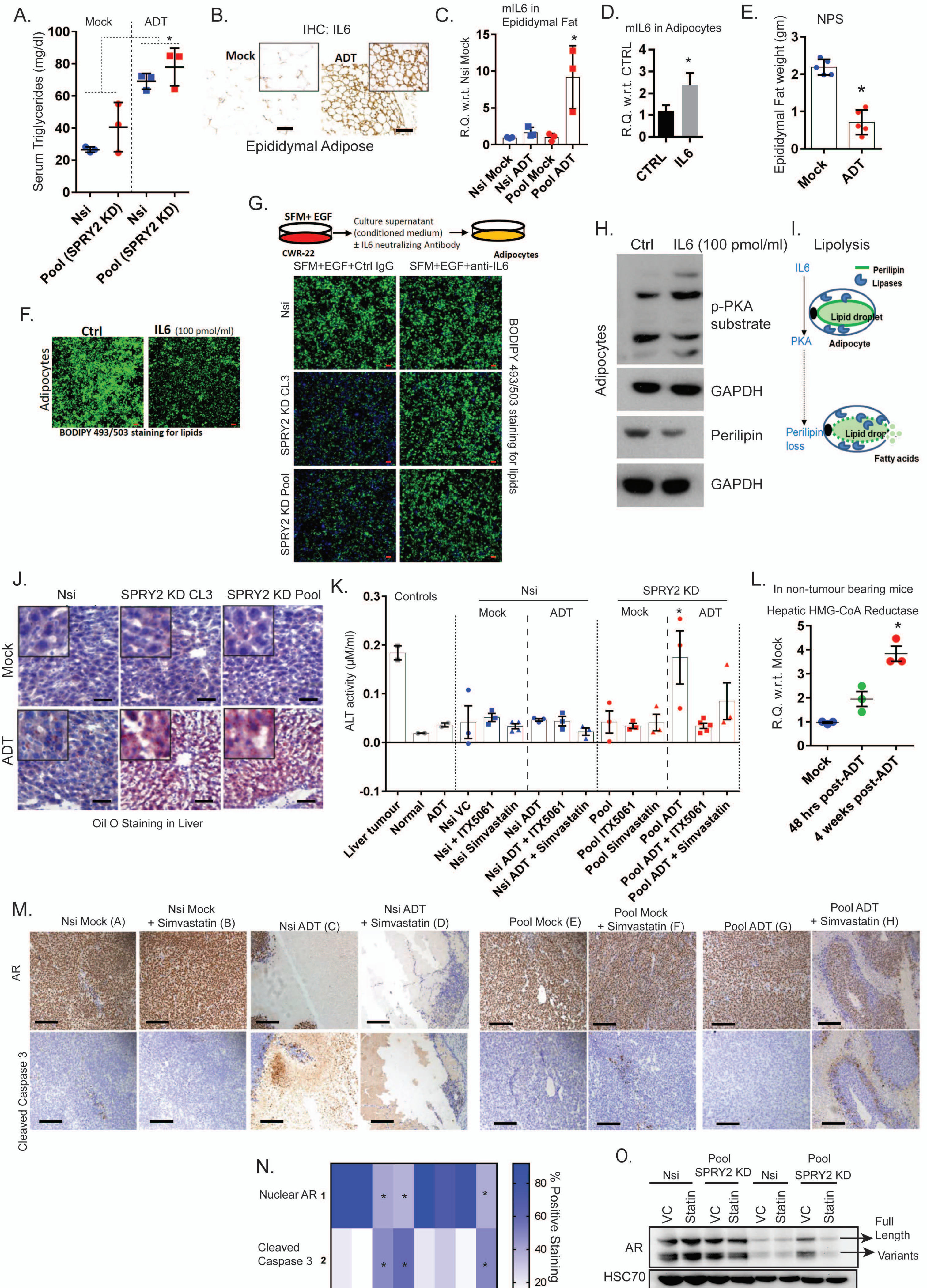

Appendix Figure S2

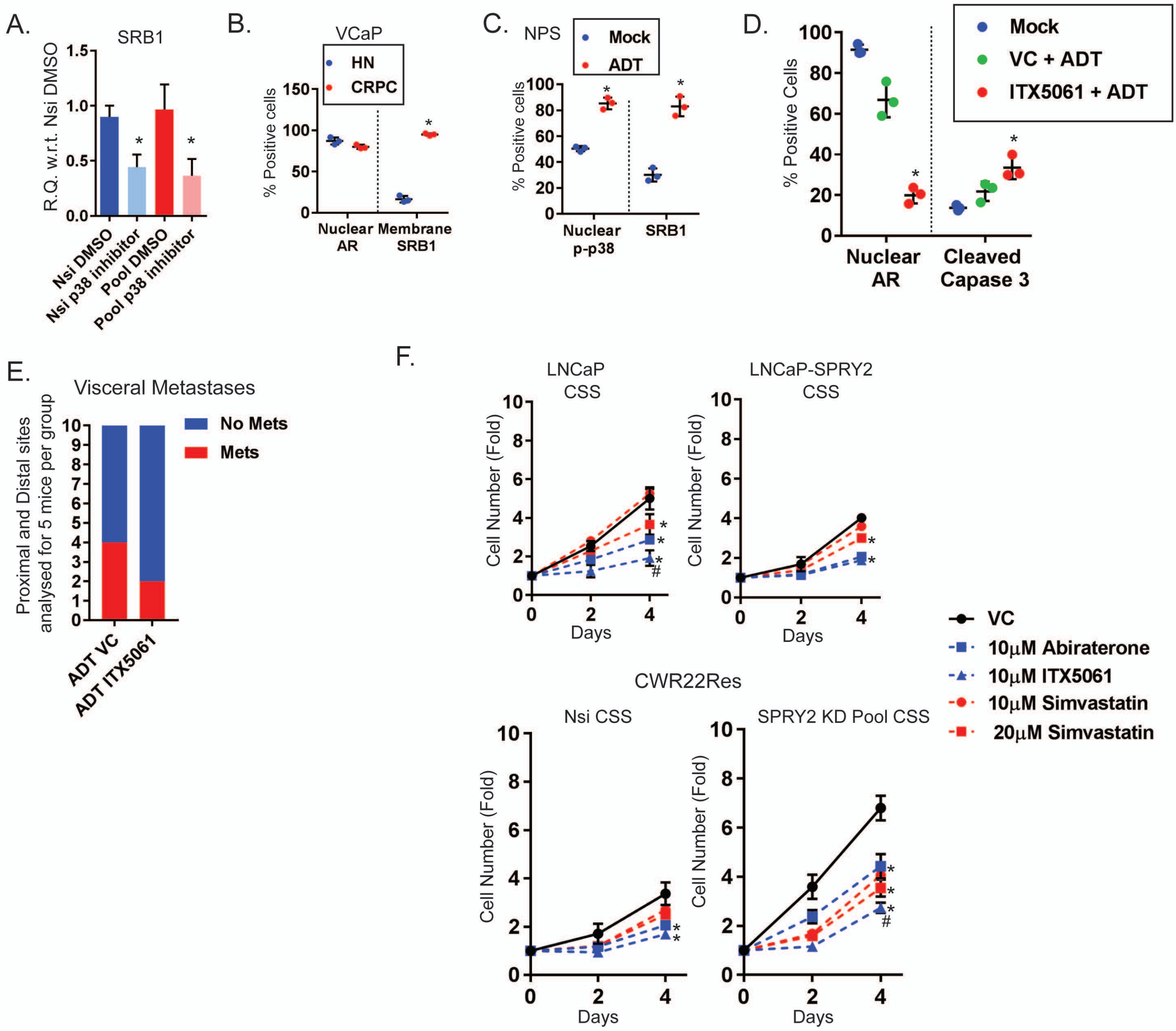

Appendix Figure S3

Appendix Figure S1- Visceral Metastases

A Presentation of visceral metastases observed in the prostate orthograft model. The metastatic incidence is segregated as proximal (yellow dots) or distal (green dots). Representative H&E images of the visceral metastases. Scale bar: 10µm.

B Proximal (upper panel) and distal (lower panel: Chi-square test p=0.002) metastases incidence in the mice with indicated CWR22Res orthografts (n= 5 mice per group).

C Incidence of proximal and distal metastases incidence in ADT treated NPS mice (n= 5 mice per group).

D Incidence of cumulative visceral (proximal and distal) metastases was analysed from 10 sites per group (2 sites: proximal and distal x 5 mice per group) in the NPS. The proximal metastases sites analysed were lumbar lymph nodes and epididymal or mesenteric adipose tissue & the distal sites include thoracic lymph nodes and lungs. Chi-square test: p=0.02.

E IHC quantification of AR positive cells as presented in figure 3K (n=3 mice analysed per group; *p<0.05 compared to respective controls; unpaired two-tailed Student's t-test).

Data Information: In (E), each data point represents one independent observation and the data is presented as mean ± SD. In (B-D), data are presented as contingency graph.

Appendix Figure S2- Systemic IL6 and cholesterol homeostasis in CRPC

A Serum triglyceride levels in mice with CWR22Res prostate orthografts as indicated (n=3 mice per group; *p<0.05 ANOVA Tukey's test).

B Representative immunostained images (n=3) for IL6 of epididymal adipose tissue from ADT treated NPS mice.

C Relative quantitation of murine IL6 mRNA in epididymal adipose tissues of indicated mice with CWR22Res orthografts (n=3 mice per group; *p<0.05 ANOVA Tukey's test).

D Relative quantitation of murine IL6 mRNA in 3T3-L1 adipocytes treated for 16 hr with human 100 pmol/ml IL6 (n=3; *p<0.05; Unpaired two-tailed Student's t-test).

E The epididymal fat weights of NPS mice (n=5 mice per group; *p<0.05 unpaired two-tailed Student's t-test).

F Representative confocal images (n=5) of Bodipy staining in IL6 (100 pmol/ml) treated 3T3-L1 adipocytes.

G Top: Schematic of experimental design for the data represented below. Briefly, conditioned medium from EGF treated CWR22Res cells in serum-free RPMI medium (SFM) with or without anti-IL6 neutralising antibody is added to 3T3-L1 adipocytes pre-stained with Bodipy to test the effects of IL6 on adipocyte lipolysis.

Bottom: Representative confocal images (n=3) of Bodipy (for lipids) and DAPI (for nuclei to account for the presence of cells) staining in 3T3-L1 adipocytes treated for 24 hr with filtered cultured supernatant from Nsi and SPRY2 KD (CL3 and Pool) CWR22Res cells grown in SFM containing EGF (20 ng/ml) for 24 hr with Ctrl or anti-IL6 neutralising antibody (0.1 µg/µl).

H Representative images (n=3) of immunoblots for indicated proteins in whole cell lysates of 3T3-L1 adipocytes treated with IL6 (100 pmol/ml). GAPDH is used as loading control.

I Schematic representation of lipolytic effects of IL6 on adipocytes: IL6 via PKA activation induces loss of perilipin. Normally perilipin forms a protective layer around lipid droplets and protects triglycerides and cholesterol esters with in the lipid droplet from cytosolic lipases. Upon perilipin loss, lipases can induce lipolysis within the cells.

J Representative images of Oil O stained liver sections from mice with CWR22Res prostate orthografts.

K Serum alanine transaminase activity (ALT) in mice bearing CWR22Res orthografts treated as indicated (n=3 mice per group). Sera from mice bearing liver tumours (age associated tumours in wildtype colony mice) were used as a positive control (n=2 mice). Sera from mock or ADT treated non-tumour bearing CD-1 nude mice (age matched) were used as additional controls (n=2 mice). *$p<0.05$; ANOVA Tukey's test.

L Relative quantitation of murine Hmgcr mRNA in murine livers from non-tumour bearing mice treated as indicated (n=3 mice per group; *$p<0.05$ ANOVA with Tukey's test).

M Representative images (n=3) of CWR22Res prostate orthograft sections immunostained for AR and cleaved caspase 3 from Mock or ADT treated mice. Scale bar=10 µm.

N Heat map showing mean value of IHC quantifications in appendix figure S2M (n=3 mice per group; *$p<0.05$ ANOVA Tukey's test).

O Representative immunoblot images for indicated proteins in lysates from indicated CWR22Res orthografts. HSC70 is used as loading control.

Data Information: In (A, C, E, K, L), each data point represents one independent observation. In (A, C, D, E, K), the data is presented as mean ± SD. In (N), data are presented as heat map based on the mean of three independent observations.

Appendix Figure S3- Targeting SRB1 in CRPC

A Relative quantitation of SRB1 mRNA in Nsi and SPRY2 (Pool) CWR22Res cells treated for 16 hr with p38 inhibitor SB203580 (20 µM) (n=3; *p<0.05 compared to respective DMSO controls; unpaired two-tailed Student's t-test).

B-C IHC quantifications of indicated proteins shown in (**B**) figure 5G and (**C**) figure 5H (n=3 mice per group; *p<0.05 compared to respective HN samples; unpaired two-tailed Student's t-test).

D IHC quantifications of indicated proteins shown in figure 6G (n=3 mice per group; *p<0.05 ANOVA Tukey's test).

E Incidence of cumulative visceral (proximal and distal as shown in Appendix S1A) metastases are analysed from 10 sites per group (2 sites x 5 mice per group) in the NPS mice.

F Growth rate of indicated prostate cancer cells relative to Day 0 (T0) in medium containing 10% CSS (hormone deprived conditions) (n=3; *p<0.05 compared to respective DMSO/VC; #p<0.05 compared to all treatments; ANOVA Tukey's test).

Data Information: In (B-D), each data point represents one independent observation. In (F), each data point is presented as mean of three independent observations and data presented as mean ± SD. In (A), the data is presented as mean ± SD. In (E), data are presented contingency graph.

Appendix Table S1- STR analyses of CWR22Res and CWR22RV1 cells.

Appendix Table S2- Detailed statistical analyses and p values for groups compared.

Appendix Table S1

Cell lines	Markers	Allele 1	Allele 2	Allele 3
CWR22Res	AMEL	X	Y	
	CSF1PO	10	11	
	D13S317	9	12	
	D16S539	12		
	D21S11	30		
	D5S818	11	12	13
	D7S820	9	10	
	TH01	6	9.3	
	TPOX	8		
	vWA	15	21	
CWR22RV1	AMEL	X	Y	
	CSF1PO	10	11	
	D13S317	9	12	
	D16S539	12		
	D21S11	30		
	D5S818	11	12	
	D7S820	10	11	
	TH01	6	9.3	
	TPOX	8		
	vWA	15	21	

Results

CWR22Res and CWR22RV1 Matched >95% with 22Rv1 (prostate epithelial)

Appendix Table S2

Figure number	Statistical test used	Experimental groups compared	P Value
Figure 1H	ANOVA Tukey's Test	Time 0	
		CTRL FBS vs. SPRY2 FBS	>0.9999
		CTRL FBS vs. CTRL CSS	>0.9999
		CTRL FBS vs. SPRY2 CSS	>0.9999
		SPRY2 FBS vs. CTRL CSS	>0.9999
		SPRY2 FBS vs. SPRY2 CSS	>0.9999
		CTRL CSS vs. SPRY2 CSS	>0.9999
		Time 2 days	
		CTRL FBS vs. SPRY2 FBS	0.0401
		CTRL FBS vs. CTRL CSS	0.0061
		CTRL FBS vs. SPRY2 CSS	<0.0001
		SPRY2 FBS vs. CTRL CSS	0.8457
		SPRY2 FBS vs. SPRY2 CSS	<0.0001
		CTRL CSS vs. SPRY2 CSS	0.0002
		Tme 4 days	
		CTRL FBS vs. SPRY2 FBS	0.0001
		CTRL FBS vs. CTRL CSS	<0.0001
		CTRL FBS vs. SPRY2 CSS	<0.0001
		SPRY2 FBS vs. CTRL CSS	0.0004
		SPRY2 FBS vs. SPRY2 CSS	<0.0001
		CTRL CSS vs. SPRY2 CSS	<0.0001

Figure number	Statistical test used	Experimental groups compared	P Value
Figure 1I	ANOVA Tukey's Test	Time 0	
		Nsi FBS vs. SPRY2 KD CL3 FBS	>0.9999
		Nsi FBS vs. SPRY2 KD Pool FBS	>0.9999
		Nsi FBS vs. Nsi CSS	>0.9999
		Nsi FBS vs. SPRY2 KD CL3 CSS	>0.9999
		Nsi FBS vs. SPRY2 KD Pool CSS	>0.9999
		SPRY2 KD CL3 FBS vs. SPRY2 KD Pool FBS	>0.9999
		SPRY2 KD CL3 FBS vs. Nsi CSS	>0.9999
		SPRY2 KD CL3 FBS vs. SPRY2 KD CL3 CSS	>0.9999
		SPRY2 KD CL3 FBS vs. SPRY2 KD Pool CSS	>0.9999
		SPRY2 KD Pool FBS vs. Nsi CSS	>0.9999
		SPRY2 KD Pool FBS vs. SPRY2 KD CL3 CSS	>0.9999
		SPRY2 KD Pool FBS vs. SPRY2 KD Pool CSS	>0.9999
		Nsi CSS vs. SPRY2 KD CL3 CSS	>0.9999
		Nsi CSS vs. SPRY2 KD Pool CSS	>0.9999

SPRY2 KD CL3 CSS vs. SPRY2 KD Pool CSS	>0.9999
Time 2 days	
Nsi FBS vs. SPRY2 KD CL3 FBS	0.9614
Nsi FBS vs. SPRY2 KD Pool FBS	0.9961
Nsi FBS vs. Nsi CSS	0.0028
Nsi FBS vs. SPRY2 KD CL3 CSS	0.9951
Nsi FBS vs. SPRY2 KD Pool CSS	>0.9999
SPRY2 KD CL3 FBS vs. SPRY2 KD Pool FBS	0.9994
SPRY2 KD CL3 FBS vs. Nsi CSS	0.0003
SPRY2 KD CL3 FBS vs. SPRY2 KD CL3 CSS	0.9996
SPRY2 KD CL3 FBS vs. SPRY2 KD Pool CSS	0.9675
SPRY2 KD Pool FBS vs. Nsi CSS	0.0007
SPRY2 KD Pool FBS vs. SPRY2 KD CL3 CSS	>0.9999
SPRY2 KD Pool FBS vs. SPRY2 KD Pool CSS	0.9972
Nsi CSS vs. SPRY2 KD CL3 CSS	0.0006
Nsi CSS vs. SPRY2 KD Pool CSS	0.0026
SPRY2 KD CL3 CSS vs. SPRY2 KD Pool CSS	0.9964
Time 4 days	
Nsi FBS vs. SPRY2 KD CL3 FBS	0.4478
Nsi FBS vs. SPRY2 KD Pool FBS	0.7515
Nsi FBS vs. Nsi CSS	<0.0001
Nsi FBS vs. SPRY2 KD CL3 CSS	0.8635
Nsi FBS vs. SPRY2 KD Pool CSS	0.2292
SPRY2 KD CL3 FBS vs. SPRY2 KD Pool FBS	0.0309
SPRY2 KD CL3 FBS vs. Nsi CSS	<0.0001
SPRY2 KD CL3 FBS vs. SPRY2 KD CL3 CSS	0.0528
SPRY2 KD CL3 FBS vs. SPRY2 KD Pool CSS	0.0026
SPRY2 KD Pool FBS vs. Nsi CSS	<0.0001
SPRY2 KD Pool FBS vs. SPRY2 KD CL3 CSS	>0.9999
SPRY2 KD Pool FBS vs. SPRY2 KD Pool CSS	0.9385
Nsi CSS vs. SPRY2 KD CL3 CSS	<0.0001
Nsi CSS vs. SPRY2 KD Pool CSS	<0.0001
SPRY2 KD CL3 CSS vs. SPRY2 KD Pool CSS	0.8599

**Figure number**	**Statistical test used**	**Experimental groups compared**	**P Value**
Figure 1J	ANOVA Tukey's Test	Nsi Mock vs. CL3 Mock	>0.9999
		Nsi Mock vs. Pool Mock	0.9796
		Nsi Mock vs. Nsi ADT	0.0014
		Nsi Mock vs. CL3 ADT	0.9988

		Nsi Mock vs. Pool ADT	0.9999
		CL3 Mock vs. Pool Mock	0.9502
		CL3 Mock vs. Nsi ADT	0.0021
		CL3 Mock vs. CL3 ADT	>0.9999
		CL3 Mock vs. Pool ADT	>0.9999
		Pool Mock vs. Nsi ADT	0.0002
		Pool Mock vs. CL3 ADT	0.8811
		Pool Mock vs. Pool ADT	0.9283
		Nsi ADT vs. CL3 ADT	0.0036
		Nsi ADT vs. Pool ADT	0.0026
		CL3 ADT vs. Pool ADT	>0.9999
Figure 1K	ANOVA Tukey's Test	Nsi Mock vs. CL3 Mock	0.2544
		Nsi Mock vs. Pool Mock	0.7985
		Nsi Mock vs. Nsi ADT	<0.0001
		Nsi Mock vs. CL3 ADT	0.9547
		Nsi Mock vs. Pool ADT	0.9272
		CL3 Mock vs. Pool Mock	0.9207
		CL3 Mock vs. Nsi ADT	<0.0001
		CL3 Mock vs. CL3 ADT	0.7286
		CL3 Mock vs. Pool ADT	0.7873
		Pool Mock vs. Nsi ADT	<0.0001
		Pool Mock vs. CL3 ADT	0.9981
		Pool Mock vs. Pool ADT	0.9995
		Nsi ADT vs. CL3 ADT	<0.0001
		Nsi ADT vs. Pool ADT	<0.0001
		CL3 ADT vs. Pool ADT	>0.9999
Figure 2B	ANOVA Tukey's Test	Nsi Mock vs. CL3 Mock	<0.0001
		Nsi Mock vs. Pool Mock	0.0026
		Nsi Mock vs. Nsi ADT	0.0007
		Nsi Mock vs. CL3 ADT	<0.0001
		Nsi Mock vs. Pool ADT	0.0031
		CL3 Mock vs. Pool Mock	0.0495
		CL3 Mock vs. Nsi ADT	<0.0001
		CL3 Mock vs. CL3 ADT	0.9342
		CL3 Mock vs. Pool ADT	0.0423
		Pool Mock vs. Nsi ADT	<0.0001
		Pool Mock vs. CL3 ADT	0.0058
		Pool Mock vs. Pool ADT	>0.9999
		Nsi ADT vs. CL3 ADT	<0.0001
		Nsi ADT vs. Pool ADT	<0.0001
		CL3 ADT vs. Pool ADT	0.0049

Figure 2E	ANOVA Tukey's Test	Nsi Mock vs. CL3 Mock	0.866
		Nsi Mock vs. Pool Mock	0.9821
		Nsi Mock vs. Nsi ADT	<0.0001
		Nsi Mock vs. CL3 ADT	<0.0001
		Nsi Mock vs. Pool ADT	<0.0001
		CL3 Mock vs. Pool Mock	0.9976
		CL3 Mock vs. Nsi ADT	<0.0001
		CL3 Mock vs. CL3 ADT	<0.0001
		CL3 Mock vs. Pool ADT	<0.0001
		Pool Mock vs. Nsi ADT	<0.0001
		Pool Mock vs. CL3 ADT	<0.0001
		Pool Mock vs. Pool ADT	<0.0001
		Nsi ADT vs. CL3 ADT	>0.9999
		Nsi ADT vs. Pool ADT	0.9904
		CL3 ADT vs. Pool ADT	0.9956
Figure 2F	ANOVA Tukey's Test	Nsi Mock vs. CL3 Mock	>0.9999
		Nsi Mock vs. Pool Mock	0.5422
		Nsi Mock vs. Nsi ADT	<0.0001
		Nsi Mock vs. CL3 ADT	0.0431
		Nsi Mock vs. Pool ADT	0.9653
		CL3 Mock vs. Pool Mock	0.6794
		CL3 Mock vs. Nsi ADT	<0.0001
		CL3 Mock vs. CL3 ADT	0.0695
		CL3 Mock vs. Pool ADT	0.9919
		Pool Mock vs. Nsi ADT	<0.0001
		Pool Mock vs. CL3 ADT	0.6938
		Pool Mock vs. Pool ADT	0.9408
		Nsi ADT vs. CL3 ADT	0.0008
		Nsi ADT vs. Pool ADT	<0.0001
		CL3 ADT vs. Pool ADT	0.2087
Figure 2G	ANOVA Tukey's Test	Nsi Mock vs. CL3 Mock	0.4299
		Nsi Mock vs. Pool Mock	0.9966
		Nsi Mock vs. Nsi ADT	>0.9999
		Nsi Mock vs. CL3 ADT	<0.0001
		Nsi Mock vs. Pool ADT	0.0003
		CL3 Mock vs. Pool Mock	0.6902

		CL3 Mock vs. Nsi ADT	0.357
		CL3 Mock vs. CL3 ADT	0.0004
		CL3 Mock vs. Pool ADT	0.0064
		Pool Mock vs. Nsi ADT	0.9877
		Pool Mock vs. CL3 ADT	<0.0001
		Pool Mock vs. Pool ADT	0.0006
		Nsi ADT vs. CL3 ADT	<0.0001
		Nsi ADT vs. Pool ADT	0.0003
		CL3 ADT vs. Pool ADT	0.4699
Figure 2H	ANOVA Tukey's Test	Nsi Mock vs. CL3 Mock	0.9989
		Nsi Mock vs. Pool Mock	0.264
		Nsi Mock vs. Nsi ADT	>0.9999
		Nsi Mock vs. CL3 ADT	<0.0001
		Nsi Mock vs. Pool ADT	<0.0001
		CL3 Mock vs. Pool Mock	0.4558
		CL3 Mock vs. Nsi ADT	>0.9999
		CL3 Mock vs. CL3 ADT	<0.0001
		CL3 Mock vs. Pool ADT	<0.0001
		Pool Mock vs. Nsi ADT	0.3345
		Pool Mock vs. CL3 ADT	<0.0001
		Pool Mock vs. Pool ADT	<0.0001
		Nsi ADT vs. CL3 ADT	<0.0001
		Nsi ADT vs. Pool ADT	<0.0001
		CL3 ADT vs. Pool ADT	0.9914
Figure 2J	ANOVA Tukey's Test	Nsi	
		Time 0	
		CTRL VC vs. CTRL 10µM Abiraterone	>0.9999
		CTRL VC vs. HSD3B1 KO VC	>0.9999
		CTRL VC vs. HSD3B1 KO 10 µM Abiraterone	>0.9999
		CTRL 10 µM Abiraterone vs. HSD3B1 KO VC	>0.9999
		CTRL 10 µM Abiraterone vs. HSD3B1 KO 10 µM Abiraterone	>0.9999
		HSD3B1 KO VC vs. HSD3B1 KO 10 µM Abiraterone	>0.9999
		Time 2 days	
		CTRL VC vs. CTRL 10 µM Abiraterone	0.066
		CTRL VC vs. HSD3B1 KO VC	0.1582
		CTRL VC vs. HSD3B1 KO 10 µM Abiraterone	0.0865
		CTRL 10 µM Abiraterone vs. HSD3B1 KO VC	0.9692
		CTRL 10 µM Abiraterone vs. HSD3B1 KO 10 µM Abiraterone	0.9991

		HSD3B1 KO VC vs. HSD3B1 KO 10 μM Abiraterone	0.9888
		Time 4 days	
		CTRL VC vs. CTRL 10 μM Abiraterone	<0.0001
		CTRL VC vs. HSD3B1 KO VC	0.0001
		CTRL VC vs. HSD3B1 KO 10 μM Abiraterone	<0.0001
		CTRL 10 μM Abiraterone vs. HSD3B1 KO VC	0.5738
		CTRL 10 μM Abiraterone vs. HSD3B1 KO 10 μM Abiraterone	0.5335
		HSD3B1 KO VC vs. HSD3B1 KO 10 μM Abiraterone	0.0616
		SPRY2 KD Pool	
		Time 0	
		CTRL VC vs. CTRL 10 μM Abiraterone	>0.9999
		CTRL VC vs. HSD3B1 KO VC	>0.9999
		CTRL VC vs. HSD3B1 KO 10 μM Abiraterone	>0.9999
		CTRL 10 μM Abiraterone vs. HSD3B1 KO VC	>0.9999
		CTRL 10 μM Abiraterone vs. HSD3B1 KO 10 μM Abiraterone	>0.9999
		HSD3B1 KO VC vs. HSD3B1 KO 10 μM Abiraterone	>0.9999
		Time 2 days	
		CTRL VC vs. CTRL 10 μM Abiraterone	0.0052
		CTRL VC vs. HSD3B1 KO VC	0.0011
		CTRL VC vs. HSD3B1 KO 10 μM Abiraterone	<0.0001
		CTRL 10 μM Abiraterone vs. HSD3B1 KO VC	0.9233
		CTRL 10 μM Abiraterone vs. HSD3B1 KO 10 μM Abiraterone	0.2245
		HSD3B1 KO VC vs. HSD3B1 KO 10 μM Abiraterone	0.54
		Time 4 days	
		CTRL VC vs. CTRL 10mM Abiraterone	<0.0001
		CTRL VC vs. HSD3B1 KO VC	<0.0001
		CTRL VC vs. HSD3B1 KO 10 μM Abiraterone	<0.0001
		CTRL 10 μM Abiraterone vs. HSD3B1 KO VC	0.54
		CTRL 10 μM Abiraterone vs. HSD3B1 KO 10 μM Abiraterone	<0.0001
		HSD3B1 KO VC vs. HSD3B1 KO 10 μM Abiraterone	0.0002
Figure 2K	Paired t test	CRPC Vs HN	<0.0001
Figure 2L	Unpaired t test Two-tailed	Nsi shHER2 vs Nsi shSc	0.0076

	Unpaired t test Two-tailed	Pool shHER2 vs Pool shSc	0.0089
Figure 2M	ANOVA Tukey's Test	Nsi Mock shScram vs. Nsi ADT shScram	<0.0001
		Nsi Mock shScram vs. Pool Mock shScram	0.4378
		Nsi Mock shScram vs. Pool ADT shScram	0.9433
		Nsi Mock shScram vs. Nsi Mock shHER2	0.5236
		Nsi Mock shScram vs. Nsi ADT shHER2	<0.0001
		Nsi Mock shScram vs. Pool Mock shHER2	0.5647
		Nsi Mock shScram vs. Pool ADT shHER2	0.0001
		Nsi ADT shScram vs. Pool Mock shScram	<0.0001
		Nsi ADT shScram vs. Pool ADT shScram	<0.0001
		Nsi ADT shScram vs. Nsi Mock shHER2	<0.0001
		Nsi ADT shScram vs. Nsi ADT shHER2	0.9999
		Nsi ADT shScram vs. Pool Mock shHER2	<0.0001
		Nsi ADT shScram vs. Pool ADT shHER2	0.5303
		Pool Mock shScram vs. Pool ADT shScram	0.9769
		Pool Mock shScram vs. Nsi Mock shHER2	>0.9999
		Pool Mock shScram vs. Nsi ADT shHER2	<0.0001
		Pool Mock shScram vs. Pool Mock shHER2	>0.9999
		Pool Mock shScram vs. Pool ADT shHER2	<0.0001
		Pool ADT shScram vs. Nsi Mock shHER2	0.9905
		Pool ADT shScram vs. Nsi ADT shHER2	<0.0001
		Pool ADT shScram vs. Pool Mock shHER2	0.9942
		Pool ADT shScram vs. Pool ADT shHER2	<0.0001
		Nsi Mock shHER2 vs. Nsi ADT shHER2	<0.0001
		Nsi Mock shHER2 vs. Pool Mock shHER2	>0.9999
		Nsi Mock shHER2 vs. Pool ADT shHER2	<0.0001
		Nsi ADT shHER2 vs. Pool Mock shHER2	<0.0001
		Nsi ADT shHER2 vs. Pool ADT shHER2	0.2869
		Pool Mock shHER2 vs. Pool ADT shHER2	<0.0001
Figure 3A	ANOVA Tukey's Test	Nsi Mock vs. CL3 Mock	0.5034
		Nsi Mock vs. Pool Mock	0.0457
		Nsi Mock vs. Nsi ADT	>0.9999
		Nsi Mock vs. CL3 ADT	0.0016
		Nsi Mock vs. Pool ADT	<0.0001
		CL3 Mock vs. Pool Mock	0.7465
		CL3 Mock vs. Nsi ADT	0.6387
		CL3 Mock vs. CL3 ADT	0.0966
		CL3 Mock vs. Pool ADT	<0.0001
		Pool Mock vs. Nsi ADT	0.073
		Pool Mock vs. CL3 ADT	0.7209
		Pool Mock vs. Pool ADT	0.0029
		Nsi ADT vs. CL3 ADT	0.0028

		Nsi ADT vs. Pool ADT	<0.0001
		CL3 ADT vs. Pool ADT	0.0743
Figure 3B	ANOVA Tukey's Test	Nsi Mock shScram vs. Nsi ADT shScram	>0.9999
		Nsi Mock shScram vs. Pool Mock shScram	0.0013
		Nsi Mock shScram vs. Pool ADT shScram	<0.0001
		Nsi Mock shScram vs. Nsi Mock shHER2	>0.9999
		Nsi Mock shScram vs. Nsi ADT shHER2	>0.9999
		Nsi Mock shScram vs. Pool Mock shHER2	>0.9999
		Nsi Mock shScram vs. Pool ADT shHER2	0.9972
		Nsi ADT shScram vs. Pool Mock shScram	0.0025
		Nsi ADT shScram vs. Pool ADT shScram	<0.0001
		Nsi ADT shScram vs. Nsi Mock shHER2	0.9993
		Nsi ADT shScram vs. Nsi ADT shHER2	>0.9999
		Nsi ADT shScram vs. Pool Mock shHER2	>0.9999
		Nsi ADT shScram vs. Pool ADT shHER2	>0.9999
		Pool Mock shScram vs. Pool ADT shScram	<0.0001
		Pool Mock shScram vs. Nsi Mock shHER2	0.0009
		Pool Mock shScram vs. Nsi ADT shHER2	0.0023
		Pool Mock shScram vs. Pool Mock shHER2	0.0018
		Pool Mock shScram vs. Pool ADT shHER2	0.0045
		Pool ADT shScram vs. Nsi Mock shHER2	<0.0001
		Pool ADT shScram vs. Nsi ADT shHER2	<0.0001
		Pool ADT shScram vs. Pool Mock shHER2	<0.0001
		Pool ADT shScram vs. Pool ADT shHER2	<0.0001
		Nsi Mock shHER2 vs. Nsi ADT shHER2	0.9997
		Nsi Mock shHER2 vs. Pool Mock shHER2	>0.9999
		Nsi Mock shHER2 vs. Pool ADT shHER2	0.9894
		Nsi ADT shHER2 vs. Pool Mock shHER2	>0.9999
		Nsi ADT shHER2 vs. Pool ADT shHER2	>0.9999
		Pool Mock shHER2 vs. Pool ADT shHER2	0.9997
Figure 3C	Unpaired t test Two-tailed	CWR Nsi p38MAPKi Vs CWR Nsi DMSO	0.0009
		CWR Poolp38MAPKi Vs CWR Pool DMSO	0.004
Figure 3D	Paired t test Two-tailed	CRPC vs HN	0.05
Figure 3E	ANOVA Dunnett's test	Pool ADT Tocilizumab vs. Nsi Mock vehicle	0.0007
		Pool ADT Tocilizumab vs. Nsi ADT vehicle	0.9997
		Pool ADT Tocilizumab vs. Pool Mock vehicle	0.0007
		Pool ADT Tocilizumab vs. Pool ADT vehicle	0.0201

		Pool ADT Tocilizumab vs. Nsi Mock Tocilizumab	0.1408
		Pool ADT Tocilizumab vs. Nsi ADT Tocilizumab	0.9938
		Pool ADT Tocilizumab vs. Pool Mock Tocilizumab	0.0028
		Nsi Mock vehicle vs. Nsi ADT vehicle	0.0004
		Nsi Mock vehicle vs. Pool Mock vehicle	0.9999
		Nsi Mock vehicle vs. Pool ADT vehicle	0.6635
		Nsi Mock vehicle vs. Nsi Mock Tocilizumab	0.1762
		Nsi Mock vehicle vs. Nsi ADT Tocilizumab	0.0002
		Nsi Mock vehicle vs. Pool Mock Tocilizumab	0.9934
		Nsi Mock vehicle vs. Pool ADT Tocilizumab	0.0007
Figure 3F	ANOVA Tukey's Test	Nsi Mock vehicle vs. Nsi ADT vehicle	0.998
		Nsi Mock vehicle vs. Pool Mock vehicle	0.0032
		Nsi Mock vehicle vs. Pool ADT vehicle	<0.0001
		Nsi Mock vehicle vs. Nsi Mock Tocilizumab	>0.9999
		Nsi Mock vehicle vs. Nsi ADT Tocilizumab	0.9971
		Nsi Mock vehicle vs. Pool Mock Tocilizumab	0.9163
		Nsi Mock vehicle vs. Pool ADT Tocilizumab	0.9969
		Nsi ADT vehicle vs. Pool Mock vehicle	0.0005
		Nsi ADT vehicle vs. Pool ADT vehicle	<0.0001
		Nsi ADT vehicle vs. Nsi Mock Tocilizumab	0.9962
		Nsi ADT vehicle vs. Nsi ADT Tocilizumab	>0.9999
		Nsi ADT vehicle vs. Pool Mock Tocilizumab	0.5822
		Nsi ADT vehicle vs. Pool ADT Tocilizumab	>0.9999
		Pool Mock vehicle vs. Pool ADT vehicle	0.0071
		Pool Mock vehicle vs. Nsi Mock Tocilizumab	0.0038
		Pool Mock vehicle vs. Nsi ADT Tocilizumab	0.0005
		Pool Mock vehicle vs. Pool Mock Tocilizumab	0.0707
		Pool Mock vehicle vs. Pool ADT Tocilizumab	0.0005
		Pool ADT vehicle vs. Nsi Mock Tocilizumab	<0.0001
		Pool ADT vehicle vs. Nsi ADT Tocilizumab	<0.0001
		Pool ADT vehicle vs. Pool Mock Tocilizumab	<0.0001
		Pool ADT vehicle vs. Pool ADT Tocilizumab	<0.0001
		Nsi Mock Tocilizumab vs. Nsi ADT Tocilizumab	0.9947
		Nsi Mock Tocilizumab vs. Pool Mock Tocilizumab	0.9371

		Nsi Mock Tocilizumab vs. Pool ADT Tocilizumab	0.9944
		Nsi ADT Tocilizumab vs. Pool Mock Tocilizumab	0.5561
		Nsi ADT Tocilizumab vs. Pool ADT Tocilizumab	>0.9999
		Pool Mock Tocilizumab vs. Pool ADT Tocilizumab	0.5517
Figure 3G	ANOVA Tukey's Test	Nsi Mock Vehicle vs. Nsi ADT Vehicle	0.0021
		Nsi Mock Vehicle vs. Pool Mock Vehicle	0.9879
		Nsi Mock Vehicle vs. Pool ADT Vehicle	0.9183
		Nsi Mock Vehicle vs. Nsi Mock Tocilizumab	>0.9999
		Nsi Mock Vehicle vs. Nsi ADT Tocilizumab	0.0003
		Nsi Mock Vehicle vs. Pool Mock Tocilizumab	0.7335
		Nsi Mock Vehicle vs. Pool ADT Tocilizumab	0.0015
		Nsi ADT Vehicle vs. Pool Mock Vehicle	0.0002
		Nsi ADT Vehicle vs. Pool ADT Vehicle	<0.0001
		Nsi ADT Vehicle vs. Nsi Mock Tocilizumab	0.0042
		Nsi ADT Vehicle vs. Nsi ADT Tocilizumab	0.9959
		Nsi ADT Vehicle vs. Pool Mock Tocilizumab	<0.0001
		Nsi ADT Vehicle vs. Pool ADT Tocilizumab	>0.9999
		Pool Mock Vehicle vs. Pool ADT Vehicle	>0.9999
		Pool Mock Vehicle vs. Nsi Mock Tocilizumab	0.9501
		Pool Mock Vehicle vs. Nsi ADT Tocilizumab	<0.0001
		Pool Mock Vehicle vs. Pool Mock Tocilizumab	0.9938
		Pool Mock Vehicle vs. Pool ADT Tocilizumab	0.0001
		Pool ADT Vehicle vs. Nsi Mock Tocilizumab	0.8135
		Pool ADT Vehicle vs. Nsi ADT Tocilizumab	<0.0001
		Pool ADT Vehicle vs. Pool Mock Tocilizumab	>0.9999
		Pool ADT Vehicle vs. Pool ADT Tocilizumab	<0.0001
		Nsi Mock Tocilizumab vs. Nsi ADT Tocilizumab	0.0006
		Nsi Mock Tocilizumab vs. Pool Mock Tocilizumab	0.5767
		Nsi Mock Tocilizumab vs. Pool ADT Tocilizumab	0.003
		Nsi ADT Tocilizumab vs. Pool Mock Tocilizumab	<0.0001
		Nsi ADT Tocilizumab vs. Pool ADT Tocilizumab	0.9989
		Pool Mock Tocilizumab vs. Pool ADT Tocilizumab	<0.0001
			0.9067
Figure 3H	ANOVA Tukey's Test	Nsi Mock Vehicle vs. Nsi ADT Vehicle	<0.0001

		Nsi Mock Vehicle vs. Pool Mock Vehicle	<0.0001
		Nsi Mock Vehicle vs. Pool ADT Vehicle	0.9999
		Nsi Mock Vehicle vs. Nsi Mock Tocilizumab	0.9113
		Nsi Mock Vehicle vs. Nsi ADT Tocilizumab	0.8805
		Nsi Mock Vehicle vs. Pool Mock Tocilizumab	>0.9999
		Nsi Mock Vehicle vs. Pool ADT Tocilizumab	<0.0001
		Nsi ADT Vehicle vs. Pool Mock Vehicle	<0.0001
		Nsi ADT Vehicle vs. Pool ADT Vehicle	0.992
		Nsi ADT Vehicle vs. Nsi Mock Tocilizumab	>0.9999
		Nsi ADT Vehicle vs. Nsi ADT Tocilizumab	0.2387
		Nsi ADT Vehicle vs. Pool Mock Tocilizumab	0.9612
		Nsi ADT Vehicle vs. Pool ADT Tocilizumab	0.0168
		Pool Mock Vehicle vs. Pool ADT Vehicle	<0.0001
		Pool Mock Vehicle vs. Nsi Mock Tocilizumab	<0.0001
		Pool Mock Vehicle vs. Nsi ADT Tocilizumab	<0.0001
		Pool Mock Vehicle vs. Pool Mock Tocilizumab	<0.0001
		Pool Mock Vehicle vs. Pool ADT Tocilizumab	<0.0001
		Pool ADT Vehicle vs. Nsi Mock Tocilizumab	<0.0001
		Pool ADT Vehicle vs. Nsi ADT Tocilizumab	<0.0001
		Pool ADT Vehicle vs. Pool Mock Tocilizumab	<0.0001
		Pool ADT Vehicle vs. Pool ADT Tocilizumab	0.9928
		Nsi Mock Tocilizumab vs. Nsi ADT Tocilizumab	0.6983
		Nsi Mock Tocilizumab vs. Pool Mock Tocilizumab	>0.9999
		Nsi Mock Tocilizumab vs. Pool ADT Tocilizumab	0.2442
		Nsi ADT Tocilizumab vs. Pool Mock Tocilizumab	0.9636
		Nsi ADT Tocilizumab vs. Pool ADT Tocilizumab	0.84
		Pool Mock Tocilizumab vs. Pool ADT Tocilizumab	
Figure 3I	Unpaired t test Two-tailed	Anti-IL6 ADT vs Ctrl IgG ADT	0.0003
Figure 3J	Chi-square test	Metastatic incidence in Tocilizumab treated set	<0.001
Figure 3L	ANOVA Tukey's Test	Nsi Mock Vehicle vs. Nsi ADT Vehicle	0.5382
		Nsi Mock Vehicle vs. Pool Mock Vehicle	0.6282
		Nsi Mock Vehicle vs. Pool ADT Vehicle	<0.0001
		Nsi Mock Vehicle vs. Nsi Mock	>0.9999

		Tocilizumab	
		Nsi Mock Vehicle vs. Nsi ADT Tocilizumab	0.9958
		Nsi Mock Vehicle vs. Pool Mock Tocilizumab	0.9988
		Nsi Mock Vehicle vs. Pool ADT Tocilizumab	>0.9999
		Nsi ADT Vehicle vs. Pool Mock Vehicle	0.0288
		Nsi ADT Vehicle vs. Pool ADT Vehicle	<0.0001
		Nsi ADT Vehicle vs. Nsi Mock Tocilizumab	0.642
		Nsi ADT Vehicle vs. Nsi ADT Tocilizumab	0.9032
		Nsi ADT Vehicle vs. Pool Mock Tocilizumab	0.2525
		Nsi ADT Vehicle vs. Pool ADT Tocilizumab	0.3721
		Pool Mock Vehicle vs. Pool ADT Vehicle	<0.0001
		Pool Mock Vehicle vs. Nsi Mock Tocilizumab	0.5246
		Pool Mock Vehicle vs. Nsi ADT Tocilizumab	0.2621
		Pool Mock Vehicle vs. Pool Mock Tocilizumab	0.9115
		Pool Mock Vehicle vs. Pool ADT Tocilizumab	0.7977
		Pool ADT Vehicle vs. Nsi Mock Tocilizumab	<0.0001
		Pool ADT Vehicle vs. Nsi ADT Tocilizumab	<0.0001
		Pool ADT Vehicle vs. Pool Mock Tocilizumab	<0.0001
		Pool ADT Vehicle vs. Pool ADT Tocilizumab	<0.0001
		Nsi Mock Tocilizumab vs. Nsi ADT Tocilizumab	0.9993
		Nsi Mock Tocilizumab vs. Pool Mock Tocilizumab	0.9937
		Nsi Mock Tocilizumab vs. Pool ADT Tocilizumab	0.9996
		Nsi ADT Tocilizumab vs. Pool Mock Tocilizumab	0.8965
		Nsi ADT Tocilizumab vs. Pool ADT Tocilizumab	0.9675
		Pool Mock Tocilizumab vs. Pool ADT Tocilizumab	>0.9999
Figure 4A	ANOVA Tukey's Test	Nsi Mock vs. CL3 Mock	0.9052
		Nsi Mock vs. Pool Mock	0.0367
		Nsi Mock vs. Nsi ADT	0.0002
		Nsi Mock vs. CL3 ADT	0.0005
		Nsi Mock vs. Pool ADT	0.0012
		CL3 Mock vs. Pool Mock	0.1895
		CL3 Mock vs. Nsi ADT	0.0008
		CL3 Mock vs. CL3 ADT	0.0023
		CL3 Mock vs. Pool ADT	0.0063
		Pool Mock vs. Nsi ADT	0.0513
		Pool Mock vs. CL3 ADT	0.1476
		Pool Mock vs. Pool ADT	0.3661

		Nsi ADT vs. CL3 ADT	0.9844
		Nsi ADT vs. Pool ADT	0.7888
		CL3 ADT vs. Pool ADT	0.9861
Figure 4B	ANOVA Tukey's Test	Nsi Mock vs. CL3 Mock	>0.9999
		Nsi Mock vs. Pool Mock	>0.9999
		Nsi Mock vs. Nsi ADT	>0.9999
		Nsi Mock vs. CL3 ADT	<0.0001
		Nsi Mock vs. Pool ADT	<0.0001
		CL3 Mock vs. Pool Mock	>0.9999
		CL3 Mock vs. Nsi ADT	>0.9999
		CL3 Mock vs. CL3 ADT	<0.0001
		CL3 Mock vs. Pool ADT	<0.0001
		Pool Mock vs. Nsi ADT	0.9991
		Pool Mock vs. CL3 ADT	<0.0001
		Pool Mock vs. Pool ADT	<0.0001
		Nsi ADT vs. CL3 ADT	<0.0001
		Nsi ADT vs. Pool ADT	<0.0001
		CL3 ADT vs. Pool ADT	0.9498
Figure 4C	ANOVA Tukey's Test	Nsi Mock vs. CL3 Mock	>0.9999
		Nsi Mock vs. Pool Mock	0.9951
		Nsi Mock vs. Nsi ADT	0.9921
		Nsi Mock vs. CL3 ADT	0.0026
		Nsi Mock vs. Pool ADT	0.0038
		CL3 Mock vs. Pool Mock	0.9996
		CL3 Mock vs. Nsi ADT	0.9991
		CL3 Mock vs. CL3 ADT	0.0015
		CL3 Mock vs. Pool ADT	0.0023
		Pool Mock vs. Nsi ADT	>0.9999
		Pool Mock vs. CL3 ADT	0.0007
		Pool Mock vs. Pool ADT	0.0011
		Nsi ADT vs. CL3 ADT	0.0006
		Nsi ADT vs. Pool ADT	0.0009
		CL3 ADT vs. Pool ADT	>0.9999
Figure 4D	ANOVA Tukey's Test	Nsi Mock Vehicle vs. Nsi ADT Vehicle	0.9873
		Nsi Mock Vehicle vs. Pool Mock Vehicle	0.9996
		Nsi Mock Vehicle vs. Pool ADT Vehicle	<0.0001
		Nsi Mock Vehicle vs. Nsi Mock Tocilizumab	>0.9999
		Nsi Mock Vehicle vs. Nsi ADT Tocilizumab	>0.9999
		Nsi Mock Vehicle vs. Pool Mock Tocilizumab	0.9745
		Nsi Mock Vehicle vs. Pool ADT Tocilizumab	0.3934
		Nsi ADT Vehicle vs. Pool Mock Vehicle	>0.9999

		Nsi ADT Vehicle vs. Pool ADT Vehicle	<0.0001
		Nsi ADT Vehicle vs. Nsi Mock Tocilizumab	0.9612
		Nsi ADT Vehicle vs. Nsi ADT Tocilizumab	0.9994
		Nsi ADT Vehicle vs. Pool Mock Tocilizumab	>0.9999
		Nsi ADT Vehicle vs. Pool ADT Tocilizumab	0.079
		Pool Mock Vehicle vs. Pool ADT Vehicle	<0.0001
		Pool Mock Vehicle vs. Nsi Mock Tocilizumab	0.9965
		Pool Mock Vehicle vs. Nsi ADT Tocilizumab	>0.9999
		Pool Mock Vehicle vs. Pool Mock Tocilizumab	0.9997
		Pool Mock Vehicle vs. Pool ADT Tocilizumab	0.1661
		Pool ADT Vehicle vs. Nsi Mock Tocilizumab	<0.0001
		Pool ADT Vehicle vs. Nsi ADT Tocilizumab	<0.0001
		Pool ADT Vehicle vs. Pool Mock Tocilizumab	<0.0001
		Pool ADT Vehicle vs. Pool ADT Tocilizumab	<0.0001
		Nsi Mock Tocilizumab vs. Nsi ADT Tocilizumab	0.9994
		Nsi Mock Tocilizumab vs. Pool Mock Tocilizumab	0.9348
		Nsi Mock Tocilizumab vs. Pool ADT Tocilizumab	0.5095
		Nsi ADT Tocilizumab vs. Pool Mock Tocilizumab	0.9979
		Nsi ADT Tocilizumab vs. Pool ADT Tocilizumab	0.228
		Pool Mock Tocilizumab vs. Pool ADT Tocilizumab	0.0613
Figure 4E	ANOVA Tukey's Test	Nsi Mock Vehicle vs. Nsi ADT Vehicle	0.9963
		Nsi Mock Vehicle vs. Pool Mock Vehicle	>0.9999
		Nsi Mock Vehicle vs. Pool ADT Vehicle	0.0004
		Nsi Mock Vehicle vs. Nsi Mock Tocilizumab	>0.9999
		Nsi Mock Vehicle vs. Nsi ADT Tocilizumab	>0.9999
		Nsi Mock Vehicle vs. Pool Mock Tocilizumab	0.9998
		Nsi Mock Vehicle vs. Pool ADT Tocilizumab	0.834
		Nsi ADT Vehicle vs. Pool Mock Vehicle	0.9962
		Nsi ADT Vehicle vs. Pool ADT Vehicle	0.003
		Nsi ADT Vehicle vs. Nsi Mock Tocilizumab	0.9843
		Nsi ADT Vehicle vs. Nsi ADT Tocilizumab	0.9998
		Nsi ADT Vehicle vs. Pool Mock Tocilizumab	>0.9999
		Nsi ADT Vehicle vs. Pool ADT Tocilizumab	0.9953
		Pool Mock Vehicle vs. Pool ADT Vehicle	0.0004
		Pool Mock Vehicle vs. Nsi Mock Tocilizumab	>0.9999
		Pool Mock Vehicle vs. Nsi ADT Tocilizumab	>0.9999
		Pool Mock Vehicle vs. Pool Mock Tocilizumab	0.9998
		Pool Mock Vehicle vs. Pool ADT Tocilizumab	0.8328
		Pool ADT Vehicle vs. Nsi Mock Tocilizumab	0.0002
		Pool ADT Vehicle vs. Nsi ADT Tocilizumab	0.0009

		Pool ADT Vehicle vs. Pool Mock Tocilizumab	0.0015
		Pool ADT Vehicle vs. Pool ADT Tocilizumab	0.0207
		Nsi Mock Tocilizumab vs. Nsi ADT Tocilizumab	0.9998
		Nsi Mock Tocilizumab vs. Pool Mock Tocilizumab	0.998
		Nsi Mock Tocilizumab vs. Pool ADT Tocilizumab	0.731
		Nsi ADT Tocilizumab vs. Pool Mock Tocilizumab	>0.9999
		Nsi ADT Tocilizumab vs. Pool ADT Tocilizumab	0.9358
		Pool Mock Tocilizumab vs. Pool ADT Tocilizumab	0.9727
Figure 4F	Unpaired t test Two-tailed	CRPC vs HN	0.0002
Figure 4G	Pearson r	IL6 vs. PSA	< 0.0001
Figure 4I	ANOVA Tukey's Test	Nsi Mock vs. CL3 Mock	>0.9999
		Nsi Mock vs. Pool Mock	0.9905
		Nsi Mock vs. Nsi ADT	0.9824
		Nsi Mock vs. Pool ADT	<0.0001
		Nsi Mock vs. CL3 ADT	<0.0001
		CL3 Mock vs. Pool Mock	0.9968
		CL3 Mock vs. Nsi ADT	0.9639
		CL3 Mock vs. Pool ADT	<0.0001
		CL3 Mock vs. CL3 ADT	<0.0001
		Pool Mock vs. Nsi ADT	0.7953
		Pool Mock vs. Pool ADT	<0.0001
		Pool Mock vs. CL3 ADT	<0.0001
		Nsi ADT vs. Pool ADT	<0.0001
		Nsi ADT vs. CL3 ADT	<0.0001
		Pool ADT vs. CL3 ADT	0.9994
Figure 4J	ANOVA Tukey's Test	Nsi Mock Vehicle vs. Nsi ADT Vehicle	<0.0001
		Nsi Mock Vehicle vs. Pool Mock Vehicle	0.9694
		Nsi Mock Vehicle vs. Pool ADT Vehicle	<0.0001
		Nsi Mock Vehicle vs. Nsi Mock Tocilizumab	0.2392
		Nsi Mock Vehicle vs. Nsi ADT Tocilizumab	0.2652
		Nsi Mock Vehicle vs. Pool Mock Tocilizumab	0.6724
		Nsi Mock Vehicle vs. Pool ADT Tocilizumab	0.9985
		Nsi ADT Vehicle vs. Pool Mock Vehicle	<0.0001
		Nsi ADT Vehicle vs. Pool ADT Vehicle	<0.0001
		Nsi ADT Vehicle vs. Nsi Mock Tocilizumab	<0.0001

		Nsi ADT Vehicle vs. Nsi ADT Tocilizumab	0.0482
		Nsi ADT Vehicle vs. Pool Mock Tocilizumab	<0.0001
		Nsi ADT Vehicle vs. Pool ADT Tocilizumab	0.0004
		Pool Mock Vehicle vs. Pool ADT Vehicle	<0.0001
		Pool Mock Vehicle vs. Nsi Mock Tocilizumab	0.8164
		Pool Mock Vehicle vs. Nsi ADT Tocilizumab	0.0316
		Pool Mock Vehicle vs. Pool Mock Tocilizumab	0.996
		Pool Mock Vehicle vs. Pool ADT Tocilizumab	0.7352
		Pool ADT Vehicle vs. Nsi Mock Tocilizumab	<0.0001
		Pool ADT Vehicle vs. Nsi ADT Tocilizumab	<0.0001
		Pool ADT Vehicle vs. Pool Mock Tocilizumab	<0.0001
		Pool ADT Vehicle vs. Pool ADT Tocilizumab	<0.0001
		Nsi Mock Tocilizumab vs. Nsi ADT Tocilizumab	0.0006
		Nsi Mock Tocilizumab vs. Pool Mock Tocilizumab	0.9941
		Nsi Mock Tocilizumab vs. Pool ADT Tocilizumab	0.0701
		Nsi ADT Tocilizumab vs. Pool Mock Tocilizumab	0.0051
		Nsi ADT Tocilizumab vs. Pool ADT Tocilizumab	0.6162
		Pool Mock Tocilizumab vs. Pool ADT Tocilizumab	0.3076
Figure 4L	ANOVA Tukey's Test	Nsi Mock vs. CL3 Mock	>0.9999
		Nsi Mock vs. Pool Mock	0.9999
		Nsi Mock vs. Nsi ADT	0.9766
		Nsi Mock vs. CL3 ADT	<0.0001
		Nsi Mock vs. Pool ADT	<0.0001
		CL3 Mock vs. Pool Mock	>0.9999
		CL3 Mock vs. Nsi ADT	0.9789
		CL3 Mock vs. CL3 ADT	<0.0001
		CL3 Mock vs. Pool ADT	<0.0001
		Pool Mock vs. Nsi ADT	0.9959
		Pool Mock vs. CL3 ADT	<0.0001
		Pool Mock vs. Pool ADT	<0.0001
		Nsi ADT vs. CL3 ADT	<0.0001
		Nsi ADT vs. Pool ADT	<0.0001
		CL3 ADT vs. Pool ADT	0.9929
Figure 4M	ANOVA Tukey's Test	Nsi Mock Vehicle vs. Nsi ADT Vehicle	0.0694
		Nsi Mock Vehicle vs. Pool Mock Vehicle	0.4763
		Nsi Mock Vehicle vs. Pool ADT Vehicle	<0.0001
		Nsi Mock Vehicle vs. Nsi Mock Tocilizumab	0.9974
		Nsi Mock Vehicle vs. Nsi ADT Tocilizumab	0.1745
		Nsi Mock Vehicle vs. Pool Mock Tocilizumab	0.9855
		Nsi Mock Vehicle vs. Pool ADT Tocilizumab	0.003

		Nsi ADT Vehicle vs. Pool Mock Vehicle	0.9603
		Nsi ADT Vehicle vs. Pool ADT Vehicle	<0.0001
		Nsi ADT Vehicle vs. Nsi Mock Tocilizumab	0.014
		Nsi ADT Vehicle vs. Nsi ADT Tocilizumab	0.9998
		Nsi ADT Vehicle vs. Pool Mock Tocilizumab	0.3721
		Nsi ADT Vehicle vs. Pool ADT Tocilizumab	0.914
		Pool Mock Vehicle vs. Pool ADT Vehicle	<0.0001
		Pool Mock Vehicle vs. Nsi Mock Tocilizumab	0.1602
		Pool Mock Vehicle vs. Nsi ADT Tocilizumab	0.9983
		Pool Mock Vehicle vs. Pool Mock Tocilizumab	0.9401
		Pool Mock Vehicle vs. Pool ADT Tocilizumab	0.3247
		Pool ADT Vehicle vs. Nsi Mock Tocilizumab	<0.0001
		Pool ADT Vehicle vs. Nsi ADT Tocilizumab	<0.0001
		Pool ADT Vehicle vs. Pool Mock Tocilizumab	<0.0001
		Pool ADT Vehicle vs. Pool ADT Tocilizumab	<0.0001
		Nsi Mock Tocilizumab vs. Nsi ADT Tocilizumab	0.0417
		Nsi Mock Tocilizumab vs. Pool Mock Tocilizumab	0.7762
		Nsi Mock Tocilizumab vs. Pool ADT Tocilizumab	0.0005
		Nsi ADT Tocilizumab vs. Pool Mock Tocilizumab	0.6443
		Nsi ADT Tocilizumab vs. Pool ADT Tocilizumab	0.6991
		Pool Mock Tocilizumab vs. Pool ADT Tocilizumab	0.0303
Figure 4N	Spearman r	FFA nMol/ul vs. IL6 pMol/ml	0.037
Figure 5A	ANOVA Tukey's Test	Nsi Mock Vehicle vs. Nsi ADT Vehicle	0.6195
		Nsi Mock Vehicle vs. Pool Mock Vehicle	0.9666
		Nsi Mock Vehicle vs. Pool ADT Vehicle	<0.0001
		Nsi Mock Vehicle vs. Nsi Mock Tocilizumab	0.9913
		Nsi Mock Vehicle vs. Nsi ADT Tocilizumab	0.9999
		Nsi Mock Vehicle vs. Pool Mock Tocilizumab	>0.9999
		Nsi Mock Vehicle vs. Pool ADT Tocilizumab	>0.9999
		Nsi ADT Vehicle vs. Pool Mock Vehicle	0.979
		Nsi ADT Vehicle vs. Pool ADT Vehicle	0.0022
		Nsi ADT Vehicle vs. Nsi Mock Tocilizumab	0.2154
		Nsi ADT Vehicle vs. Nsi ADT Tocilizumab	0.4849
		Nsi ADT Vehicle vs. Pool Mock Tocilizumab	0.6614
		Nsi ADT Vehicle vs. Pool ADT Tocilizumab	0.4106
		Pool Mock Vehicle vs. Pool ADT Vehicle	0.0001
		Pool Mock Vehicle vs. Nsi Mock Tocilizumab	0.5914
		Pool Mock Vehicle vs. Nsi ADT Tocilizumab	0.8751
		Pool Mock Vehicle vs. Pool Mock Tocilizumab	0.9779

		Pool Mock Vehicle vs. Pool ADT Tocilizumab	0.87
		Pool ADT Vehicle vs. Nsi Mock Tocilizumab	<0.0001
		Pool ADT Vehicle vs. Nsi ADT Tocilizumab	<0.0001
		Pool ADT Vehicle vs. Pool Mock Tocilizumab	<0.0001
		Pool ADT Vehicle vs. Pool ADT Tocilizumab	<0.0001
		Nsi Mock Tocilizumab vs. Nsi ADT Tocilizumab	>0.9999
		Nsi Mock Tocilizumab vs. Pool Mock Tocilizumab	0.9859
		Nsi Mock Tocilizumab vs. Pool ADT Tocilizumab	0.9981
		Nsi ADT Tocilizumab vs. Pool Mock Tocilizumab	0.9996
		Nsi ADT Tocilizumab vs. Pool ADT Tocilizumab	>0.9999
		Pool Mock Tocilizumab vs. Pool ADT Tocilizumab	>0.9999
Figure 5B	ANOVA Tukey's Test	Nsi Mock Vehicle vs. Nsi ADT Vehicle	0.0003
		Nsi Mock Vehicle vs. Pool Mock Vehicle	0.9821
		Nsi Mock Vehicle vs. Pool ADT Vehicle	>0.9999
		Nsi Mock Vehicle vs. Nsi Mock Simvastatin	0.9957
		Nsi Mock Vehicle vs. Nsi ADT Simvastatin	<0.0001
		Nsi Mock Vehicle vs. Pool Mock Simvastatin	0.3581
		Nsi Mock Vehicle vs. Pool ADT Simvastatin	0.0016
		Nsi ADT Vehicle vs. Pool Mock Vehicle	<0.0001
		Nsi ADT Vehicle vs. Pool ADT Vehicle	0.0002
		Nsi ADT Vehicle vs. Nsi Mock Simvastatin	0.0026
		Nsi ADT Vehicle vs. Nsi ADT Simvastatin	0.0077
		Nsi ADT Vehicle vs. Pool Mock Simvastatin	<0.0001
		Nsi ADT Vehicle vs. Pool ADT Simvastatin	0.9993
		Pool Mock Vehicle vs. Pool ADT Vehicle	0.9982
		Pool Mock Vehicle vs. Nsi Mock Simvastatin	0.7241
		Pool Mock Vehicle vs. Nsi ADT Simvastatin	<0.0001
		Pool Mock Vehicle vs. Pool Mock Simvastatin	0.8849
		Pool Mock Vehicle vs. Pool ADT Simvastatin	0.0001
		Pool ADT Vehicle vs. Nsi Mock Simvastatin	0.969
		Pool ADT Vehicle vs. Nsi ADT Simvastatin	<0.0001
		Pool ADT Vehicle vs. Pool Mock Simvastatin	0.5325
		Pool ADT Vehicle vs. Pool ADT Simvastatin	0.0007
		Nsi Mock Simvastatin vs. Nsi ADT Simvastatin	<0.0001
		Nsi Mock Simvastatin vs. Pool Mock Simvastatin	0.0939
		Nsi Mock Simvastatin vs. Pool ADT Simvastatin	0.0109
		Nsi ADT Simvastatin vs. Pool Mock Simvastatin	<0.0001
		Nsi ADT Simvastatin vs. Pool ADT Simvastatin	0.0018

		Pool Mock Simvastatin vs. Pool ADT Simvastatin	<0.0001
Figure 5E	ANOVA Tukey's Test	Nsi Mock vs. CL3 Mock	0.1827
		Nsi Mock vs. Pool Mock	0.6922
		Nsi Mock vs. Nsi ADT	0.1475
		Nsi Mock vs. CL3 ADT	0.0007
		Nsi Mock vs. Pool ADT	0.0005
		CL3 Mock vs. Pool Mock	0.9197
		CL3 Mock vs. Nsi ADT	0.0005
		CL3 Mock vs. CL3 ADT	0.1816
		CL3 Mock vs. Pool ADT	0.1405
		Pool Mock vs. Nsi ADT	0.006
		Pool Mock vs. CL3 ADT	0.024
		Pool Mock vs. Pool ADT	0.0174
		Nsi ADT vs. CL3 ADT	<0.0001
		Nsi ADT vs. Pool ADT	<0.0001
		CL3 ADT vs. Pool ADT	>0.9999
Figure 5F	ANOVA Tukey's Test	Nsi Mock Vehicle vs. Nsi ADT Vehicle	0.9984
		Nsi Mock Vehicle vs. Pool Mock Vehicle	0.9806
		Nsi Mock Vehicle vs. Pool ADT Vehicle	0.0074
		Nsi Mock Vehicle vs. Nsi Mock Tocilizumab	0.9975
		Nsi Mock Vehicle vs. Nsi ADT Tocilizumab	0.8658
		Nsi Mock Vehicle vs. Pool Mock Tocilizumab	>0.9999
		Nsi Mock Vehicle vs. Pool ADT Tocilizumab	0.6028
		Nsi ADT Vehicle vs. Pool Mock Vehicle	0.7935
		Nsi ADT Vehicle vs. Pool ADT Vehicle	0.0023
		Nsi ADT Vehicle vs. Nsi Mock Tocilizumab	>0.9999
		Nsi ADT Vehicle vs. Nsi ADT Tocilizumab	0.9934
		Nsi ADT Vehicle vs. Pool Mock Tocilizumab	0.9931
		Nsi ADT Vehicle vs. Pool ADT Tocilizumab	0.9054
		Pool Mock Vehicle vs. Pool ADT Vehicle	0.043
		Pool Mock Vehicle vs. Nsi Mock Tocilizumab	0.7696
		Pool Mock Vehicle vs. Nsi ADT Tocilizumab	0.3692
		Pool Mock Vehicle vs. Pool Mock Tocilizumab	0.9937
		Pool Mock Vehicle vs. Pool ADT Tocilizumab	0.1758
		Pool ADT Vehicle vs. Nsi Mock Tocilizumab	0.0021
		Pool ADT Vehicle vs. Nsi ADT Tocilizumab	0.0006
		Pool ADT Vehicle vs. Pool Mock Tocilizumab	0.0102
		Pool ADT Vehicle vs. Pool ADT Tocilizumab	0.0002
		Nsi Mock Tocilizumab vs. Nsi ADT Tocilizumab	0.9955
		Nsi Mock Tocilizumab vs. Pool Mock Tocilizumab	0.9903
		Nsi Mock Tocilizumab vs. Pool ADT Tocilizumab	0.9204

		Nsi ADT Tocilizumab vs. Pool Mock Tocilizumab	0.7904
		Nsi ADT Tocilizumab vs. Pool ADT Tocilizumab	0.9995
		Pool Mock Tocilizumab vs. Pool ADT Tocilizumab	0.5076
Figure 6A	ANOVA Tukey's Test	0	
		Nsi VC vs. Pool VC	>0.9999
		Nsi VC vs. Nsi SRB1KO	>0.9999
		Nsi VC vs. Pool SRB1 KO	>0.9999
		Nsi VC vs. Nsi 10 µM ITX5061	>0.9999
		Nsi VC vs. Pool 10 µM ITX5061	>0.9999
		Nsi VC vs. Nsi SRB1KO 10 µM ITX5061	>0.9999
		Nsi VC vs. Pool SRB1 KO 10 µM ITX5061	>0.9999
		Pool VC vs. Nsi SRB1KO	>0.9999
		Pool VC vs. Pool SRB1 KO	>0.9999
		Pool VC vs. Nsi 10 µM ITX5061	>0.9999
		Pool VC vs. Pool 10 µM ITX5061	>0.9999
		Pool VC vs. Nsi SRB1KO 10 µM ITX5061	>0.9999
		Pool VC vs. Pool SRB1 KO 10 µM ITX5061	>0.9999
		Nsi SRB1KO vs. Pool SRB1 KO	>0.9999
		Nsi SRB1KO vs. Nsi 10 µM ITX5061	>0.9999
		Nsi SRB1KO vs. Pool 10 µM ITX5061	>0.9999
		Nsi SRB1KO vs. Nsi SRB1KO 10 µM ITX5061	>0.9999
		Nsi SRB1KO vs. Pool SRB1 KO 10 µM ITX5061	>0.9999
		Pool SRB1 KO vs. Nsi 10 µM ITX5061	>0.9999
		Pool SRB1 KO vs. Pool 10 µM ITX5061	>0.9999
		Pool SRB1 KO vs. Nsi SRB1KO 10 µM ITX5061	>0.9999
		Pool SRB1 KO vs. Pool SRB1 KO 10 µM ITX5061	>0.9999
		Nsi 10 µM ITX5061 vs. Pool 10 µM ITX5061	>0.9999
		Nsi 10 µM ITX5061 vs. Nsi SRB1KO 10 µM ITX5061	>0.9999
		Nsi 10 µM ITX5061 vs. Pool SRB1 KO 10 µM ITX5061	>0.9999
		Pool 10 µM ITX5061 vs. Nsi SRB1KO 10 µM ITX5061	>0.9999
		Pool 10 µM ITX5061 vs. Pool SRB1 KO 10 µM ITX5061	>0.9999
		Nsi SRB1KO 10 µM ITX5061 vs. Pool SRB1 KO 10 µM ITX5061	>0.9999
		2	
		Nsi VC vs. Pool VC	<0.0001
		Nsi VC vs. Nsi SRB1KO	0.9993
		Nsi VC vs. Pool SRB1 KO	0.9999
		Nsi VC vs. Nsi 10 µM ITX5061	0.99
		Nsi VC vs. Pool 10 µM ITX5061	>0.9999

Nsi VC vs. Nsi SRB1KO 10 μM ITX5061	0.9963
Nsi VC vs. Pool SRB1 KO 10 μM ITX5061	0.9988
Pool VC vs. Nsi SRB1KO	<0.0001
Pool VC vs. Pool SRB1 KO	<0.0001
Pool VC vs. Nsi 10 μM ITX5061	<0.0001
Pool VC vs. Pool 10 μM ITX5061	<0.0001
Pool VC vs. Nsi SRB1KO 10 μM ITX5061	<0.0001
Pool VC vs. Pool SRB1 KO 10 μM ITX5061	<0.0001
Nsi SRB1KO vs. Pool SRB1 KO	>0.9999
Nsi SRB1KO vs. Nsi 10 μM ITX5061	0.8608
Nsi SRB1KO vs. Pool 10 μM ITX5061	0.9841
Nsi SRB1KO vs. Nsi SRB1KO 10 μM ITX5061	0.9107
Nsi SRB1KO vs. Pool SRB1 KO 10 μM ITX5061	0.9448
Pool SRB1 KO vs. Nsi 10 μM ITX5061	0.9067
Pool SRB1 KO vs. Pool 10 μM ITX5061	0.9929
Pool SRB1 KO vs. Nsi SRB1KO 10 μM ITX5061	0.9448
Pool SRB1 KO vs. Pool SRB1 KO 10 μM ITX5061	0.9687
Nsi 10 μM ITX5061 vs. Pool 10 μM ITX5061	0.9997
Nsi 10 μM ITX5061 vs. Nsi SRB1KO 10 μM ITX5061	>0.9999
Nsi 10 μM ITX5061 vs. Pool SRB1 KO 10 μM ITX5061	>0.9999
Pool 10 μM ITX5061 vs. Nsi SRB1KO 10 μM ITX5061	>0.9999
Pool 10 μM ITX5061 vs. Pool SRB1 KO 10 μM ITX5061	>0.9999
Nsi SRB1KO 10 μM ITX5061 vs. Pool SRB1 KO 10 μM ITX5061	>0.9999
4	
Nsi VC vs. Pool VC	<0.0001
Nsi VC vs. Nsi SRB1KO	0.8556
Nsi VC vs. Pool SRB1 KO	>0.9999
Nsi VC vs. Nsi 10 μM ITX5061	0.0248
Nsi VC vs. Pool 10 μM ITX5061	0.9841
Nsi VC vs. Nsi SRB1KO 10 μM ITX5061	0.0416
Nsi VC vs. Pool SRB1 KO 10 μM ITX5061	0.2467
Pool VC vs. Nsi SRB1KO	<0.0001
Pool VC vs. Pool SRB1 KO	<0.0001
Pool VC vs. Nsi 10 μM ITX5061	<0.0001
Pool VC vs. Pool 10 μM ITX5061	<0.0001
Pool VC vs. Nsi SRB1KO 10 μM ITX5061	<0.0001
Pool VC vs. Pool SRB1 KO 10 μM ITX5061	<0.0001
Nsi SRB1KO vs. Pool SRB1 KO	0.8556
Nsi SRB1KO vs. Nsi 10 μM ITX5061	0.4644
Nsi SRB1KO vs. Pool 10 μM ITX5061	0.9997

		Nsi SRB1KO vs. Nsi SRB1KO 10 µM ITX5061	0.5925
		Nsi SRB1KO vs. Pool SRB1 KO 10 µM ITX5061	0.9647
		Pool SRB1 KO vs. Nsi 10 µM ITX5061	0.0248
		Pool SRB1 KO vs. Pool 10 µM ITX5061	0.9841
		Pool SRB1 KO vs. Nsi SRB1KO 10 µM ITX5061	0.0416
		Pool SRB1 KO vs. Pool SRB1 KO 10 µM ITX5061	0.2467
		Nsi 10 µM ITX5061 vs. Pool 10 µM ITX5061	0.2071
		Nsi 10 µM ITX5061 vs. Nsi SRB1KO 10 µM ITX5061	>0.9999
		Nsi 10 µM ITX5061 vs. Pool SRB1 KO 10 µM ITX5061	0.9724
		Pool 10 µM ITX5061 vs. Nsi SRB1KO 10 µM ITX5061	0.2969
		Pool 10 µM ITX5061 vs. Pool SRB1 KO 10 µM ITX5061	0.7858
		Nsi SRB1KO 10 µM ITX5061 vs. Pool SRB1 KO 10 µM ITX5061	0.9922
Figure 6B	ANOVA Tukey's Test	FM	
		Nsi CTRL vs. Pool SPRY2 KD CTRL	0.9043
		Nsi CTRL vs. Nsi SRB1 KO	0.9806
		Nsi CTRL vs. Pool SPRY2 KD SRB1 KO	0.7746
		Pool SPRY2 KD CTRL vs. Nsi SRB1 KO	0.9906
		Pool SPRY2 KD CTRL vs. Pool SPRY2 KD SRB1 KO	0.3883
		Nsi SRB1 KO vs. Pool SPRY2 KD SRB1 KO	0.5533
		FM ITX5061	
		Nsi CTRL vs. Pool SPRY2 KD CTRL	0.9917
		Nsi CTRL vs. Nsi SRB1 KO	0.6755
		Nsi CTRL vs. Pool SPRY2 KD SRB1 KO	0.9865
		Pool SPRY2 KD CTRL vs. Nsi SRB1 KO	0.5079
		Pool SPRY2 KD CTRL vs. Pool SPRY2 KD SRB1 KO	>0.9999
		Nsi SRB1 KO vs. Pool SPRY2 KD SRB1 KO	0.4783
		ADT	
		Nsi CTRL vs. Pool SPRY2 KD CTRL	0.0065
		Nsi CTRL vs. Nsi SRB1 KO	<0.0001
		Nsi CTRL vs. Pool SPRY2 KD SRB1 KO	<0.0001
		Pool SPRY2 KD CTRL vs. Nsi SRB1 KO	<0.0001
		Pool SPRY2 KD CTRL vs. Pool SPRY2 KD SRB1 KO	<0.0001
		Nsi SRB1 KO vs. Pool SPRY2 KD SRB1 KO	0.8823
		ADT ITX5061	
		Nsi CTRL vs. Pool SPRY2 KD CTRL	0.9317
		Nsi CTRL vs. Nsi SRB1 KO	0.9834

		Nsi CTRL vs. Pool SPRY2 KD SRB1 KO	0.9761
		Pool SPRY2 KD CTRL vs. Nsi SRB1 KO	0.9952
		Pool SPRY2 KD CTRL vs. Pool SPRY2 KD SRB1 KO	0.9976
		Nsi SRB1 KO vs. Pool SPRY2 KD SRB1 KO	>0.9999
Figure 6C	ANOVA Tukey's Test	Nsi Mock Vehicle vs. Nsi ADT Vehicle	0.0039
		Nsi Mock Vehicle vs. Pool Mock Vehicle	>0.9999
		Nsi Mock Vehicle vs. Pool ADT Vehicle	>0.9999
		Nsi Mock Vehicle vs. Nsi Mock ITX5061	0.5485
		Nsi Mock Vehicle vs. Nsi ADT ITX5061	0.0009
		Nsi Mock Vehicle vs. Pool Mock ITX5061	0.9978
		Nsi Mock Vehicle vs. Pool ADT ITX5061	0.016
		Nsi ADT Vehicle vs. Pool Mock Vehicle	0.0036
		Nsi ADT Vehicle vs. Pool ADT Vehicle	0.0014
		Nsi ADT Vehicle vs. Nsi Mock ITX5061	0.3146
		Nsi ADT Vehicle vs. Nsi ADT ITX5061	0.9993
		Nsi ADT Vehicle vs. Pool Mock ITX5061	0.0007
		Nsi ADT Vehicle vs. Pool ADT ITX5061	0.9994
		Pool Mock Vehicle vs. Pool ADT Vehicle	>0.9999
		Pool Mock Vehicle vs. Nsi Mock ITX5061	0.5246
		Pool Mock Vehicle vs. Nsi ADT ITX5061	0.0008
		Pool Mock Vehicle vs. Pool Mock ITX5061	0.9985
		Pool Mock Vehicle vs. Pool ADT ITX5061	0.0146
		Pool ADT Vehicle vs. Nsi Mock ITX5061	0.3232
		Pool ADT Vehicle vs. Nsi ADT ITX5061	0.0003
		Pool ADT Vehicle vs. Pool Mock ITX5061	>0.9999
		Pool ADT Vehicle vs. Pool ADT ITX5061	0.0059
		Nsi Mock ITX5061 vs. Nsi ADT ITX5061	0.1163
		Nsi Mock ITX5061 vs. Pool Mock ITX5061	0.2046
		Nsi Mock ITX5061 vs. Pool ADT ITX5061	0.6331
		Nsi ADT ITX5061 vs. Pool Mock ITX5061	0.0001
		Nsi ADT ITX5061 vs. Pool ADT ITX5061	0.9586
		Pool Mock ITX5061 vs. Pool ADT ITX5061	0.0029
Figure 6D	ANOVA Tukey's Test	Nsi Mock Vehicle vs. Nsi ADT Vehicle	0.0802
		Nsi Mock Vehicle vs. Pool Mock Vehicle	0.0446
		Nsi Mock Vehicle vs. Pool ADT Vehicle	0.0327
		Nsi Mock Vehicle vs. Nsi Mock Simvastatin	0.9389
		Nsi Mock Vehicle vs. Nsi ADT Simvastatin	0.095
		Nsi Mock Vehicle vs. Pool Mock Simvastatin	0.6123
		Nsi Mock Vehicle vs. Pool ADT Simvastatin	0.0867
		Nsi Mock Vehicle vs. Nsi Mock ITX5061	>0.9999
		Nsi Mock Vehicle vs. Nsi ADT ITX5061	0.0798

Nsi Mock Vehicle vs. Pool Mock ITX5061	>0.9999
Nsi Mock Vehicle vs. Pool ADT ITX5061	0.0901
Nsi ADT Vehicle vs. Pool Mock Vehicle	<0.0001
Nsi ADT Vehicle vs. Pool ADT Vehicle	<0.0001
Nsi ADT Vehicle vs. Nsi Mock Simvastatin	0.7336
Nsi ADT Vehicle vs. Nsi ADT Simvastatin	>0.9999
Nsi ADT Vehicle vs. Pool Mock Simvastatin	0.9772
Nsi ADT Vehicle vs. Pool ADT Simvastatin	>0.9999
Nsi ADT Vehicle vs. Nsi Mock ITX5061	0.248
Nsi ADT Vehicle vs. Nsi ADT ITX5061	>0.9999
Nsi ADT Vehicle vs. Pool Mock ITX5061	0.2212
Nsi ADT Vehicle vs. Pool ADT ITX5061	>0.9999
Pool Mock Vehicle vs. Pool ADT Vehicle	>0.9999
Pool Mock Vehicle vs. Nsi Mock Simvastatin	0.0015
Pool Mock Vehicle vs. Nsi ADT Simvastatin	<0.0001
Pool Mock Vehicle vs. Pool Mock Simvastatin	0.0003
Pool Mock Vehicle vs. Pool ADT Simvastatin	<0.0001
Pool Mock Vehicle vs. Nsi Mock ITX5061	0.0115
Pool Mock Vehicle vs. Nsi ADT ITX5061	<0.0001
Pool Mock Vehicle vs. Pool Mock ITX5061	0.0134
Pool Mock Vehicle vs. Pool ADT ITX5061	<0.0001
Pool ADT Vehicle vs. Nsi Mock Simvastatin	0.0011
Pool ADT Vehicle vs. Nsi ADT Simvastatin	<0.0001
Pool ADT Vehicle vs. Pool Mock Simvastatin	0.0002
Pool ADT Vehicle vs. Pool ADT Simvastatin	<0.0001
Pool ADT Vehicle vs. Nsi Mock ITX5061	0.0082
Pool ADT Vehicle vs. Nsi ADT ITX5061	<0.0001
Pool ADT Vehicle vs. Pool Mock ITX5061	0.0097
Pool ADT Vehicle vs. Pool ADT ITX5061	<0.0001
Nsi Mock Simvastatin vs. Nsi ADT Simvastatin	0.7804
Nsi Mock Simvastatin vs. Pool Mock Simvastatin	>0.9999
Nsi Mock Simvastatin vs. Pool ADT Simvastatin	0.7555
Nsi Mock Simvastatin vs. Nsi Mock ITX5061	0.999
Nsi Mock Simvastatin vs. Nsi ADT ITX5061	0.7322
Nsi Mock Simvastatin vs. Pool Mock ITX5061	0.9981
Nsi Mock Simvastatin vs. Pool ADT ITX5061	0.766
Nsi ADT Simvastatin vs. Pool Mock Simvastatin	0.9863
Nsi ADT Simvastatin vs. Pool ADT Simvastatin	>0.9999
Nsi ADT Simvastatin vs. Nsi Mock ITX5061	0.2843
Nsi ADT Simvastatin vs. Nsi ADT ITX5061	>0.9999
Nsi ADT Simvastatin vs. Pool Mock ITX5061	0.2548
Nsi ADT Simvastatin vs. Pool ADT ITX5061	>0.9999

		Pool Mock Simvastatin vs. Pool ADT Simvastatin	0.9818
		Pool Mock Simvastatin vs. Nsi Mock ITX5061	0.9189
		Pool Mock Simvastatin vs. Nsi ADT ITX5061	0.9769
		Pool Mock Simvastatin vs. Pool Mock ITX5061	0.8959
		Pool Mock Simvastatin vs. Pool ADT ITX5061	0.9838
		Pool ADT Simvastatin vs. Nsi Mock ITX5061	0.2642
		Pool ADT Simvastatin vs. Nsi ADT ITX5061	>0.9999
		Pool ADT Simvastatin vs. Pool Mock ITX5061	0.2362
		Pool ADT Simvastatin vs. Pool ADT ITX5061	>0.9999
		Nsi Mock ITX5061 vs. Nsi ADT ITX5061	0.247
		Nsi Mock ITX5061 vs. Pool Mock ITX5061	>0.9999
		Nsi Mock ITX5061 vs. Pool ADT ITX5061	0.2725
		Nsi ADT ITX5061 vs. Pool Mock ITX5061	0.2203
		Nsi ADT ITX5061 vs. Pool ADT ITX5061	>0.9999
		Pool Mock ITX5061 vs. Pool ADT ITX5061	0.2438
Figure 6E	Unpaired t test Two-tailed	Nsi ADT Vehicle vs Nsi Mock Vehicle (Testosterone)	0.0075
		Nsi ADT Simvastatin vs Nsi Mock Simvastatin (Testosterone)	<0.0001
		Nsi ADT ITX5061 vs Nsi Mock ITX5061 (Testosterone)	0.0322
Figure 6E	ANOVA Tukey's Test	Nsi Mock Vehicle vs. Nsi ADT Vehicle	0.931
		Nsi Mock Vehicle vs. Pool Mock Vehicle	>0.9999
		Nsi Mock Vehicle vs. Pool ADT Vehicle	0.0303
		Nsi Mock Vehicle vs. Nsi Mock Simvastatin	>0.9999
		Nsi Mock Vehicle vs. Nsi ADT Simvastatin	0.9389
		Nsi Mock Vehicle vs. Pool Mock Simvastatin	>0.9999
		Nsi Mock Vehicle vs. Pool ADT Simvastatin	0.9371
		Nsi Mock Vehicle vs. Nsi Mock ITX5061	>0.9999
		Nsi Mock Vehicle vs. Nsi ADT ITX5061	0.9272
		Nsi Mock Vehicle vs. Pool Mock ITX5061	0.9481
		Nsi Mock Vehicle vs. Pool ADT ITX5061	0.9404
		Nsi ADT Vehicle vs. Pool Mock Vehicle	0.9012
		Nsi ADT Vehicle vs. Pool ADT Vehicle	0.0009
		Nsi ADT Vehicle vs. Nsi Mock Simvastatin	0.9974
		Nsi ADT Vehicle vs. Nsi ADT Simvastatin	>0.9999
		Nsi ADT Vehicle vs. Pool Mock Simvastatin	0.9905
		Nsi ADT Vehicle vs. Pool ADT Simvastatin	>0.9999
		Nsi ADT Vehicle vs. Nsi Mock ITX5061	0.9872
		Nsi ADT Vehicle vs. Nsi ADT ITX5061	>0.9999
		Nsi ADT Vehicle vs. Pool Mock ITX5061	0.2011
		Nsi ADT Vehicle vs. Pool ADT ITX5061	>0.9999

Pool Mock Vehicle vs. Pool ADT Vehicle	0.0372
Pool Mock Vehicle vs. Nsi Mock Simvastatin	>0.9999
Pool Mock Vehicle vs. Nsi ADT Simvastatin	0.9113
Pool Mock Vehicle vs. Pool Mock Simvastatin	>0.9999
Pool Mock Vehicle vs. Pool ADT Simvastatin	0.9089
Pool Mock Vehicle vs. Nsi Mock ITX5061	>0.9999
Pool Mock Vehicle vs. Nsi ADT ITX5061	0.8966
Pool Mock Vehicle vs. Pool Mock ITX5061	0.9669
Pool Mock Vehicle vs. Pool ADT ITX5061	0.9131
Pool ADT Vehicle vs. Nsi Mock Simvastatin	0.009
Pool ADT Vehicle vs. Nsi ADT Simvastatin	0.001
Pool ADT Vehicle vs. Pool Mock Simvastatin	0.0132
Pool ADT Vehicle vs. Pool ADT Simvastatin	0.001
Pool ADT Vehicle vs. Nsi Mock ITX5061	0.0146
Pool ADT Vehicle vs. Nsi ADT ITX5061	0.0009
Pool ADT Vehicle vs. Pool Mock ITX5061	0.4328
Pool ADT Vehicle vs. Pool ADT ITX5061	0.001
Nsi Mock Simvastatin vs. Nsi ADT Simvastatin	0.998
Nsi Mock Simvastatin vs. Pool Mock Simvastatin	>0.9999
Nsi Mock Simvastatin vs. Pool ADT Simvastatin	0.9979
Nsi Mock Simvastatin vs. Nsi Mock ITX5061	>0.9999
Nsi Mock Simvastatin vs. Nsi ADT ITX5061	0.9971
Nsi Mock Simvastatin vs. Pool Mock ITX5061	0.7212
Nsi Mock Simvastatin vs. Pool ADT ITX5061	0.9981
Nsi ADT Simvastatin vs. Pool Mock Simvastatin	0.9923
Nsi ADT Simvastatin vs. Pool ADT Simvastatin	>0.9999
Nsi ADT Simvastatin vs. Nsi Mock ITX5061	0.9894
Nsi ADT Simvastatin vs. Nsi ADT ITX5061	>0.9999
Nsi ADT Simvastatin vs. Pool Mock ITX5061	0.2117
Nsi ADT Simvastatin vs. Pool ADT ITX5061	>0.9999
Pool Mock Simvastatin vs. Pool ADT Simvastatin	0.9919
Pool Mock Simvastatin vs. Nsi Mock ITX5061	>0.9999
Pool Mock Simvastatin vs. Nsi ADT ITX5061	0.9896
Pool Mock Simvastatin vs. Pool Mock ITX5061	0.8113
Pool Mock Simvastatin vs. Pool ADT ITX5061	0.9926
Pool ADT Simvastatin vs. Nsi Mock ITX5061	0.9889
Pool ADT Simvastatin vs. Nsi ADT ITX5061	>0.9999
Pool ADT Simvastatin vs. Pool Mock ITX5061	0.2091
Pool ADT Simvastatin vs. Pool ADT ITX5061	>0.9999
Nsi Mock ITX5061 vs. Nsi ADT ITX5061	0.9861
Nsi Mock ITX5061 vs. Pool Mock ITX5061	0.833
Nsi Mock ITX5061 vs. Pool ADT ITX5061	0.9898

		Nsi ADT ITX5061 vs. Pool Mock ITX5061	0.1966
		Nsi ADT ITX5061 vs. Pool ADT ITX5061	>0.9999
		Pool Mock ITX5061 vs. Pool ADT ITX5061	0.2137
Figure 6F	ANOVA Tukey's Test	Mock vs. ADT Vehicle	<0.0001
		Mock vs. ADT ITX5061	<0.0001
		ADT Vehicle vs. ADT ITX5061	<0.0001
Figure EV1G	ANOVA Sidak's test	Mock Vs ADT :-	
		Nuclear Ki67	0.815
		Membrane p-AKT	0.6301
		p-ERK1/2	0.2867
		Nuclear AR	0.0008
Figure EV1H	ANOVA Sidak's test	Mock vs ADT:-	
		WT	0.0014
		Ptenfl/+	<0.0001
		Spry2fl/+	0.025
Figure EV1J	ANOVA Tukey's Test	Membrane p-AKT	
		WT Mock vs. WT ADT	0.9972
		Ptenfl/+ Mock vs. Ptenfl/+ ADT	0.0011
		Spry2fl/+ Mock vs. Spry2fl/+ ADT	0.0537
		p-ERK1/2	
		WT Mock vs. WT ADT	0.0274
		Ptenfl/+ Mock vs. Ptenfl/+ ADT	0.0404
		Spry2fl/+ Mock vs. Spry2fl/+ ADT	0.1318
		Nuclear AR	
		WT Mock vs. WT ADT	<0.0001
		Ptenfl/+ Mock vs. Ptenfl/+ ADT	<0.0001
		Spry2fl/+ Mock vs. Spry2fl/+ ADT	<0.0001
Figure EV2B	ANOVA Sidak's test	AR	
		LNCaP vs. LNCaP AI	>0.9999
		LNCaP vs. CWR22Res	0.8356
		LNCaP vs. CWR22RV1	0.9581
		LNCaP vs. VCaP	<0.0001
		AR V7	
		LNCaP vs. LNCaP AI	>0.9999
		LNCaP vs. CWR22Res	<0.0001
		LNCaP vs. CWR22RV1	<0.0001
		LNCaP vs. VCaP	<0.0001

Figure EV2C	ANOVA Sidak's test	CWR22Res FBS - CWR22Res CSS	
		0	>0.9999
		2	<0.0001
		4	<0.0001
		CWR22RV1 FBS - CWR22RV1 CSS	
		0	>0.9999
		2	0.8839
		4	0.759
Figure EV2D	ANOVA Dunnett's test	CWR Nsi vs. CWR CL3	0.0001
		CWR Nsi vs. CWR Pool	0.0001
		CWR22Res Mock - CWR22Res ADT	
Figure EV2E	ANOVA Sidak's test	0	>0.9999
		30	0.9948
		60	<0.0001
Figure EV2F	Log-rank (Mantel-Cox) test	Comparison between ADT treated mice	0.0004
	Log-rank (Mantel-Cox) test	Comparison between Mock Vs ADT(Nsi)	0.0007
	Log-rank (Mantel-Cox) test	Comparison between Mock Vs ADT(Cl3)	0.0044
	Log-rank (Mantel-Cox) test	Comparison between Mock Vs ADT(Pool)	0.0064
Figure EV2I	ANOVA Tukey's Test	Nsi Mock vs. CL3 Mock	0.8564
		Nsi Mock vs. Pool Mock	0.9935
		Nsi Mock vs. Nsi ADT	0.0893
		Nsi Mock vs. CL3 ADT	0.0005
		Nsi Mock vs. Pool ADT	<0.0001
		CL3 Mock vs. Pool Mock	0.9899
		CL3 Mock vs. Nsi ADT	0.0068
		CL3 Mock vs. CL3 ADT	0.0077
		CL3 Mock vs. Pool ADT	0.0005
		Pool Mock vs. Nsi ADT	0.0276
		Pool Mock vs. CL3 ADT	0.0018
		Pool Mock vs. Pool ADT	0.0001
		Nsi ADT vs. CL3 ADT	<0.0001
		Nsi ADT vs. Pool ADT	<0.0001
		CL3 ADT vs. Pool ADT	0.8682
Figure EV2O	ANOVA Tukey's Test	Nsi siCTRL vs. Pool siCTRL	0.0013
		Nsi siCTRL vs. Nsi siPTEN	0.0475
		Nsi siCTRL vs. Pool siPTEN	0.0004
		Pool siCTRL vs. Nsi siPTEN	0.0821
		Pool siCTRL vs. Pool siPTEN	0.6384

		Nsi siPTEN vs. Pool siPTEN	0.0154
Figure EV3B	ANOVA Sidak's test	Nuclear AR	
		Nsi Mock vs. Nsi ADT	<0.0001
		Nsi Mock vs. CL3 Mock	>0.9999
		Nsi Mock vs. CL3 ADT	0.0591
		Nsi Mock vs. Pool Mock	0.9994
		Nsi Mock vs. Pool ADT	0.174
		Nsi ADT vs. CL3 Mock	<0.0001
		Nsi ADT vs. CL3 ADT	<0.0001
		Nsi ADT vs. Pool Mock	<0.0001
		Nsi ADT vs. Pool ADT	<0.0001
		CL3 Mock vs. CL3 ADT	0.0139
		CL3 Mock vs. Pool Mock	>0.9999
		CL3 Mock vs. Pool ADT	0.0453
		CL3 ADT vs. Pool Mock	0.0069
		CL3 ADT vs. Pool ADT	>0.9999
		Pool Mock vs. Pool ADT	0.0229
		Cleaved Caspase 3	
		Nsi Mock vs. Nsi ADT	0.0298
		Nsi Mock vs. CL3 Mock	0.4154
		Nsi Mock vs. CL3 ADT	0.0754
		Nsi Mock vs. Pool Mock	0.3632
		Nsi Mock vs. Pool ADT	0.2433
		Nsi ADT vs. CL3 Mock	0.0001
		Nsi ADT vs. CL3 ADT	<0.0001
		Nsi ADT vs. Pool Mock	<0.0001
		Nsi ADT vs. Pool ADT	<0.0001
		CL3 Mock vs. CL3 ADT	0.9996
		CL3 Mock vs. Pool Mock	>0.9999
		CL3 Mock vs. Pool ADT	>0.9999
		CL3 ADT vs. Pool Mock	0.9999
		CL3 ADT vs. Pool ADT	>0.9999
		Pool Mock vs. Pool ADT	>0.9999
Figure EV3C	Unpaired t test Two-tailed	CL3 vs Nsi (HSD3B1)	0.0001
		Pool vs Nsi (HSD3B1)	0.0306
		Pool vs Nsi (CYP17A1)	0.0078
Figure EV3I	ANOVA Tukey's Test	0 FBS	
		CTRL VC vs. CTRL 10μM Abiraterone	>0.9999
		CTRL VC vs. CTRL 20 μM Abiraterone	>0.9999
		CTRL VC vs. SPRY2 VC	>0.9999

CTRL VC vs. SPRY2 10 μM Abiraterone	>0.9999
CTRL VC vs. SPRY2 20 μM Abiraterone	>0.9999
CTRL 10 μM Abiraterone vs. CTRL 20 μM Abiraterone	>0.9999
CTRL 10 μM Abiraterone vs. SPRY2 VC	>0.9999
CTRL 10 μM Abiraterone vs. SPRY2 μM Abiraterone	>0.9999
CTRL 10 μM Abiraterone vs. SPRY2 20 μM Abiraterone	>0.9999
CTRL 20 μM Abiraterone vs. SPRY2 VC	>0.9999
CTRL 20 μM Abiraterone vs. SPRY2 10 μM Abiraterone	>0.9999
CTRL 20 μM Abiraterone vs. SPRY2 20 μM Abiraterone	>0.9999
SPRY2 VC vs. SPRY2 10 μM Abiraterone	>0.9999
SPRY2 VC vs. SPRY2 20 μM Abiraterone	>0.9999
SPRY2 10 μM Abiraterone vs. SPRY2 20 μM Abiraterone	>0.9999
2 FBS	
CTRL VC vs. CTRL 10μM Abiraterone	<0.0001
CTRL VC vs. CTRL 20 μM Abiraterone	<0.0001
CTRL VC vs. SPRY2 VC	0.0004
CTRL VC vs. SPRY2 10 μM Abiraterone	<0.0001
CTRL VC vs. SPRY2 20 μM Abiraterone	<0.0001
CTRL 10 μM Abiraterone vs. CTRL 20 μM Abiraterone	0.4881
CTRL 10 μM Abiraterone vs. SPRY2 VC	0.0001
CTRL 10 μM Abiraterone vs. SPRY2 μM Abiraterone	0.9858
CTRL 10 μM Abiraterone vs. SPRY2 20 μM Abiraterone	0.4973
CTRL 20 μM Abiraterone vs. SPRY2 VC	<0.0001
CTRL 20 μM Abiraterone vs. SPRY2 10 μM Abiraterone	0.8656
CTRL 20 μM Abiraterone vs. SPRY2 20 μM Abiraterone	>0.9999
SPRY2 VC vs. SPRY2 10 μM Abiraterone	<0.0001
SPRY2 VC vs. SPRY2 20 μM Abiraterone	<0.0001
SPRY2 10 μM Abiraterone vs. SPRY2 20 μM Abiraterone	0.8719
4 FBS	
CTRL VC vs. CTRL 10μM Abiraterone	<0.0001
CTRL VC vs. CTRL 20 μM Abiraterone	<0.0001
CTRL VC vs. SPRY2 VC	<0.0001
CTRL VC vs. SPRY2 10 μM Abiraterone	<0.0001
CTRL VC vs. SPRY2 20 μM Abiraterone	<0.0001
CTRL 10 μM Abiraterone vs. CTRL 20 μM Abiraterone	0.0003
CTRL 10 μM Abiraterone vs. SPRY2 VC	<0.0001
CTRL 10 μM Abiraterone vs. SPRY2 μM Abiraterone	0.7453
CTRL 10 μM Abiraterone vs. SPRY2 20 μM Abiraterone	0.0409
CTRL 20 μM Abiraterone vs. SPRY2 VC	<0.0001

CTRL 20 µM Abiraterone vs. SPRY2 10 µM Abiraterone	<0.0001
CTRL 20 µM Abiraterone vs. SPRY2 20 µM Abiraterone	0.4699
SPRY2 VC vs. SPRY2 10 µM Abiraterone	<0.0001
SPRY2 VC vs. SPRY2 20 µM Abiraterone	<0.0001
SPRY2 10 µM Abiraterone vs. SPRY2 20 µM Abiraterone	0.001
0 CSS	
CTRL VC vs. CTRL 10µM Abiraterone	>0.9999
CTRL VC vs. CTRL 20 µM Abiraterone	>0.9999
CTRL VC vs. SPRY2 VC	>0.9999
CTRL VC vs. SPRY2 10 µM Abiraterone	>0.9999
CTRL VC vs. SPRY2 20 µM Abiraterone	>0.9999
CTRL 10 µM Abiraterone vs. CTRL 20 µM Abiraterone	>0.9999
CTRL 10 µM Abiraterone vs. SPRY2 VC	>0.9999
CTRL 10 µM Abiraterone vs. SPRY2 µM Abiraterone	>0.9999
CTRL 10 µM Abiraterone vs. SPRY2 20 µM Abiraterone	>0.9999
CTRL 20 µM Abiraterone vs. SPRY2 VC	>0.9999
CTRL 20 µM Abiraterone vs. SPRY2 10 µM Abiraterone	>0.9999
CTRL 20 µM Abiraterone vs. SPRY2 20 µM Abiraterone	>0.9999
SPRY2 VC vs. SPRY2 10 µM Abiraterone	>0.9999
SPRY2 VC vs. SPRY2 20 µM Abiraterone	>0.9999
SPRY2 10 µM Abiraterone vs. SPRY2 20 µM Abiraterone	>0.9999
2 CSS	
CTRL VC vs. CTRL 10µM Abiraterone	<0.0001
CTRL VC vs. CTRL 20 µM Abiraterone	0.2233
CTRL VC vs. SPRY2 VC	0.282
CTRL VC vs. SPRY2 10 µM Abiraterone	0.2878
CTRL VC vs. SPRY2 20 µM Abiraterone	0.9389
CTRL 10 µM Abiraterone vs. CTRL 20 µM Abiraterone	<0.0001
CTRL 10 µM Abiraterone vs. SPRY2 VC	0.0037
CTRL 10 µM Abiraterone vs. SPRY2 µM Abiraterone	<0.0001
CTRL 10 µM Abiraterone vs. SPRY2 20 µM Abiraterone	<0.0001
CTRL 20 µM Abiraterone vs. SPRY2 VC	0.001
CTRL 20 µM Abiraterone vs. SPRY2 10 µM Abiraterone	>0.9999
CTRL 20 µM Abiraterone vs. SPRY2 20 µM Abiraterone	0.0306
SPRY2 VC vs. SPRY2 10 µM Abiraterone	0.0016
SPRY2 VC vs. SPRY2 20 µM Abiraterone	0.8151
SPRY2 10 µM Abiraterone vs. SPRY2 20 µM Abiraterone	0.0437

		4 CSS	
		CTRL VC vs. CTRL 10µM Abiraterone	<0.0001
		CTRL VC vs. CTRL 20 µM Abiraterone	<0.0001
		CTRL VC vs. SPRY2 VC	0.6308
		CTRL VC vs. SPRY2 10 µM Abiraterone	<0.0001
		CTRL VC vs. SPRY2 20 µM Abiraterone	0.1299
		CTRL 10 µM Abiraterone vs. CTRL 20 µM Abiraterone	<0.0001
		CTRL 10 µM Abiraterone vs. SPRY2 VC	<0.0001
		CTRL 10 µM Abiraterone vs. SPRY2 µM Abiraterone	<0.0001
		CTRL 10 µM Abiraterone vs. SPRY2 20 µM Abiraterone	<0.0001
		CTRL 20 µM Abiraterone vs. SPRY2 VC	<0.0001
		CTRL 20 µM Abiraterone vs. SPRY2 10 µM Abiraterone	0.8151
		CTRL 20 µM Abiraterone vs. SPRY2 20 µM Abiraterone	<0.0001
		SPRY2 VC vs. SPRY2 10 µM Abiraterone	<0.0001
		SPRY2 VC vs. SPRY2 20 µM Abiraterone	0.0026
		SPRY2 10 µM Abiraterone vs. SPRY2 20 µM Abiraterone	<0.0001
Figure EV3J	ANOVA Tukey's Test	FBS 0	
		Nsi VC vs. Pool SPRY2 KD VC	>0.9999
		Nsi VC vs. Nsi 10 µM Abiraterone	>0.9999
		Nsi VC vs. Pool SPRY2 KD 10 µM Abiraterone	>0.9999
		Nsi VC vs. Nsi 20 µM Abiraterone	>0.9999
		Nsi VC vs. Pool SPRY2 KD 20 µM Abiraterone	>0.9999
		Pool SPRY2 KD VC vs. Nsi 10 µM Abiraterone	>0.9999
		Pool SPRY2 KD VC vs. Pool SPRY2 KD 10 µM Abiraterone	>0.9999
		Pool SPRY2 KD VC vs. Nsi 20 µM Abiraterone	>0.9999
		Pool SPRY2 KD VC vs. Pool SPRY2 KD 20 µM Abiraterone	>0.9999
		Nsi 10 µM Abiraterone vs. Pool SPRY2 KD 10 µM Abiraterone	>0.9999
		Nsi 10 µM Abiraterone vs. Nsi 20 µM Abiraterone	>0.9999
		Nsi 10 µM Abiraterone vs. Pool SPRY2 KD 20 µM Abiraterone	>0.9999
		Pool SPRY2 KD 10 µM Abiraterone vs. Nsi 20 µM Abiraterone	>0.9999
		Pool SPRY2 KD 10 µM Abiraterone vs. Pool SPRY2 KD 20 µM Abiraterone	>0.9999
		Nsi 20 µM Abiraterone vs. Pool SPRY2 KD 20 µM Abiraterone	>0.9999
		FBS 2	
		Nsi VC vs. Pool SPRY2 KD VC	0.7776
		Nsi VC vs. Nsi 10 µM Abiraterone	<0.0001
		Nsi VC vs. Pool SPRY2 KD 10 µM Abiraterone	<0.0001
		Nsi VC vs. Nsi 20 µM Abiraterone	<0.0001
		Nsi VC vs. Pool SPRY2 KD 20 µM Abiraterone	<0.0001
		Pool SPRY2 KD VC vs. Nsi 10 µM Abiraterone	<0.0001

Pool SPRY2 KD VC vs. Pool SPRY2 KD 10 µM Abiraterone	<0.0001
Pool SPRY2 KD VC vs. Nsi 20 µM Abiraterone	<0.0001
Pool SPRY2 KD VC vs. Pool SPRY2 KD 20 µM Abiraterone	<0.0001
Nsi 10 µM Abiraterone vs. Pool SPRY2 KD 10 µM Abiraterone	<0.0001
Nsi 10 µM Abiraterone vs. Nsi 20 µM Abiraterone	<0.0001
Nsi 10 µM Abiraterone vs. Pool SPRY2 KD 20 µM Abiraterone	<0.0001
Pool SPRY2 KD 10 µM Abiraterone vs. Nsi 20 µM Abiraterone	0.9983
Pool SPRY2 KD 10 µM Abiraterone vs. Pool SPRY2 KD 20 µM Abiraterone	0.9966
Nsi 20 µM Abiraterone vs. Pool SPRY2 KD 20 µM Abiraterone	>0.9999
FBS 4	
Nsi VC vs. Pool SPRY2 KD VC	0.0112
Nsi VC vs. Nsi 10 µM Abiraterone	<0.0001
Nsi VC vs. Pool SPRY2 KD 10 µM Abiraterone	<0.0001
Nsi VC vs. Nsi 20 µM Abiraterone	<0.0001
Nsi VC vs. Pool SPRY2 KD 20 µM Abiraterone	<0.0001
Pool SPRY2 KD VC vs. Nsi 10 µM Abiraterone	<0.0001
Pool SPRY2 KD VC vs. Pool SPRY2 KD 10 µM Abiraterone	<0.0001
Pool SPRY2 KD VC vs. Nsi 20 µM Abiraterone	<0.0001
Pool SPRY2 KD VC vs. Pool SPRY2 KD 20 µM Abiraterone	<0.0001
Nsi 10 µM Abiraterone vs. Pool SPRY2 KD 10 µM Abiraterone	<0.0001
Nsi 10 µM Abiraterone vs. Nsi 20 µM Abiraterone	<0.0001
Nsi 10 µM Abiraterone vs. Pool SPRY2 KD 20 µM Abiraterone	<0.0001
Pool SPRY2 KD 10 µM Abiraterone vs. Nsi 20 µM Abiraterone	0.0074
Pool SPRY2 KD 10 µM Abiraterone vs. Pool SPRY2 KD 20 µM Abiraterone	0.0025
Nsi 20 µM Abiraterone vs. Pool SPRY2 KD 20 µM Abiraterone	0.9987
CSS 0	
Nsi VC vs. Pool SPRY2 KD VC	>0.9999
Nsi VC vs. Nsi 10 µM Abiraterone	>0.9999
Nsi VC vs. Pool SPRY2 KD 10 µM Abiraterone	>0.9999
Nsi VC vs. Nsi 20 µM Abiraterone	>0.9999
Nsi VC vs. Pool SPRY2 KD 20 µM Abiraterone	>0.9999
Pool SPRY2 KD VC vs. Nsi 10 µM Abiraterone	>0.9999
Pool SPRY2 KD VC vs. Pool SPRY2 KD 10 µM Abiraterone	>0.9999
Pool SPRY2 KD VC vs. Nsi 20 µM Abiraterone	>0.9999
Pool SPRY2 KD VC vs. Pool SPRY2 KD 20 µM Abiraterone	>0.9999
Nsi 10 µM Abiraterone vs. Pool SPRY2 KD 10 µM Abiraterone	>0.9999
Nsi 10 µM Abiraterone vs. Nsi 20 µM Abiraterone	>0.9999
Nsi 10 µM Abiraterone vs. Pool SPRY2 KD 20 µM	>0.9999

Abiraterone	
Pool SPRY2 KD 10 µM Abiraterone vs. Nsi 20 µM Abiraterone	>0.9999
Pool SPRY2 KD 10 µM Abiraterone vs. Pool SPRY2 KD 20 µM Abiraterone	>0.9999
Nsi 20 µM Abiraterone vs. Pool SPRY2 KD 20 µM Abiraterone	>0.9999
CSS 2	
Nsi VC vs. Pool SPRY2 KD VC	<0.0001
Nsi VC vs. Nsi 10 µM Abiraterone	0.2233
Nsi VC vs. Pool SPRY2 KD 10 µM Abiraterone	0.282
Nsi VC vs. Nsi 20 µM Abiraterone	0.2878
Nsi VC vs. Pool SPRY2 KD 20 µM Abiraterone	0.9389
Pool SPRY2 KD VC vs. Nsi 10 µM Abiraterone	<0.0001
Pool SPRY2 KD VC vs. Pool SPRY2 KD 10 µM Abiraterone	0.0037
Pool SPRY2 KD VC vs. Nsi 20 µM Abiraterone	<0.0001
Pool SPRY2 KD VC vs. Pool SPRY2 KD 20 µM Abiraterone	<0.0001
Nsi 10 µM Abiraterone vs. Pool SPRY2 KD 10 µM Abiraterone	0.001
Nsi 10 µM Abiraterone vs. Nsi 20 µM Abiraterone	>0.9999
Nsi 10 µM Abiraterone vs. Pool SPRY2 KD 20 µM Abiraterone	0.0306
Pool SPRY2 KD 10 µM Abiraterone vs. Nsi 20 µM Abiraterone	0.0016
Pool SPRY2 KD 10 µM Abiraterone vs. Pool SPRY2 KD 20 µM Abiraterone	0.8151
Nsi 20 µM Abiraterone vs. Pool SPRY2 KD 20 µM Abiraterone	0.0437
CSS 4	
Nsi VC vs. Pool SPRY2 KD VC	<0.0001
Nsi VC vs. Nsi 10 µM Abiraterone	<0.0001
Nsi VC vs. Pool SPRY2 KD 10 µM Abiraterone	0.6308
Nsi VC vs. Nsi 20 µM Abiraterone	<0.0001
Nsi VC vs. Pool SPRY2 KD 20 µM Abiraterone	0.1299
Pool SPRY2 KD VC vs. Nsi 10 µM Abiraterone	<0.0001
Pool SPRY2 KD VC vs. Pool SPRY2 KD 10 µM Abiraterone	<0.0001
Pool SPRY2 KD VC vs. Nsi 20 µM Abiraterone	<0.0001
Pool SPRY2 KD VC vs. Pool SPRY2 KD 20 µM Abiraterone	<0.0001
Nsi 10 µM Abiraterone vs. Pool SPRY2 KD 10 µM Abiraterone	<0.0001
Nsi 10 µM Abiraterone vs. Nsi 20 µM Abiraterone	0.8151
Nsi 10 µM Abiraterone vs. Pool SPRY2 KD 20 µM Abiraterone	<0.0001
Pool SPRY2 KD 10 µM Abiraterone vs. Nsi 20 µM Abiraterone	<0.0001
Pool SPRY2 KD 10 µM Abiraterone vs. Pool SPRY2 KD 20 µM Abiraterone	0.0026
Nsi 20 µM Abiraterone vs. Pool SPRY2 KD 20 µM Abiraterone	<0.0001

Figure EV3M	ANOVA Dunnett's test	CWR Nsi FBS vs. CWR Pool FBS	0.6704
		CWR Nsi FBS vs. CWR Nsi HSD3B1 KO FBS	0.9997
		CWR Nsi FBS vs. CWR Pool HSD3B1 KO FBS	0.9234
		CWR Nsi FBS vs. CWR Nsi CSS	0.0021
		CWR Nsi FBS vs. CWR Pool CSS	0.5651
		CWR Nsi FBS vs. CWR Nsi HSD3B1 KO CSS	0.0011
		CWR Nsi FBS vs. CWR Pool HSD3B1 KO CSS	0.001
Figure EV3O	Unpaired t test Two-tailed	Nsi shHER2 vs Nsi shSc	0.0064
		Pool shHER2 vs Pool shSc	0.0001
Figure EV4B	Unpaired t test Two-tailed	CTRL vs SPRY2	0.0498
Figure EV4F	ANOVA Tukey's test	LNCaP vs. CWR22Res	0.1678
		LNCaP vs. CWR22RV1	<0.0001
		LNCaP vs. DU145	0.0002
		CWR22Res vs. CWR22RV1	0.0002
		CWR22Res vs. DU145	0.0017
		CWR22RV1 vs. DU145	0.2321
Figure EV4G	Unpaired t test Two-tailed	IL6 Vs CTRL	0.0222
Figure EV4H	Unpaired t test Two-tailed	anti-IL6 vs ctrl IgG (hIL6)	0.014
		anti-IL6 vs ctrl IgG (HSD3B1)	0.0489
Figure EV4K	ANOVA Tukey's test	AR	
		Nsi Mock VC vs. Nsi ADT VC	<0.0001
		Nsi Mock VC vs. Pool Mock VC	0.987
		Nsi Mock VC vs. Pool ADT VC	0.0954
		Nsi Mock VC vs. Nsi Mock Tocilizumab	>0.9999
		Nsi Mock VC vs. Nsi ADT Tocilizumab	<0.0001
		Nsi Mock VC vs. Pool Mock Tocilizumab	0.2401
		Nsi Mock VC vs. Pool ADT Tocilizumab	<0.0001
		Nsi ADT VC vs. Pool Mock VC	<0.0001
		Nsi ADT VC vs. Pool ADT VC	<0.0001
		Nsi ADT VC vs. Nsi Mock Tocilizumab	<0.0001
		Nsi ADT VC vs. Nsi ADT Tocilizumab	0.9987
		Nsi ADT VC vs. Pool Mock Tocilizumab	<0.0001
		Nsi ADT VC vs. Pool ADT Tocilizumab	0.99
		Pool Mock VC vs. Pool ADT VC	0.012
		Pool Mock VC vs. Nsi Mock Tocilizumab	0.9746
		Pool Mock VC vs. Nsi ADT Tocilizumab	<0.0001
		Pool Mock VC vs. Pool Mock Tocilizumab	0.0388

		Pool Mock VC vs. Pool ADT Tocilizumab	<0.0001
		Pool ADT VC vs. Nsi Mock Tocilizumab	0.12
		Pool ADT VC vs. Nsi ADT Tocilizumab	<0.0001
		Pool ADT VC vs. Pool Mock Tocilizumab	0.9997
		Pool ADT VC vs. Pool ADT Tocilizumab	<0.0001
		Nsi Mock Tocilizumab vs. Nsi ADT Tocilizumab	<0.0001
		Nsi Mock Tocilizumab vs. Pool Mock Tocilizumab	0.2894
		Nsi Mock Tocilizumab vs. Pool ADT Tocilizumab	<0.0001
		Nsi ADT Tocilizumab vs. Pool Mock Tocilizumab	<0.0001
		Nsi ADT Tocilizumab vs. Pool ADT Tocilizumab	>0.9999
		Pool Mock Tocilizumab vs. Pool ADT Tocilizumab	<0.0001
		Cleaved caspase 3	
		Nsi Mock VC vs. Nsi ADT VC	0.0001
		Nsi Mock VC vs. Pool Mock VC	>0.9999
		Nsi Mock VC vs. Pool ADT VC	>0.9999
		Nsi Mock VC vs. Nsi Mock Tocilizumab	>0.9999
		Nsi Mock VC vs. Nsi ADT Tocilizumab	0.0002
		Nsi Mock VC vs. Pool Mock Tocilizumab	0.9894
		Nsi Mock VC vs. Pool ADT Tocilizumab	<0.0001
		Nsi ADT VC vs. Pool Mock VC	0.0003
		Nsi ADT VC vs. Pool ADT VC	0.0001
		Nsi ADT VC vs. Nsi Mock Tocilizumab	0.0001
		Nsi ADT VC vs. Nsi ADT Tocilizumab	>0.9999
		Nsi ADT VC vs. Pool Mock Tocilizumab	0.0013
		Nsi ADT VC vs. Pool ADT Tocilizumab	>0.9999
		Pool Mock VC vs. Pool ADT VC	>0.9999
		Pool Mock VC vs. Nsi Mock Tocilizumab	>0.9999
		Pool Mock VC vs. Nsi ADT Tocilizumab	0.0005
		Pool Mock VC vs. Pool Mock Tocilizumab	0.9994
		Pool Mock VC vs. Pool ADT Tocilizumab	0.0002
		Pool ADT VC vs. Nsi Mock Tocilizumab	>0.9999
		Pool ADT VC vs. Nsi ADT Tocilizumab	0.0002
		Pool ADT VC vs. Pool Mock Tocilizumab	0.9903
		Pool ADT VC vs. Pool ADT Tocilizumab	<0.0001
		Nsi Mock Tocilizumab vs. Nsi ADT Tocilizumab	0.0002
		Nsi Mock Tocilizumab vs. Pool Mock Tocilizumab	0.9931
		Nsi Mock Tocilizumab vs. Pool ADT Tocilizumab	0.0001
		Nsi ADT Tocilizumab vs. Pool Mock Tocilizumab	0.002
		Nsi ADT Tocilizumab vs. Pool ADT Tocilizumab	>0.9999
		Pool Mock Tocilizumab vs. Pool ADT Tocilizumab	0.001
Figure EV4M	Unpaired Two- tailed t test	AR	0.000144008
		Cleaved Caspase 3	0.004478318

		Ki67	0.004986585
Figure EV5G	Unpaired Two- tailed t test	15µM ITX5061 vs DMSO	0.0145
Figure EV5H	Unpaired Two- tailed t test	15µM ITX5061 vs DMSO	0.0419
Figure EV5I	Unpaired Two- tailed t test	15µM ITX5061 vs DMSO	0.0493
Figure EV5J	ANOVA Tukey's test	Nsi Ctrl Vs Nsi SRB1 KO (DMSO)	0.021327346
		Nsi Ctrl Vs Pool Ctrl (ITX-5061)	0.035819009
		Nsi Ctrl Vs Pool Ctrl (DMSO)	0.035819009
		Nsi Ctrl Vs Pool SRB1 KO (ITX-5061)	0.027102703
		Nsi Ctrl Vs Pool SRB1 KO (DMSO)	0.027102703
Figure EV5K	ANOVA Tukey's test	0	
		LNCaP-VC CTRL vs. LNCaP-SPRY2 VC	>0.9999
		LNCaP-VC CTRL vs. LNCaP-VC 10µM ITX5061	>0.9999
		LNCaP-VC CTRL vs. LNCaP-SPRY2 10 µM ITX5061	>0.9999
		LNCaP-VC CTRL vs. LNCaP-VC 15 µM ITX5061	>0.9999
		LNCaP-VC CTRL vs. LNCaP-SPRY2 15 µM ITX5061	>0.9999
		LNCaP-VC CTRL vs. LNCaP-VC 20 µM ITX5061	>0.9999
		LNCaP-VC CTRL vs. LNCaP-SPRY2 20 µM ITX5061	>0.9999
		LNCaP-SPRY2 VC vs. LNCaP-VC 10 µM ITX5061	>0.9999
		LNCaP-SPRY2 VC vs. LNCaP-SPRY2 10 µM ITX5061	>0.9999
		LNCaP-SPRY2 VC vs. LNCaP-VC 15 µM ITX5061	>0.9999
		LNCaP-SPRY2 VC vs. LNCaP-VC 20 µM ITX5061	>0.9999
		LNCaP-SPRY2 VC vs. LNCaP-SPRY2 20 µM ITX5061	>0.9999
		LNCaP-VC 10 µM ITX5061 vs. LNCaP-SPRY2 10 µM ITX5061	>0.9999
		LNCaP-VC 10 µM ITX5061 vs. LNCaP-VC 15 µM ITX5061	>0.9999
		LNCaP-VC 10 µM ITX5061 vs. LNCaP-SPRY2 15 µM ITX5061	>0.9999
		LNCaP-VC 10 µM ITX5061 vs. LNCaP-VC 20 µM ITX5061	>0.9999
		LNCaP-VC 10 µM ITX5061 vs. LNCaP-SPRY2 20 µM ITX5061	>0.9999
		LNCaP-SPRY2 10 µM ITX5061 vs. LNCaP-VC 15 µM ITX5061	>0.9999
		LNCaP-SPRY2 10 µM ITX5061 vs. LNCaP-SPRY2 15 µM ITX5061	>0.9999
		LNCaP-SPRY2 10 µM ITX5061 vs. LNCaP-VC 20 µM ITX5061	>0.9999
		LNCaP-SPRY2 10 µM ITX5061 vs. LNCaP-SPRY2 20 µM ITX5061	>0.9999
		LNCaP-VC 15 µM ITX5061 vs. LNCaP-SPRY2 15 µM ITX5061	>0.9999
		LNCaP-VC 15 µM ITX5061 vs. LNCaP-VC 20 µM ITX5061	>0.9999

LNCaP-VC 15 μM ITX5061 vs. LNCaP-SPRY2 20 μM ITX5061	>0.9999
LNCaP-SPRY2 15 μM ITX5061 vs. LNCaP-VC 20 μM ITX5061	>0.9999
LNCaP-SPRY2 15 μM ITX5061 vs. LNCaP-SPRY2 20 μM ITX5061	>0.9999
LNCaP-VC 20 μM ITX5061 vs. LNCaP-SPRY2 20 μM ITX5061	>0.9999

2

LNCaP-VC CTRL vs. LNCaP-SPRY2 VC	<0.0001
LNCaP-VC CTRL vs. LNCaP-VC 10μM ITX5061	<0.0001
LNCaP-VC CTRL vs. LNCaP-SPRY2 10 μM ITX5061	<0.0001
LNCaP-VC CTRL vs. LNCaP-VC 15 μM ITX5061	<0.0001
LNCaP-VC CTRL vs. LNCaP-SPRY2 15 μM ITX5061	<0.0001
LNCaP-VC CTRL vs. LNCaP-VC 20 μM ITX5061	<0.0001
LNCaP-VC CTRL vs. LNCaP-SPRY2 20 μM ITX5061	<0.0001
LNCaP-SPRY2 VC vs. LNCaP-VC 10 μM ITX5061	0.1117
LNCaP-SPRY2 VC vs. LNCaP-SPRY2 10 μM ITX5061	0.0011
LNCaP-SPRY2 VC vs. LNCaP-VC 15 μM ITX5061	0.0007
LNCaP-SPRY2 VC vs. LNCaP-VC 20 μM ITX5061	<0.0001
LNCaP-SPRY2 VC vs. LNCaP-SPRY2 20 μM ITX5061	<0.0001
LNCaP-VC 10 μM ITX5061 vs. LNCaP-SPRY2 10 μM ITX5061	<0.0001
LNCaP-VC 10 μM ITX5061 vs. LNCaP-VC 15 μM ITX5061	0.713
LNCaP-VC 10 μM ITX5061 vs. LNCaP-SPRY2 15 μM ITX5061	0.6137
LNCaP-VC 10 μM ITX5061 vs. LNCaP-VC 20 μM ITX5061	0.0192
LNCaP-VC 10 μM ITX5061 vs. LNCaP-SPRY2 20 μM ITX5061	0.2649
LNCaP-SPRY2 10 μM ITX5061 vs. LNCaP-VC 15 μM ITX5061	<0.0001
LNCaP-SPRY2 10 μM ITX5061 vs. LNCaP-SPRY2 15 μM ITX5061	>0.9999
LNCaP-SPRY2 10 μM ITX5061 vs. LNCaP-VC 20 μM ITX5061	0.5754
LNCaP-SPRY2 10 μM ITX5061 vs. LNCaP-SPRY2 20 μM ITX5061	0.9952
LNCaP-VC 15 μM ITX5061 vs. LNCaP-SPRY2 15 μM ITX5061	0.0117
LNCaP-VC 15 μM ITX5061 vs. LNCaP-VC 20 μM ITX5061	0.6766
LNCaP-VC 15 μM ITX5061 vs. LNCaP-SPRY2 20 μM ITX5061	0.9989
LNCaP-SPRY2 15 μM ITX5061 vs. LNCaP-VC 20 μM ITX5061	0.0182
LNCaP-SPRY2 15 μM ITX5061 vs. LNCaP-SPRY2 20 μM ITX5061	0.9464
LNCaP-VC 20 μM ITX5061 vs. LNCaP-SPRY2 20 μM ITX5061	0.601

4

LNCaP-VC CTRL vs. LNCaP-SPRY2 VC	<0.0001

		LNCaP-VC CTRL vs. LNCaP-VC 10µM ITX5061	<0.0001
		LNCaP-VC CTRL vs. LNCaP-SPRY2 10 µM ITX5061	<0.0001
		LNCaP-VC CTRL vs. LNCaP-VC 15 µM ITX5061	<0.0001
		LNCaP-VC CTRL vs. LNCaP-SPRY2 15 µM ITX5061	<0.0001
		LNCaP-VC CTRL vs. LNCaP-VC 20 µM ITX5061	<0.0001
		LNCaP-VC CTRL vs. LNCaP-SPRY2 20 µM ITX5061	<0.0001
		LNCaP-SPRY2 VC vs. LNCaP-VC 10 µM ITX5061	<0.0001
		LNCaP-SPRY2 VC vs. LNCaP-SPRY2 10 µM ITX5061	<0.0001
		LNCaP-SPRY2 VC vs. LNCaP-VC 15 µM ITX5061	<0.0001
		LNCaP-SPRY2 VC vs. LNCaP-VC 20 µM ITX5061	<0.0001
		LNCaP-SPRY2 VC vs. LNCaP-SPRY2 20 µM ITX5061	<0.0001
		LNCaP-VC 10 µM ITX5061 vs. LNCaP-SPRY2 10 µM ITX5061	<0.0001
		LNCaP-VC 10 µM ITX5061 vs. LNCaP-VC 15 µM ITX5061	0.9667
		LNCaP-VC 10 µM ITX5061 vs. LNCaP-SPRY2 15 µM ITX5061	0.1117
		LNCaP-VC 10 µM ITX5061 vs. LNCaP-VC 20 µM ITX5061	0.0004
		LNCaP-VC 10 µM ITX5061 vs. LNCaP-SPRY2 20 µM ITX5061	<0.0001
		LNCaP-SPRY2 10 µM ITX5061 vs. LNCaP-VC 15 µM ITX5061	<0.0001
		LNCaP-SPRY2 10 µM ITX5061 vs. LNCaP-SPRY2 15 µM ITX5061	0.6264
		LNCaP-SPRY2 10 µM ITX5061 vs. LNCaP-VC 20 µM ITX5061	0.0105
		LNCaP-SPRY2 10 µM ITX5061 vs. LNCaP-SPRY2 20 µM ITX5061	<0.0001
		LNCaP-VC 15 µM ITX5061 vs. LNCaP-SPRY2 15 µM ITX5061	<0.0001
		LNCaP-VC 15 µM ITX5061 vs. LNCaP-VC 20 µM ITX5061	0.5243
		LNCaP-VC 15 µM ITX5061 vs. LNCaP-SPRY2 20 µM ITX5061	0.0155
		LNCaP-SPRY2 15 µM ITX5061 vs. LNCaP-VC 20 µM ITX5061	<0.0001
		LNCaP-SPRY2 15 µM ITX5061 vs. LNCaP-SPRY2 20 µM ITX5061	0.713
		LNCaP-VC 20 µM ITX5061 vs. LNCaP-SPRY2 20 µM ITX5061	0.0007
Figure EV5L	ANOVA Tukey's test	0	
		VC vs. 10µM ITX5061	>0.9999
		VC vs. 15µM ITX5061	>0.9999
		VC vs. 20µM ITX5061	>0.9999
		10µM ITX5061 vs. 15µM ITX5061	>0.9999
		10µM ITX5061 vs. 20µM ITX5061	>0.9999
		15µM ITX5061 vs. 20µM ITX5061	>0.9999
		2	
		VC vs. 10µM ITX5061	0.0786
		VC vs. 15µM ITX5061	<0.0001

		VC vs. 20µM ITX5061	<0.0001
		10µM ITX5061 vs. 15µM ITX5061	0.0153
		10µM ITX5061 vs. 20µM ITX5061	0.0006
		15µM ITX5061 vs. 20µM ITX5061	0.5623
		4	
		VC vs. 10µM ITX5061	<0.0001
		VC vs. 15µM ITX5061	<0.0001
		VC vs. 20µM ITX5061	<0.0001
		10µM ITX5061 vs. 15µM ITX5061	0.0017
		10µM ITX5061 vs. 20µM ITX5061	<0.0001
		15µM ITX5061 vs. 20µM ITX5061	0.0006
Figure EV5N	Unpaired Two- tailed t test	Nsi Mock Vehicle Vs Nsi ADT Vehicle:	
		Nuclear AR	0.00014056
		Cleaved Capase 3	0.00609197
		Nsi Mock ITX5061 Vs Nsi ADT ITX5061:	
		Nuclear AR	0.00014244
		Cleaved Capase 3	0.007401964
		Pool Mock ITX5061 Vs Pool ADT ITX5061 :	
		Nuclear AR	0.000237895
		Cleaved Capase 3	0.002315735
Appendix Figure S1E	Unpaired Two- tailed t test	Proximal Mets	0.001184277
		Distal mets (Thoracic Mets)	0.007256289
		Distal mets (Lungs)	0.008962484
Appendix Figure S2A	ANOVA Tukey's test	Nsi Mock vs. Pool (SPRY2 KD) Mock	0.3753
		Nsi Mock vs. Nsi ADT	0.0036
		Nsi Mock vs. Pool (SPRY2 KD) ADT	0.001
		Pool (SPRY2 KD) Mock vs. Nsi ADT	0.0329
		Pool (SPRY2 KD) Mock vs. Pool (SPRY2 KD) ADT	0.0077
		Nsi ADT vs. Pool (SPRY2 KD) ADT	0.7078
Appendix Figure S2C	ANOVA Tukey's test	Nsi Mock vs. Nsi ADT	0.9747
		Nsi Mock vs. Pool Mock	>0.9999
		Nsi Mock vs. Pool ADT	0.0074
		Nsi ADT vs. Pool Mock	0.9802
		Nsi ADT vs. Pool ADT	0.0125

		Pool Mock vs. Pool ADT	0.0077
Appendix Figure S2D	Unpaired Two- tailed t test	IL6 vs Ctrl	0.0287
Appendix Figure S2E	Unpaired Two- tailed t test	ADT Vs Mock	<0.0001
Appendix Figure S2K	ANOVA Tukey's test	Liver tumour vs. Normal	0.0073
		Liver tumour vs. ADT	0.0234
		Liver tumour vs. Nsi VC	0.0137
		Liver tumour vs. Nsi + ITX5061	0.028
		Liver tumour vs. Nsi Simvastatin	0.0039
		Liver tumour vs. Nsi ADT	0.0198
		Liver tumour vs. Nsi ADT + ITX5061	0.016
		Liver tumour vs. Nsi ADT + Simvastatin	0.0031
		Liver tumour vs. Pool	0.0141
		Liver tumour vs. Pool ITX5061	0.0076
		Liver tumour vs. Pool Simvastatin	0.0129
		Liver tumour vs. Pool ADT	>0.9999
		Liver tumour vs. Pool ADT + ITX5061	0.0027
		Liver tumour vs. Pool ADT + Simvastatin	0.2344
		Normal vs. ADT	>0.9999
		Normal vs. Nsi VC	>0.9999
		Normal vs. Nsi + ITX5061	0.9995
		Normal vs. Nsi Simvastatin	>0.9999
		Normal vs. Nsi ADT	>0.9999
		Normal vs. Nsi ADT + ITX5061	>0.9999
		Normal vs. Nsi ADT + Simvastatin	>0.9999
		Normal vs. Pool	>0.9999
		Normal vs. Pool ITX5061	>0.9999
		Normal vs. Pool Simvastatin	>0.9999
		Normal vs. Pool ADT	0.005
		Normal vs. Pool ADT + ITX5061	>0.9999
		Normal vs. Pool ADT + Simvastatin	0.8098
		ADT vs. Nsi VC	>0.9999
		ADT vs. Nsi + ITX5061	>0.9999
		ADT vs. Nsi Simvastatin	>0.9999
		ADT vs. Nsi ADT	>0.9999
		ADT vs. Nsi ADT + ITX5061	>0.9999
		ADT vs. Nsi ADT + Simvastatin	>0.9999
		ADT vs. Pool	>0.9999
		ADT vs. Pool ITX5061	>0.9999
		ADT vs. Pool Simvastatin	>0.9999

ADT vs. Pool ADT	0.0183
ADT vs. Pool ADT + ITX5061	>0.9999
ADT vs. Pool ADT + Simvastatin	0.9752
Nsi VC vs. Nsi + ITX5061	>0.9999
Nsi VC vs. Nsi Simvastatin	>0.9999
Nsi VC vs. Nsi ADT	>0.9999
Nsi VC vs. Nsi ADT + ITX5061	>0.9999
Nsi VC vs. Nsi ADT + Simvastatin	>0.9999
Nsi VC vs. Pool	>0.9999
Nsi VC vs. Pool ITX5061	>0.9999
Nsi VC vs. Pool Simvastatin	>0.9999
Nsi VC vs. Pool ADT	0.0086
Nsi VC vs. Pool ADT + ITX5061	>0.9999
Nsi VC vs. Pool ADT + Simvastatin	0.9771
Nsi + ITX5061 vs. Nsi Simvastatin	>0.9999
Nsi + ITX5061 vs. Nsi ADT	>0.9999
Nsi + ITX5061 vs. Nsi ADT + ITX5061	>0.9999
Nsi + ITX5061 vs. Nsi ADT + Simvastatin	0.9995
Nsi + ITX5061 vs. Pool	>0.9999
Nsi + ITX5061 vs. Pool ITX5061	>0.9999
Nsi + ITX5061 vs. Pool Simvastatin	>0.9999
Nsi + ITX5061 vs. Pool ADT	0.0195
Nsi + ITX5061 vs. Pool ADT + ITX5061	>0.9999
Nsi + ITX5061 vs. Pool ADT + Simvastatin	0.9979
Nsi Simvastatin vs. Nsi ADT	>0.9999
Nsi Simvastatin vs. Nsi ADT + ITX5061	>0.9999
Nsi Simvastatin vs. Nsi ADT + Simvastatin	>0.9999
Nsi Simvastatin vs. Pool	>0.9999
Nsi Simvastatin vs. Pool ITX5061	>0.9999
Nsi Simvastatin vs. Pool Simvastatin	>0.9999
Nsi Simvastatin vs. Pool ADT	0.0018
Nsi Simvastatin vs. Pool ADT + ITX5061	>0.9999
Nsi Simvastatin vs. Pool ADT + Simvastatin	0.8697
Nsi ADT vs. Nsi ADT + ITX5061	>0.9999
Nsi ADT vs. Nsi ADT + Simvastatin	>0.9999
Nsi ADT vs. Pool	>0.9999
Nsi ADT vs. Pool ITX5061	>0.9999
Nsi ADT vs. Pool Simvastatin	>0.9999
Nsi ADT vs. Pool ADT	0.0131
Nsi ADT vs. Pool ADT + ITX5061	>0.9999
Nsi ADT vs. Pool ADT + Simvastatin	0.9922
Nsi ADT + ITX5061 vs. Nsi ADT + Simvastatin	>0.9999
Nsi ADT + ITX5061 vs. Pool	>0.9999
Nsi ADT + ITX5061 vs. Pool ITX5061	>0.9999

		Nsi ADT + ITX5061 vs. Pool Simvastatin	>0.9999
		Nsi ADT + ITX5061 vs. Pool ADT	0.0103
		Nsi ADT + ITX5061 vs. Pool ADT + ITX5061	>0.9999
		Nsi ADT + ITX5061 vs. Pool ADT + Simvastatin	0.9852
		Nsi ADT + Simvastatin vs. Pool	>0.9999
		Nsi ADT + Simvastatin vs. Pool ITX5061	>0.9999
		Nsi ADT + Simvastatin vs. Pool Simvastatin	>0.9999
		Nsi ADT + Simvastatin vs. Pool ADT	0.0016
		Nsi ADT + Simvastatin vs. Pool ADT + ITX5061	>0.9999
		Nsi ADT + Simvastatin vs. Pool ADT + Simvastatin	0.7434
		Pool vs. Pool ITX5061	>0.9999
		Pool vs. Pool Simvastatin	>0.9999
		Pool vs. Pool ADT	0.0089
		Pool vs. Pool ADT + ITX5061	>0.9999
		Pool vs. Pool ADT + Simvastatin	0.9791
		Pool ITX5061 vs. Pool Simvastatin	>0.9999
		Pool ITX5061 vs. Pool ADT	0.0044
		Pool ITX5061 vs. Pool ADT + ITX5061	>0.9999
		Pool ITX5061 vs. Pool ADT + Simvastatin	0.9217
		Pool Simvastatin vs. Pool ADT	0.0081
		Pool Simvastatin vs. Pool ADT + ITX5061	>0.9999
		Pool Simvastatin vs. Pool ADT + Simvastatin	0.9737
		Pool ADT vs. Pool ADT + ITX5061	0.001
		Pool ADT vs. Pool ADT + Simvastatin	0.2213
		Pool ADT + ITX5061 vs. Pool ADT + Simvastatin	0.847
Appendix Figure S2L	ANOVA Tukey's test	Mock vs. 48 hrs post-ADT	0.0784
		Mock vs. 4 weeks post-ADT	0.0005
		48 hrs post-ADT vs. 4 weeks post-ADT	0.0048
Appendix Figure S2N	ANOVA Tukey's test	Nuclear AR	
		Nsi Mock Vehicle vs. Nsi Mock Simvastatin	>0.9999
		Nsi Mock Vehicle vs. Nsi ADT Vehicle	<0.0001
		Nsi Mock Vehicle vs. Nsi ADT Simvastatin	<0.0001
		Nsi Mock Vehicle vs. Pool Mock Vehicle	0.9997
		Nsi Mock Vehicle vs. Pool Mock Simvastation	0.0061
		Nsi Mock Vehicle vs. Pool ADT Vehicle	0.6911
		Nsi Mock Vehicle vs. Pool ADT Simvastatin	<0.0001
		Nsi Mock Simvastatin vs. Nsi ADT Vehicle	<0.0001
		Nsi Mock Simvastatin vs. Nsi ADT Simvastatin	<0.0001
		Nsi Mock Simvastatin vs. Pool Mock Vehicle	0.999
		Nsi Mock Simvastatin vs. Pool Mock Simvastation	0.0078
		Nsi Mock Simvastatin vs. Pool ADT Vehicle	0.7475

Nsi Mock Simvastatin vs. Pool ADT Simvastatin	<0.0001
Nsi ADT Vehicle vs. Nsi ADT Simvastatin	0.9702
Nsi ADT Vehicle vs. Pool Mock Vehicle	<0.0001
Nsi ADT Vehicle vs. Pool Mock Simvastation	<0.0001
Nsi ADT Vehicle vs. Pool ADT Vehicle	<0.0001
Nsi ADT Vehicle vs. Pool ADT Simvastatin	0.9996
Nsi ADT Simvastatin vs. Pool Mock Vehicle	<0.0001
Nsi ADT Simvastatin vs. Pool Mock Simvastation	<0.0001
Nsi ADT Simvastatin vs. Pool ADT Vehicle	<0.0001
Nsi ADT Simvastatin vs. Pool ADT Simvastatin	0.9995
Pool Mock Vehicle vs. Pool Mock Simvastation	0.0017
Pool Mock Vehicle vs. Pool ADT Vehicle	0.3967
Pool Mock Vehicle vs. Pool ADT Simvastatin	<0.0001
Pool Mock Simvastation vs. Pool ADT Vehicle	0.2835
Pool Mock Simvastation vs. Pool ADT Simvastatin	<0.0001
Pool ADT Vehicle vs. Pool ADT Simvastatin	<0.0001

Cleaved Capase 3

Nsi Mock Vehicle vs. Nsi Mock Simvastatin	0.9393
Nsi Mock Vehicle vs. Nsi ADT Vehicle	<0.0001
Nsi Mock Vehicle vs. Nsi ADT Simvastatin	<0.0001
Nsi Mock Vehicle vs. Pool Mock Vehicle	0.9998
Nsi Mock Vehicle vs. Pool Mock Simvastation	0.4755
Nsi Mock Vehicle vs. Pool ADT Vehicle	0.9889
Nsi Mock Vehicle vs. Pool ADT Simvastatin	<0.0001
Nsi Mock Simvastatin vs. Nsi ADT Vehicle	<0.0001
Nsi Mock Simvastatin vs. Nsi ADT Simvastatin	<0.0001
Nsi Mock Simvastatin vs. Pool Mock Vehicle	0.7405
Nsi Mock Simvastatin vs. Pool Mock Simvastation	0.0562
Nsi Mock Simvastatin vs. Pool ADT Vehicle	>0.9999
Nsi Mock Simvastatin vs. Pool ADT Simvastatin	<0.0001
Nsi ADT Vehicle vs. Nsi ADT Simvastatin	0.3702
Nsi ADT Vehicle vs. Pool Mock Vehicle	<0.0001
Nsi ADT Vehicle vs. Pool Mock Simvastation	0.007
Nsi ADT Vehicle vs. Pool ADT Vehicle	<0.0001
Nsi ADT Vehicle vs. Pool ADT Simvastatin	>0.9999
Nsi ADT Simvastatin vs. Pool Mock Vehicle	<0.0001
Nsi ADT Simvastatin vs. Pool Mock Simvastation	<0.0001
Nsi ADT Simvastatin vs. Pool ADT Vehicle	<0.0001
Nsi ADT Simvastatin vs. Pool ADT Simvastatin	0.4977
Pool Mock Vehicle vs. Pool Mock Simvastation	0.7597
Pool Mock Vehicle vs. Pool ADT Vehicle	0.8914
Pool Mock Vehicle vs. Pool ADT Simvastatin	<0.0001
Pool Mock Simvastation vs. Pool ADT Vehicle	0.1108

Pool Mock Simvastation vs. Pool ADT Simvastatin	0.0039
Pool ADT Vehicle vs. Pool ADT Simvastatin	<0.0001
