# Supplementary material for: Sprouty2 loss‐induced IL6 drives castration‐resistant prostate cancer through scavenger receptor B1
Source: EMBO Mol Med. 2018 Mar 14;10(4):e8347. doi: 10.15252/emmm.201708347 (PMC5887544; doi:10.15252/emmm.201708347)
Supplement: Supplementary file 2 — Expanded View Figures PDF [file EMMM-10-e8347-s002.pdf]

Expanded View Figures

Figure EV1. SPRY2 deficiency mediates CRPC.

A Kaplan–Meier plot for relapse-free survival in TCGA prostate cancer 2015 dataset showing cases with low SPRY2 mRNA expression *z*-scores (RNA seq V2 RSEM) (EXP < −1.3) n = 48 and rest of the cases with higher SPRY2 expression (n = 443); log-rank (Mantel–Cox) test; P = 0.034.
B Immunostaining for PTEN in HN and CRPC matched clinical prostate cancer sections represented as IHC (immunohistochemistry) scores.
C Scatter plot showing correlation of PTEN and SPRY2 IHC scores in CRPC samples.
D Kaplan–Meier plot for overall survival of NPS mice treated with ADT after palpable tumour was detected after 50 weeks (mock = 7; ADT = 6). After 50 weeks, palpable tumour was detected (black dotted line). The mice were then randomised into mock treatment or ADT treatment (red dotted line).
E Representative images of prostate tumours (PT) from mock and ADT-treated NPS mice 1 month after ADT.
F Representative images of prostate tumours from mock and ADT-treated NPS mice at clinical endpoints (~77–115 weeks).
G IHC quantifications in NPS tumours (n = 3 mice per group; $^*P < 0.05$; ANOVA Sidak's test).
H Prostate tissue weights at 1 month following mock or ADT treatment in *Nkx3.1 Pten*$^{+/+}$ *Spry2*$^{+/+}$ wild-type (WT), *Nkx3.1 Pten*$^{fl/+}$ and *Nkx3.1 Spry2*$^{fl/+}$ mice (n = 3 mice per group; $^*P < 0.05$ compared to respective mocks; ANOVA Sidak's test).
I Representative immunostained images of mouse prostate sections from *Nkx3.1 Pten*$^{+/+}$ *Spry2*$^{+/+}$ wild-type (WT), *Nkx3.1 Pten*$^{fl/+}$ and *Nkx3.1 Spry2*$^{fl/+}$ mice 1 month following mock or ADT treatment. Scale bar = 10 μm.
J IHC quantifications for *Nkx3.1 Pten*$^{+/+}$ *Spry2*$^{+/+}$ wild-type (WT), *Nkx3.1 Pten*$^{fl/+}$ and *Nkx3.1 Spry2*$^{fl/+}$ mock- and ADT-treated control prostate sections (n = 3 mice per group; $^*P < 0.05$ compared to respective mock controls; ANOVA Tukey's test).
K Representative immunoblot images for indicated proteins from cell lysates of a panel of prostate cancer cell lines along with non-tumorigenic RWPE-1 prostate epithelial cells. HSC70 is used as loading control.

Data Information: In (B, C, G, H, J), each data point represents one independent sample. In (B, G, H, J), the data are presented as mean ± SD.
Source data are available online for this figure.

 © 2018 The Authors

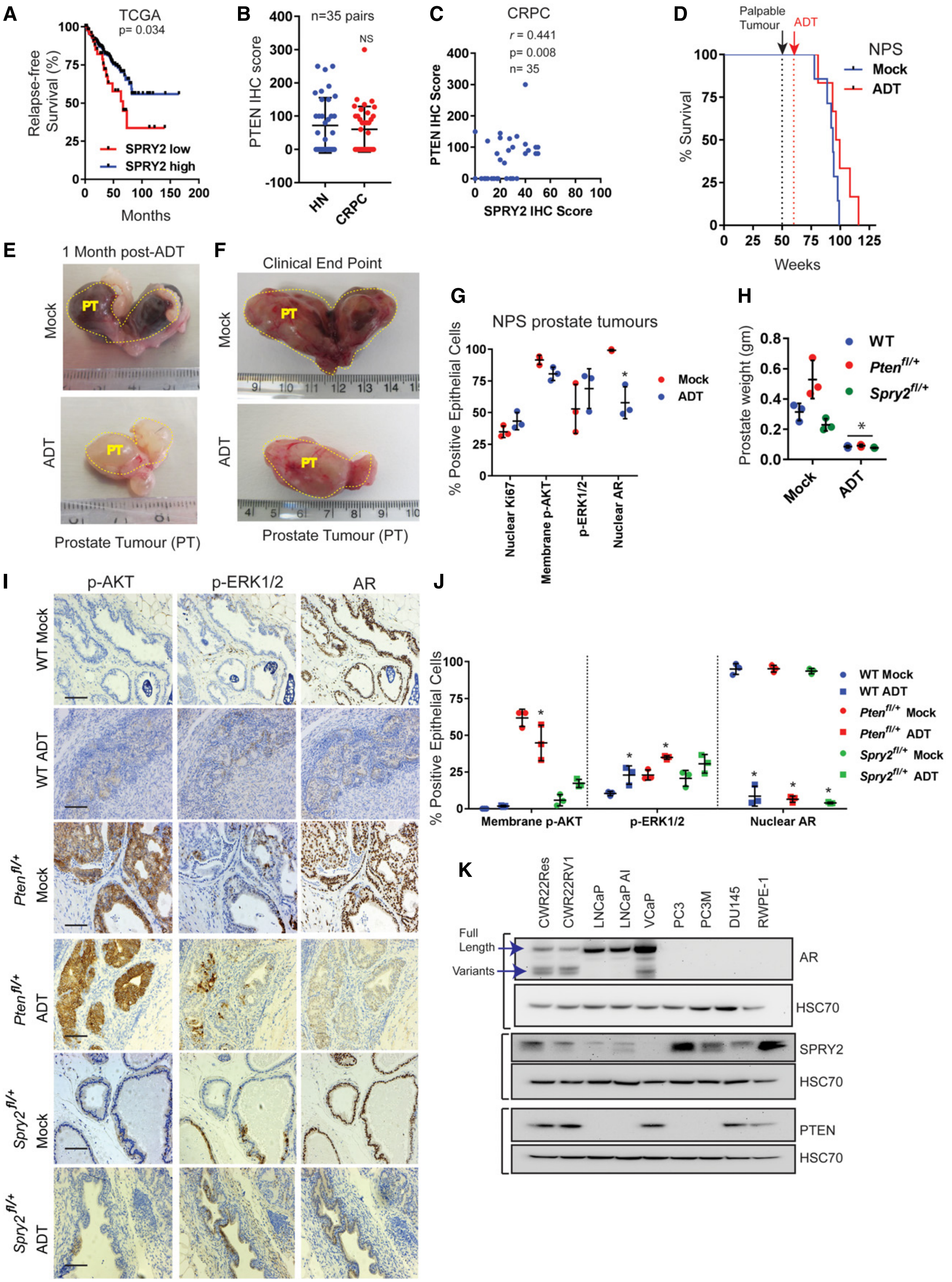
A
TCGA
p= 0.034
Relapse-free Survival (%)
SPRY2 low
SPRY2 high
Months
B
n=35 pairs
NS
PTEN IHC score
HN
CRPC
C
CRPC
r = 0.441
p= 0.008
n= 35
PTEN IHC Score
SPRY2 IHC Score
D
Palpable Tumour
ADT
NPS
Mock
ADT
% Survival
Weeks
E
1 Month post-ADT
Mock
ADT
PT
Prostate Tumour (PT)
F
Clinical End Point
Mock
ADT
PT
Prostate Tumour (PT)
G
NPS prostate tumours
% Positive Epithelial Cells
Mock
ADT
Nuclear Ki67
Membrane p-AKT
p-ERK1/2
Nuclear AR
H
Prostate weight (gm)
WT
Pten fl/+
Spry2 fl/+
Mock
ADT
I
p-AKT
p-ERK1/2
AR
WT Mock
WT ADT
Pten fl/+ Mock
Pten fl/+ ADT
Spry2 fl/+ Mock
Spry2 fl/+ ADT
J
% Positive Epithelial Cells
Membrane p-AKT
p-ERK1/2
Nuclear AR
WT Mock
WT ADT
Pten fl/+ Mock
Pten fl/+ ADT
Spry2 fl/+ Mock
Spry2 fl/+ ADT
K
CWR22Res
CWR22RV1
LNCaP
LNCaP AI
VCaP
PC3
PC3M
DU145
RWPE-1
Full Length
Variants
AR
HSC70
SPRY2
HSC70
PTEN
HSC70

Figure EV1.

Figure EV2. SPRY2 deficiency leads to CRPC.

A Representative immunoblot images for indicated proteins from cell lysates of control and SPRY2-overexpressing LNCaP cells. HSC70 is used as loading control.
B Relative levels of AR full-length and AR-V7 transcripts in indicated cell lines (n = 3; *P < 0.05 ANOVA Sidak's test).
C Growth rate of CWR22Res and CWR22RV2 cells relative to Day 0 (T0) in hormone-proficient (FBS) medium and hormone-depleted (CSS) medium (n = 3; *P < 0.05; ANOVA Sidak's test).
D The relative quantitation of SPRY2 mRNA in CWR22Res prostate cancer cells stably transfected with non-silencing scrambled shRNA (Nsi) and shRNA targeting SPRY2 (CL3 and Pool SPRY2 knockdown clones) (n = 3; *P < 0.05 compared to Nsi; ANOVA Dunnett's test).
E Ultrasound-based CWR22Res prostate orthograft tumour volume measurements. Each animal is represented by single line and each observation by a data point (n = 5 mice per group; *P < 0.05 at 60 days' time point; ANOVA Sidak's test).
F Representative ultrasound images of mock- and ADT-treated CWR22Res prostate orthografts (POs) at 60 days' time point (E). The dotted red lines highlight the POs. The cystic region observed in the ultrasound in ADT-treated orthograft may represent necrotic central core of the orthografts as shown in (K).
G Approximately 14×10^6 of CWR22Res-derived cells (Nsi and SPRY2 KD clones—CL3 and Pool, respectively) were injected in one of the anterior prostate lobes in CD-1 nude mice. At day 30 post-intra-prostatic injections, mice were randomised for further treatments with either sham surgical incision (mock) or androgen deprivation therapy (ADT) by bilateral orchiectomy based on the abdominal palpation for the tumour burden.
H Kaplan–Meier plot for overall survival of CD-1 mice after intra-prostatic injection of CWR22Res prostate cancer cells (Nsi control or CL3/Pool SPRY2 KD clones). The mice were randomised and treated with sham surgery or ADT (by castration) at 30 days post-injections when the prostate orthografts were palpable. The clinical end point as per project licence was used as the measure for survival of the tumour-bearing mice. Yellow line indicates refined 60 days protocol defined from this experiment (n = 5 mice per group; *P < 0.05 between ADT-treated mice; $^{\#}P$ < 0.05 respective orthograft-bearing mock- versus ADT-treated mice; log-rank Mantel–Cox test).
I Analysis of body weights without the weights of the respective orthografts following ADT and mock treatments (n = 5 mice per group; *P < 0.05 ANOVA Tukey's test).
J Representative images of indicated CWR22Res orthografts from mock and ADT-treated mice (n = 5 mice per group).
K Representative images of prostate orthograft (intact and halved). The black dotted area in the halved prostate orthograft marks the necrotic core. Representative H&E images of a prostate orthograft depicting the use of image analyser for calculating the percentage of the necrotic area (red area) normalised to total tumour area (blue dotted area). Insert image shows necrotic area (NA) surrounding the tumour epithelium (TE). Scale bar = 10 μm.
L Relative levels of AR full-length and AR-V7 transcripts in Nsi control and CL3/Pool SPRY2 KD CWR22Res cell lines (n = 3).
M Representative immunoblot images for indicated proteins from cell lysates of CWR22Res orthografts. HSC70 is used as loading control.
N Representative immunoblot images for indicated proteins from cell lysates of CWR22Res-derived cells with different SPRY2 and PTEN expression. GAPDH is used as loading control.
O Relative survival measured by WST-1 assay in CWR22Res cells with transient knockdown of PTEN grown in CSS-containing medium (n = 3; *P < 0.05 compared to Nsi siCTRL; $^{\#}P$ < 0.05 compared to Nsi siPTEN; ANOVA Tukey's test).

Data Information: In (E, I), each data point represents one independent observation. In (C), each data point is mean of three independent observations. In (B–D, I, L, O), the data are presented as mean ± SD.
Source data are available online for this figure.

© 2018 The Authors

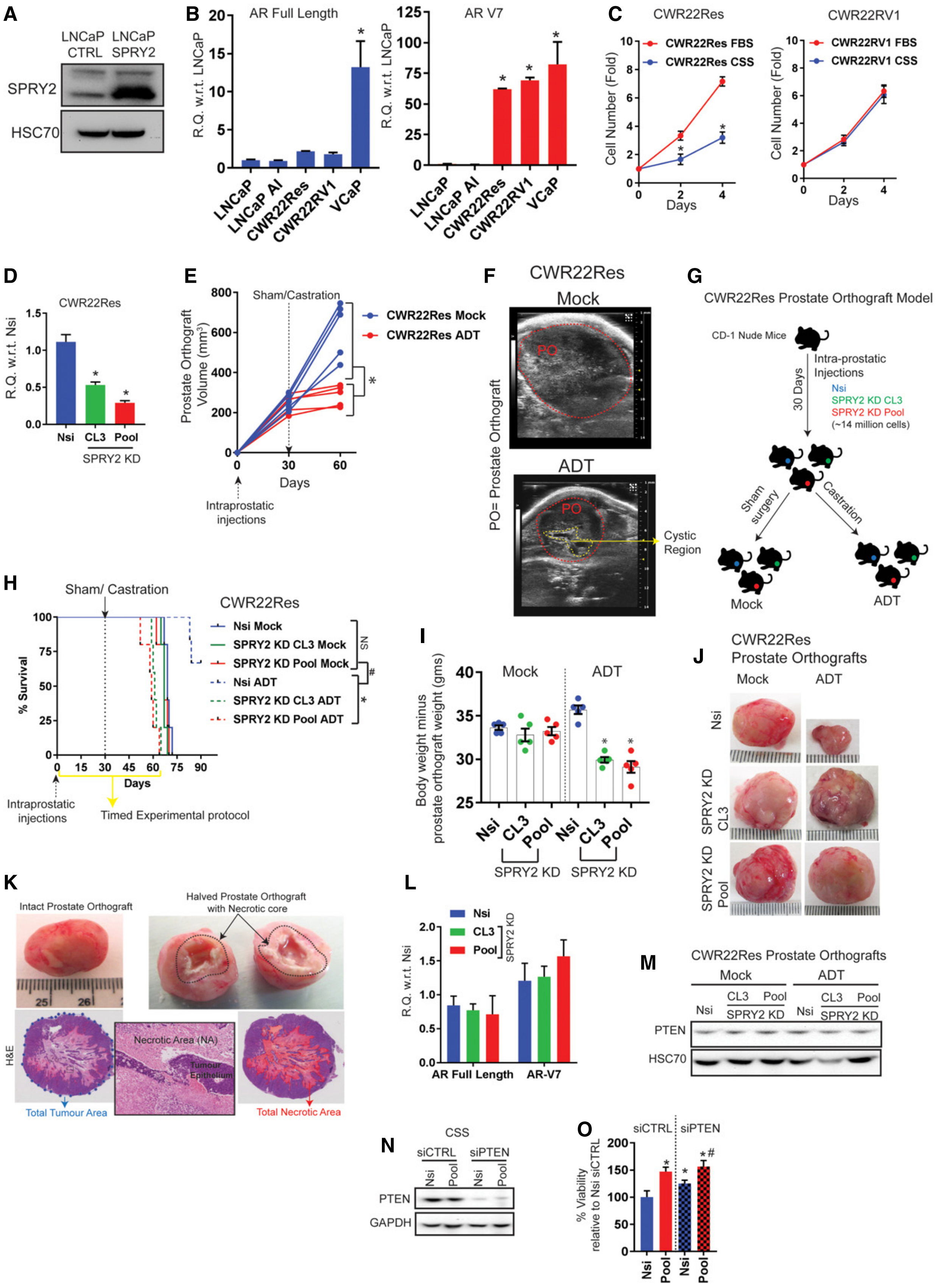
A
LNCaP CTRL
LNCaP SPRY2
SPRY2
HSC70
B
AR Full Length
R.Q. w.r.t. LNCaP
AR V7
LNCaP
LNCaP AI
CWR22Res
CWR22RV1
VCaP
C
CWR22Res
CWR22Res FBS
CWR22Res CSS
Cell Number (Fold)
Days
CWR22RV1
CWR22RV1 FBS
CWR22RV1 CSS
D
CWR22Res
R.Q. w.r.t. Nsi
Nsi
CL3
Pool
SPRY2 KD
E
Sham/Castration
Prostate Orthograft Volume (mm³)
CWR22Res Mock
CWR22Res ADT
Days
Intraprostatic injections
F
CWR22Res
Mock
PO
ADT
PO= Prostate Orthograft
Cystic Region
G
CWR22Res Prostate Orthograft Model
CD-1 Nude Mice
Intra-prostatic Injections
30 Days
Nsi
SPRY2 KD CL3
SPRY2 KD Pool
(~14 million cells)
Sham surgery
Castration
Mock
ADT
H
Sham/ Castration
CWR22Res
% Survival
Nsi Mock
SPRY2 KD CL3 Mock
SPRY2 KD Pool Mock
Nsi ADT
SPRY2 KD CL3 ADT
SPRY2 KD Pool ADT
NS
Days
Intraprostatic injections
Timed Experimental protocol
I
Body weight minus prostate orthograft weight (gms)
Mock
ADT
Nsi
CL3
Pool
SPRY2 KD
J
CWR22Res Prostate Orthografts
Mock
ADT
Nsi
SPRY2 KD CL3
SPRY2 KD Pool
K
Intact Prostate Orthograft
Halved Prostate Orthograft with Necrotic core
H&E
Necrotic Area (NA)
Tumour Epithelium
Total Tumour Area
Total Necrotic Area
L
Nsi
CL3
Pool
SPRY2 KD
R.Q. w.r.t. Nsi
AR Full Length
AR-V7
M
CWR22Res Prostate Orthografts
Mock
ADT
Nsi
CL3
Pool
SPRY2 KD
PTEN
HSC70
N
CSS
siCTRL
siPTEN
Nsi
Pool
PTEN
GAPDH
O
siCTRL
siPTEN
% Viability relative to Nsi siCTRL
Nsi
Pool

Figure EV2.

Figure EV3. SPRY2 deficiency mediates androgen autonomous CRPC.

A Representative images of indicated CWR22Res prostate orthograft sections immunostained for AR and the apoptotic marker cleaved caspase 3 from mock- or ADT-treated mice (n = 3 mice analysed per group; scale bar = 10 μm).

B IHC quantification of indicated markers as presented in (A) (n = 3 mice analysed per group; $^*P < 0.05$ compared to respective mock controls; $^\#P < 0.05$ compared to all groups ANOVA Sidak's test).

C Relative quantitation of human HSD3B1, CYP17A1 and HSD17B1 mRNA in CWR22Res Nsi control and SPRY2 KD clones (CL3 and Pool) cells (n = 3; $^*P < 0.05$; unpaired two-tailed Student's t-test).

D Representative immunoblot images for HSD3B1 from cell lysates of indicated CWR22Res cells. HSC70 is used as loading control.

E Relative quantitation of human CYP17A1 mRNA in CWR22Res prostate orthografts (n = 3).

F Representative immunoblot images for HSD3B1 from tumour lysates from mock- and ADT-treated NPS mice. HSC70 is used as loading control.

G Relative quantitation of human HSD3B1, CYP17A1 and HSD17B1 mRNA in control and SPRY2-expressing LNCaP cells (n = 3; $^*P$ = 0.0069; unpaired two-tailed Student's t-test).

H Representative immunoblot images for HSD3B1 from VCaP prostate orthograft lysates from castrated (ADT) and uncastrated (control) mice. HSC70 is used as loading control.

I, J Growth rate of (I) control and SPRY2-expressing LNCaP cells & (J) Nsi control and Pool SPRY2 KD CWR22Res cells relative to Day 0 (T0) in hormone-proficient (FBS) medium and hormone-depleted (CSS) medium (n = 3; $^*P < 0.05$; ANOVA Tukey's test).

K Representative immunoblot images for HSD3B1 from indicated CWR22Res cells with HSD3B1 knockout. HSC70 is used as loading control.

L Growth rate of Nsi control and Pool SPRY2 KD CWR22Res cells with HSD3B1 knockout relative to Day 0 (T0) in hormone-proficient (FBS) medium.

M Testosterone measured using ELISA in indicated CWR22Res-derived cells with HSD3B1 knockout grown in medium with 10% FBS or 10% CSS (hormone-depleted serum) (n = 3; $^*P < 0.05$ compared to Nsi FBS ANOVA Dunnett's test).

N Representative immunoblot images for indicated proteins from cell lysates of indicated prostate cancer cell lines and non-tumorigenic RWPE-1 cells. HSC70 is used as loading control.

O Relative quantitation of HER2 mRNA in Nsi and Pool (SPRY2 KD) CWR22Res prostate cancer cells with stable expression of shSc (shScram) or shHER2 (n = 3; $^*P < 0.05$ shHER2 compared to respective shSc; unpaired two-tailed Student's t-test).

Data Information: In (B, E), each data point represents one independent observation. In (I, J, L), each data point is mean of three independent observations. In (B, C, E, G, I, J, L, M, O), the data are presented as mean ± SD.
Source data are available online for this figure.

 © 2018 The Authors

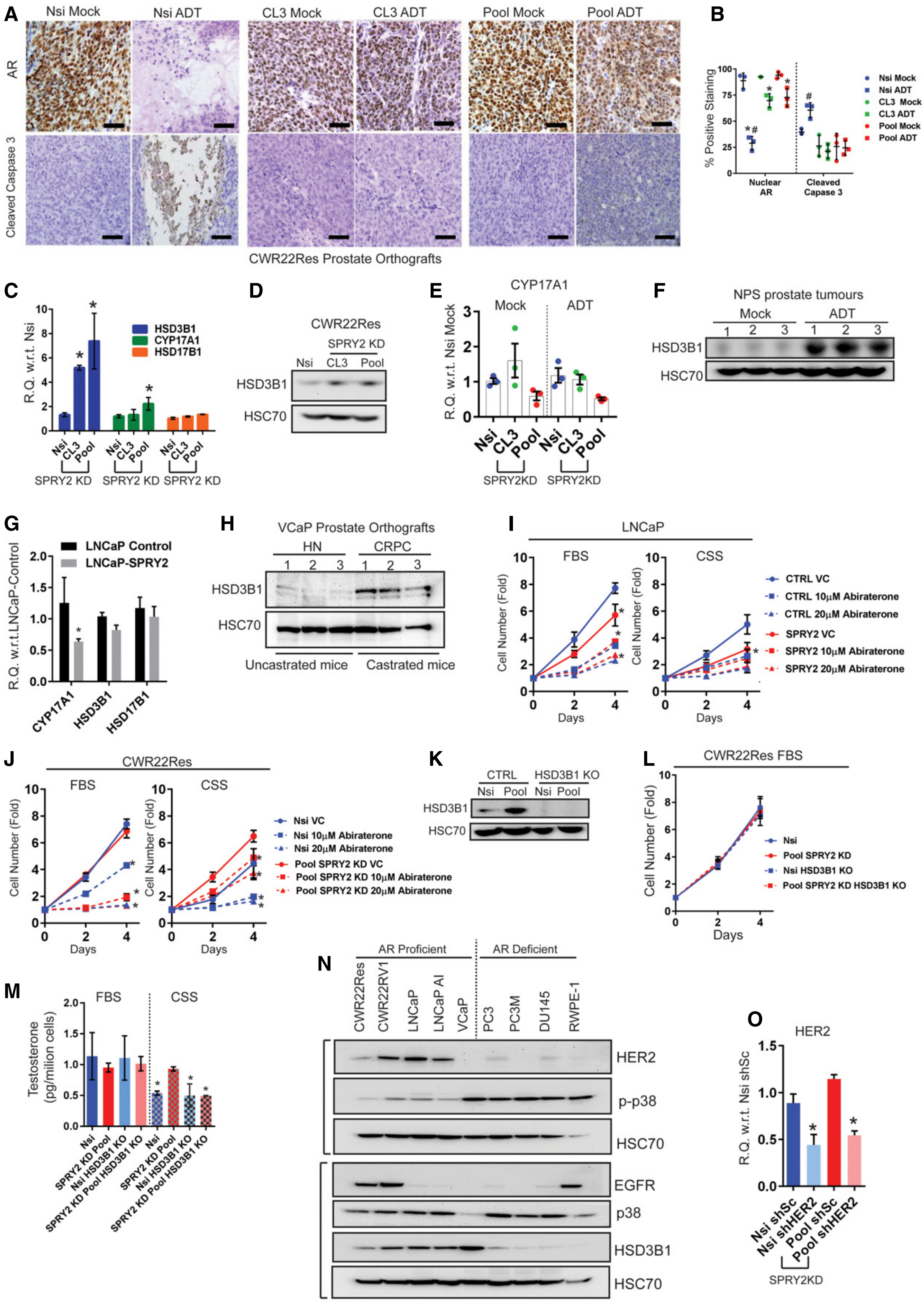
A
Nsi Mock
Nsi ADT
CL3 Mock
CL3 ADT
Pool Mock
Pool ADT
AR
Cleaved Caspase 3
CWR22Res Prostate Orthografts
B
% Positive Staining
Nuclear AR
Cleaved Capase 3
Nsi Mock
Nsi ADT
CL3 Mock
CL3 ADT
Pool Mock
Pool ADT
C
R.Q. w.r.t. Nsi
HSD3B1
CYP17A1
HSD17B1
SPRY2 KD
D
CWR22Res
SPRY2 KD
Nsi CL3 Pool
HSD3B1
HSC70
E
CYP17A1
Mock
ADT
R.Q. w.r.t. Nsi Mock
SPRY2KD
F
NPS prostate tumours
Mock
ADT
HSD3B1
HSC70
G
LNCaP Control
LNCaP-SPRY2
R.Q. w.r.t.LNCaP-Control
CYP17A1
HSD3B1
HSD17B1
H
VCaP Prostate Orthografts
HN
CRPC
HSD3B1
HSC70
Uncastrated mice
Castrated mice
I
LNCaP
FBS
CSS
Cell Number (Fold)
Days
CTRL VC
CTRL 10μM Abiraterone
CTRL 20μM Abiraterone
SPRY2 VC
SPRY2 10μM Abiraterone
SPRY2 20μM Abiraterone
J
CWR22Res
FBS
CSS
Nsi VC
Nsi 10μM Abiraterone
Nsi 20μM Abiraterone
Pool SPRY2 KD VC
Pool SPRY2 KD 10μM Abiraterone
Pool SPRY2 KD 20μM Abiraterone
K
CTRL
HSD3B1 KO
Nsi Pool Nsi Pool
HSD3B1
HSC70
L
CWR22Res FBS
Nsi
Pool SPRY2 KD
Nsi HSD3B1 KO
Pool SPRY2 KD HSD3B1 KO
M
FBS
CSS
Testosterone (pg/milion cells)
Nsi
SPRY2 KD Pool
Nsi HSD3B1 KO
SPRY2 KD Pool HSD3B1 KO
N
AR Proficient
AR Deficient
CWR22Res
CWR22RV1
LNCaP
LNCaP AI
VCaP
PC3
PC3M
DU145
RWPE-1
HER2
p-p38
HSC70
EGFR
p38
HSD3B1
HSC70
O
HER2
R.Q. w.r.t. Nsi shSc
Nsi shSc
Nsi shHER2
Pool shSc
Pool shHER2
SPRY2KD

Figure EV3.

Figure EV4. SPRY2 deficiency-induced IL6 drives CRPC by elevating tumoral HSD3B1 and cholesterol levels.

- A Human cytokine arrays probed with lysates from Nsi and SPRY2 KD (Pool) CWR22Res prostate orthografts (n = 2). Red boxes highlight the location for IL6 detection.
- B Relative quantitation of IL6 mRNA in control and SPRY2-expressing LNCaP cells (n = 3; *P = 0.0498; unpaired two-tailed Student's t-test).
- C Representative immunoblot images for indicated proteins from cell lysates of indicated prostate cancer cell lines. For the lower panel, lysates were from cells treated for 16 h with the p38 inhibitor SB203580 (20 μM). HSC70 is used as loading control.
- D Representative IL6 immunostained images of clinical hormone-naïve and castration-resistant (CRPC) prostate cancer samples (n = 35). Scale bars = 100 μm.
- E Scatter plot showing correlation of SPRY2 and IL6 IHC scores in CRPC patients with evidence of biochemical relapse.
- F Relative quantitation of IL6 mRNA in indicated cell lines. LNCaP, CWR22Res and DU145 cells were cultured in RPMI medium with 10% FBS. CWR22RV1 cells were cultured in RPMI medium with 10% CSS (hormone-deficient conditions) (n = 3; *$P < 0.05$ ANOVA Tukey's test).
- G Relative quantitation of HSD3B1 mRNA in CWR22Res cells cultured in serum-free medium with and without 100 pmol/ml hIL6 treatment for 24 h (n = 3; *P = 0.0222; unpaired two-tailed Student's t-test).
- H Relative quantitation of IL6 and HSD3B1 mRNA in Pool (SPRY2 KD clone) CWR22Res cells treated with control or anti-IL6-neutralising antibody (0.1 μg/μl) for 16 h (n = 3; *$P < 0.05$, anti-IL6 compared to respective ctrl IgG; unpaired two-tailed Student's t-test).
- I Representative immunoblot images for indicated proteins in lysates from indicated CWR22Res orthografts.
- J Representative images of indicated CWR22Res prostate orthograft sections immunostained for AR and apoptotic marker cleaved caspase 3 from mock or ADT-treated mice (n = 3 mice analysed per group; scale bar = 10 μm).
- K IHC quantification of indicated markers as presented in (J) (n = 3 mice analysed per group; *$P < 0.05$ compared to respective mock controls; $^{\#}P < 0.05$ compared to respective ADT controls ANOVA Tukey's test).
- L Representative H&E and immunostained images of prostate sections from ADT-treated NPS mice with and without anti-IL6 treatment (n = 3 mice analysed per group; scale bar = 10 μm).
- M IHC quantification of indicated markers as presented in (L) (n = 3 mice analysed per group; *$P < 0.05$ compared to respective controls; unpaired two-tailed Student's t-test).

Data Information: In (E), each data point represents one observation. In (K, M), each data point represents one independent observation with error bars as SD. In (B, F–H, K, M), the data are presented as mean ± SD.
Source data are available online for this figure.

 © 2018 The Authors

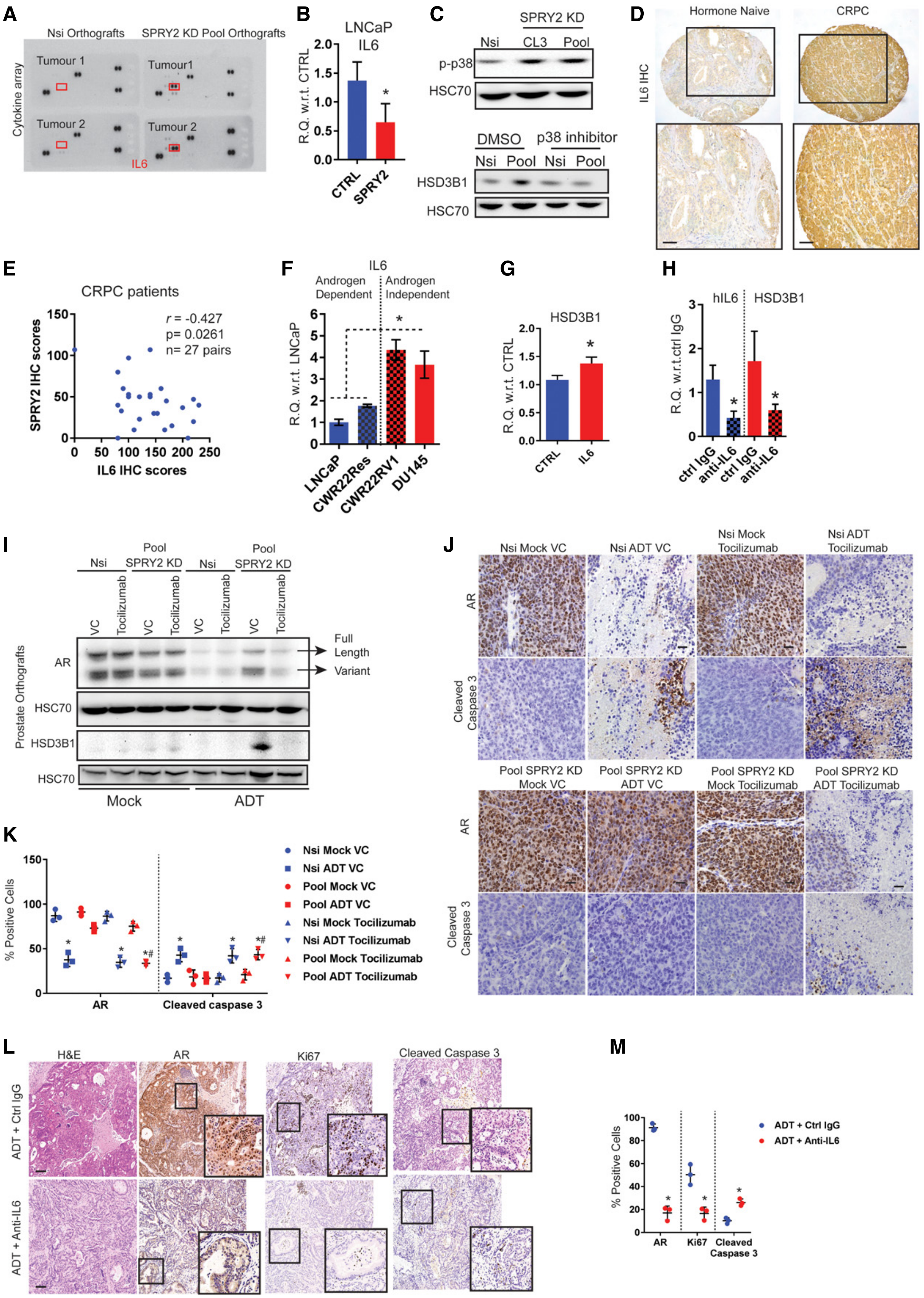
A
Nsi Orthografts
SPRY2 KD Pool Orthografts
Cytokine array
Tumour 1
Tumour1
Tumour 2
Tumour 2
IL6
B
LNCaP
IL6
R.Q. w.r.t. CTRL
CTRL
SPRY2
C
SPRY2 KD
Nsi
CL3
Pool
p-p38
HSC70
DMSO
p38 inhibitor
Nsi
Pool
Nsi
Pool
HSD3B1
HSC70
D
Hormone Naive
CRPC
IL6 IHC
E
CRPC patients
r = -0.427
p= 0.0261
n= 27 pairs
SPRY2 IHC scores
IL6 IHC scores
F
IL6
Androgen Dependent
Androgen Independent
R.Q. w.r.t. LNCaP
LNCaP
CWR22Res
CWR22RV1
DU145
G
HSD3B1
R.Q. w.r.t. CTRL
CTRL
IL6
H
hIL6
HSD3B1
R.Q. w.r.t.ctrl IgG
ctrl IgG
anti-IL6
ctrl IgG
anti-IL6
I
Nsi
Pool SPRY2 KD
Nsi
Pool SPRY2 KD
VC
Tocilizumab
Prostate Orthografts
AR
Full Length
Variant
HSC70
HSD3B1
HSC70
Mock
ADT
J
Nsi Mock VC
Nsi ADT VC
Nsi Mock Tocilizumab
Nsi ADT Tocilizumab
AR
Cleaved Caspase 3
Pool SPRY2 KD Mock VC
Pool SPRY2 KD ADT VC
Pool SPRY2 KD Mock Tocilizumab
Pool SPRY2 KD ADT Tocilizumab
K
% Positive Cells
AR
Cleaved caspase 3
Nsi Mock VC
Nsi ADT VC
Pool Mock VC
Pool ADT VC
Nsi Mock Tocilizumab
Nsi ADT Tocilizumab
Pool Mock Tocilizumab
Pool ADT Tocilizumab
L
H&E
AR
Ki67
Cleaved Caspase 3
ADT + Ctrl IgG
ADT + Anti-IL6
M
% Positive Cells
ADT + Ctrl IgG
ADT + Anti-IL6
AR
Ki67
Cleaved Caspase 3

Figure EV4.

Figure EV5. Targeting SRB1 in CRPC.

A, B Representative immunoblot image for indicated proteins in whole cell lysates from control and SRB1 knockout (KO) cells derived from (A) Nsi control and SPRY2-deficient (Pool) cells and (B) CWR22Res cells. GAPDH is used as the loading control.

C The cytotoxic effects of SRB1 antagonist ITX5061 relative to DMSO on CWR22Res control and SRB1 KO cells cultured for 48 h in RPMI with 10% charcoal-stripped serum (CSS) mimicking hormone-deprived (ADT) conditions. IC50 calculated using log (inhibitor) versus response – Variable slope (four parameters) with bottom constraint = 0.0 with GraphPad Prism software. IC50 of ITX5061 in CWR22Res control = 23.87 ± 0.677 and CWR22Res SRB1KO = not calculated due to lack of response (n = 3; IC50 data presented as mean ± SD).

D LDL uptake in indicated cells grown in medium with 10% FBS (FM) or 10% charcoal-stripped serum (ADT) treated with 15 μM ITX5061 (n = 3).

E Relative fluorescence in LNCaP (maintained in 10% FBS containing medium) and LNCaP AI (androgen-independent LNCaP cells maintained in 10% CSS-containing medium) treated with HDL or LDL bound to 1,1′-dioctadecyl-3,3,3′,3′-tetramethyllindocarbocyanine perchlorate (Dil) [Dil-HDL or Dil-LDL] (n = 3).

F Relative fluorescence in VCaP prostate cancer cells treated with HDL or LDL bound to 1,1′-dioctadecyl-3,3,3′,3′-tetramethyllindocarbocyanine perchlorate (Dil) [Dil-HDL or Dil-LDL] in medium containing 10% FBS or 10% CSS (n = 3; *P = 0.0073 compared to HDL treatment in FBS; unpaired two-tailed Student's t-test).

G–I Relative fluorescence in LNCaP (G), VCaP (H) and CWR22RV1 (I) cells treated with ITX5061 and HDL bound to 1,1′-dioctadecyl-3,3,3′,3′-tetramethyllindocarbocyanine perchlorate (Dil) [Dil-HDL] (n = 3; *$P < 0.05$ compared to HDL treatment in FBS; unpaired two-tailed Student's t-test).

J % Cholesterol efflux induced by human serum from Nsi control and SPRY2 KD (Pool) CWR22Res with SRB1 knockout (KO) as indicated in presence of 15 μM ITX5061 (n = 3; *$P < 0.05$ compared to Nsi control DMSO; $^{\#}P < 0.05$ compared to Nsi control ITX5061; ANOVA Tukey's test).

K Growth rate of control and SPRY2-expressing LNCaP cells relative to Day 0 (T0) in medium containing 10% CSS (hormone deficient) with indicated ITX5061 treatments (n = 3; *$P < 0.05$ compared to LNCaP CTRL; $^{\#}P < 0.05$ compared to LNCaP SPRY2; ANOVA Tukey's test).

L Growth rate of CWR22RV1 (CRPC) cells relative to T0 in medium containing 10% CSS (hormone deficient) with indicated ITX5061 treatments (n = 3; *$P < 0.05$ ANOVA Tukey's test).

M Representative images (n = 5) of CWR22Res prostate orthograft sections immunostained for AR and cleaved caspase 3 from mock- or ADT-treated mice. Scale bar = 10 μm.

N IHC quantification of indicated markers as presented in (M) (n = 3 mice analysed per group; *$P < 0.05$ compared to respective controls; unpaired two-tailed Student's t-test).

O Representative immunoblot images for indicated proteins in lysates from indicated CWR22Res orthografts.

Data Information: In (C, K, L), each data point represents mean with observation with error bars as SD. In (D–L), the data are presented as mean ± SD. In (N), data represented as mean.
Source data are available online for this figure.

 © 2018 The Authors

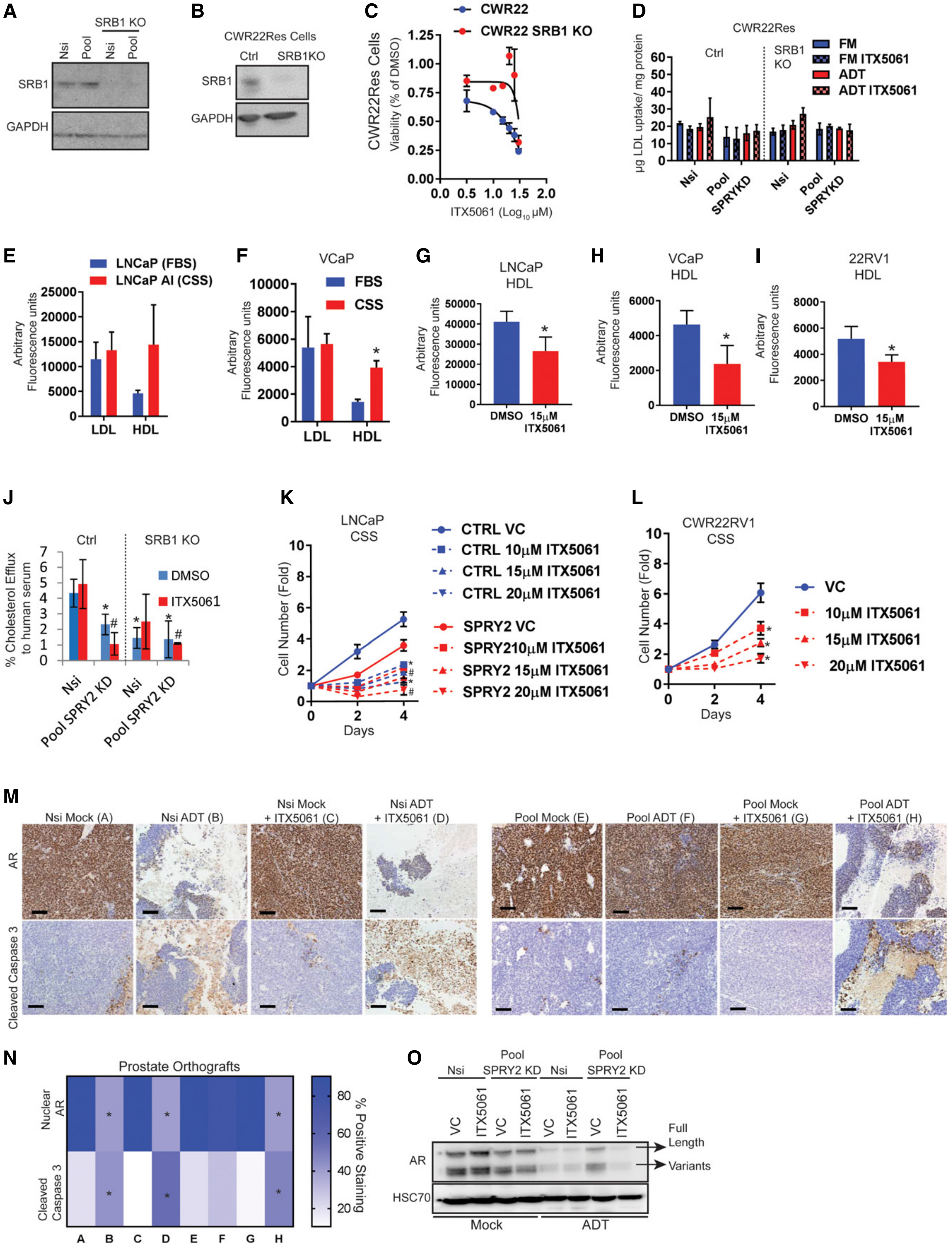
A
SRB1 KO
Nsi
Pool
Nsi
Pool
SRB1
GAPDH
B
CWR22Res Cells
Ctrl
SRB1KO
SRB1
GAPDH
C
CWR22
CWR22 SRB1 KO
CWR22Res Cells
Viability (% of DMSO)
ITX5061 (Log10 μM)
D
CWR22Res
Ctrl
SRB1 KO
FM
FM ITX5061
ADT
ADT ITX5061
μg LDL uptake/ mg protein
Nsi
Pool SPRYKD
E
LNCaP (FBS)
LNCaP AI (CSS)
Arbitrary Fluorescence units
LDL
HDL
F
VCaP
FBS
CSS
G
LNCaP HDL
DMSO
15μM ITX5061
H
VCaP HDL
I
22RV1 HDL
J
Ctrl
SRB1 KO
DMSO
ITX5061
% Cholesterol Efflux to human serum
Nsi
Pool SPRY2 KD
K
LNCaP CSS
CTRL VC
CTRL 10μM ITX5061
CTRL 15μM ITX5061
CTRL 20μM ITX5061
SPRY2 VC
SPRY210μM ITX5061
SPRY2 15μM ITX5061
SPRY2 20μM ITX5061
Cell Number (Fold)
Days
L
CWR22RV1 CSS
VC
10μM ITX5061
15μM ITX5061
20μM ITX5061
M
Nsi Mock (A)
Nsi ADT (B)
Nsi Mock + ITX5061 (C)
Nsi ADT + ITX5061 (D)
Pool Mock (E)
Pool ADT (F)
Pool Mock + ITX5061 (G)
Pool ADT + ITX5061 (H)
AR
Cleaved Caspase 3
N
Prostate Orthografts
Nuclear AR
Cleaved Caspase 3
% Positive Staining
A B C D E F G H
O
Nsi
Pool SPRY2 KD
VC
ITX5061
AR
HSC70
Full Length
Variants
Mock
ADT

Figure EV5.
