# Supplementary figures and images for: Sprouty2 loss‐induced IL6 drives castration‐resistant prostate cancer through scavenger receptor B1
Source: EMBO Mol Med. 2018 Mar 14;10(4):e8347. doi: 10.15252/emmm.201708347 (PMC5887544; doi:10.15252/emmm.201708347)

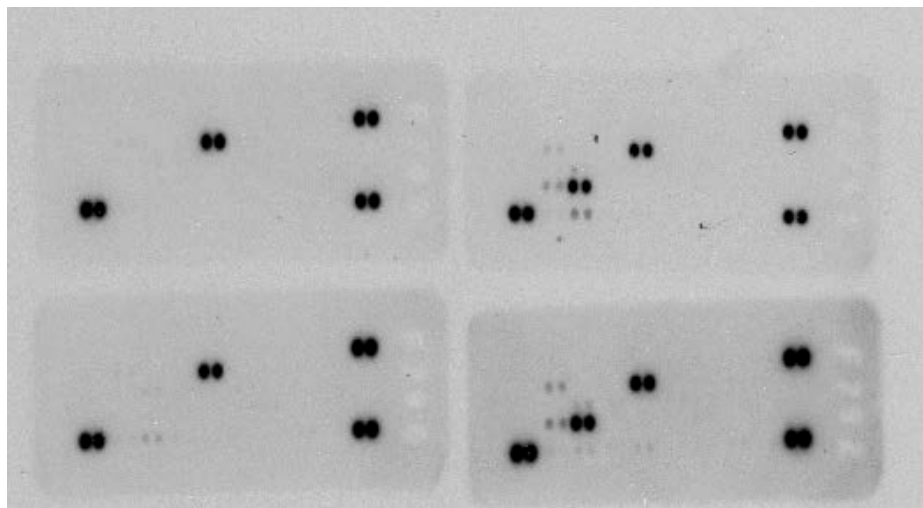

EV4A

~38 —

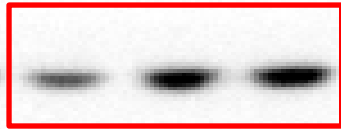

P-p38

~70 —

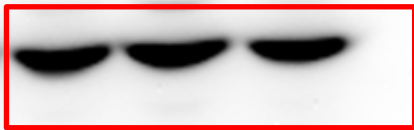

HSC70

EV4C top panel

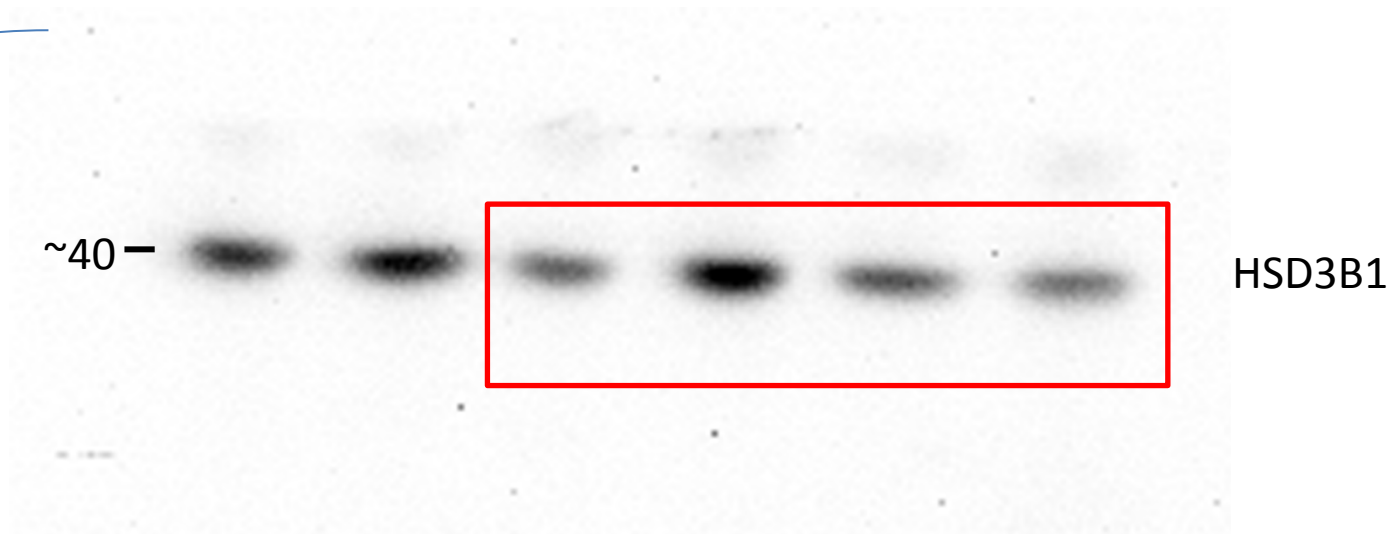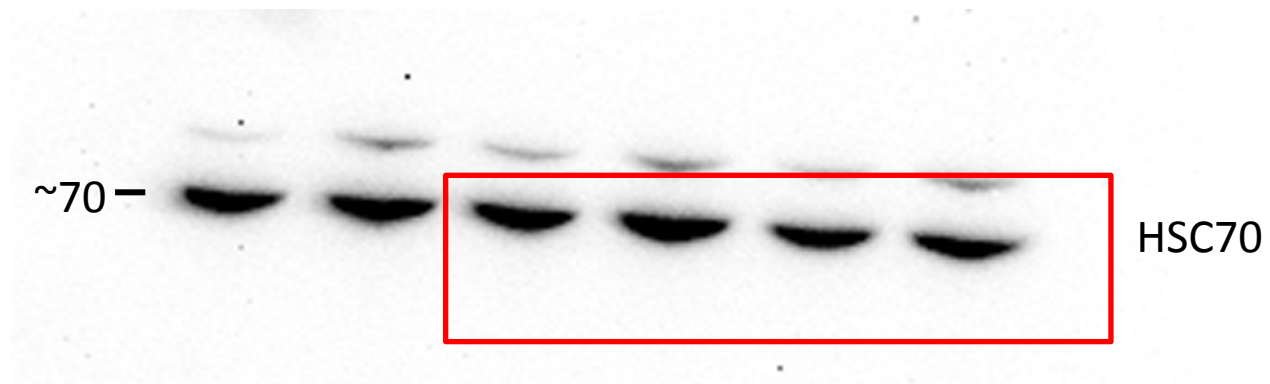

EV4C bottom panel

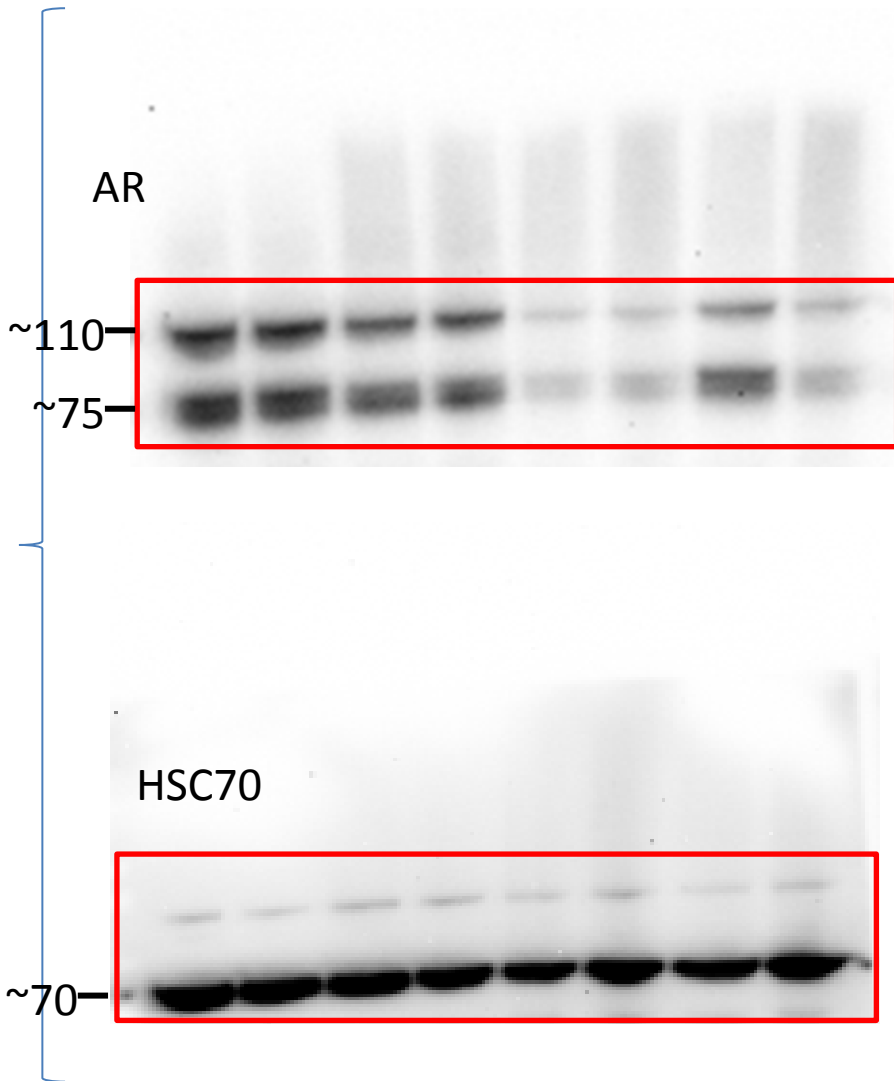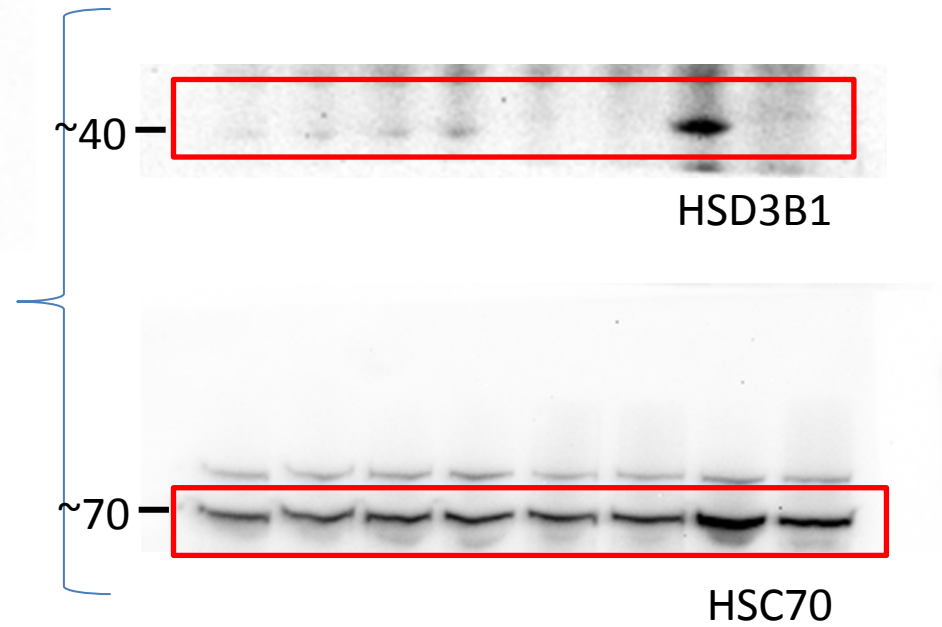

EV4I

Supplement: Supplementary file 3 — Source Data for Expanded View and Appendix [file EMMM-10-e8347-s005.zip › EV4.pdf]

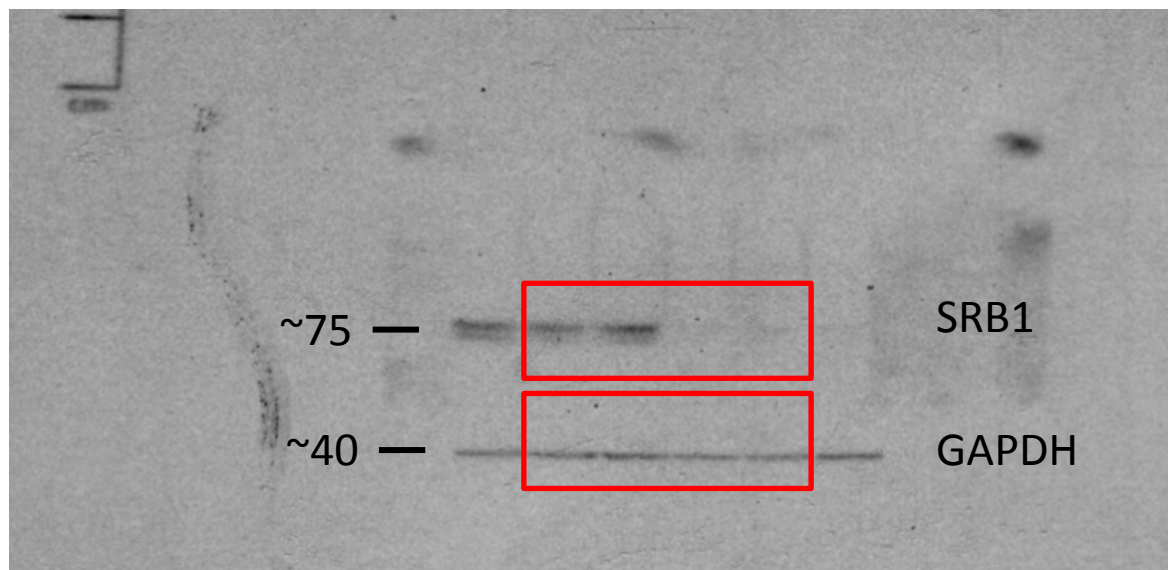

Fig EV5A

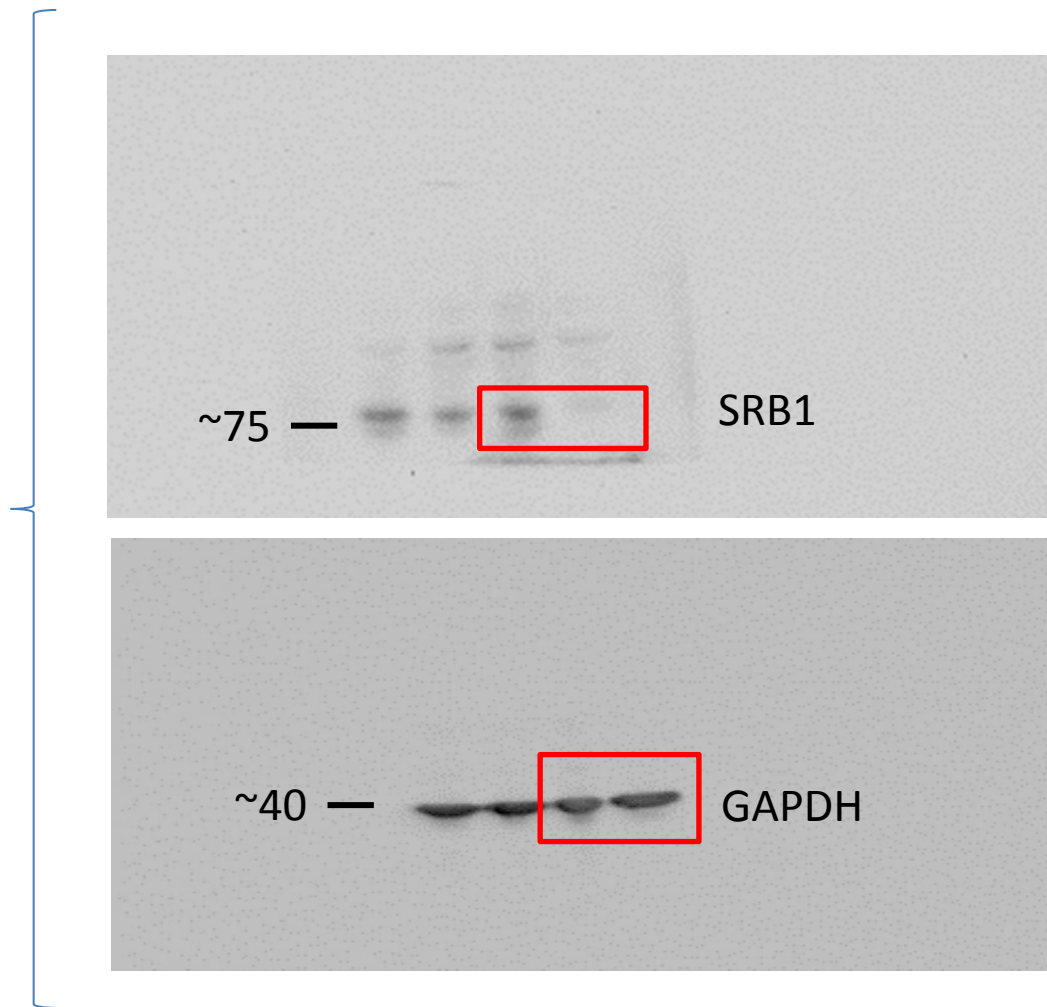

Fig EV5B

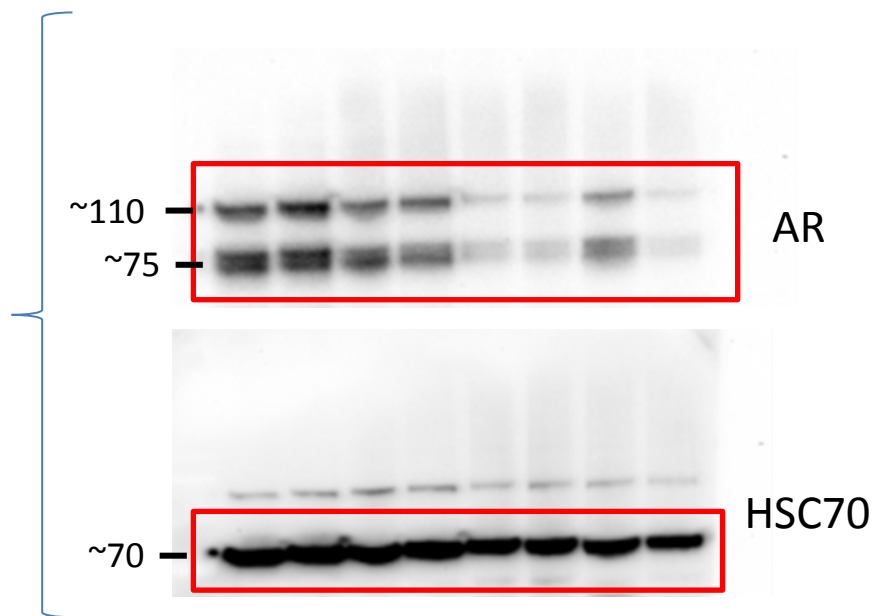

Fig EV50

Supplement: Supplementary file 3 — Source Data for Expanded View and Appendix [file EMMM-10-e8347-s005.zip › EV5.pdf]

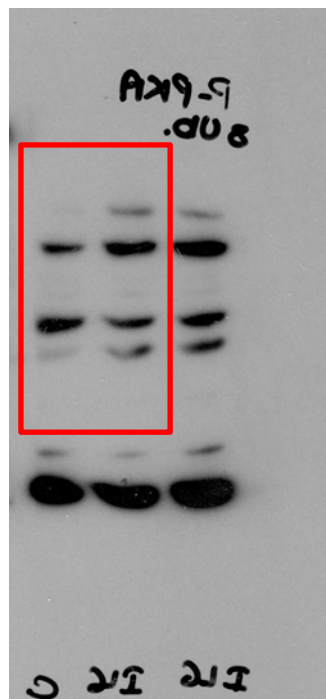

P-PKA substrate

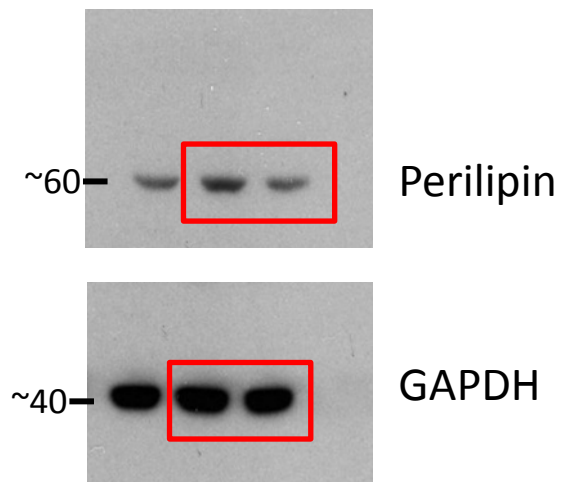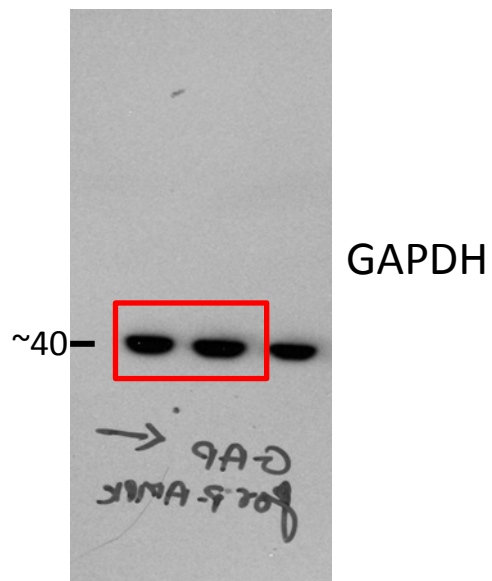

Appendix Figure S2H

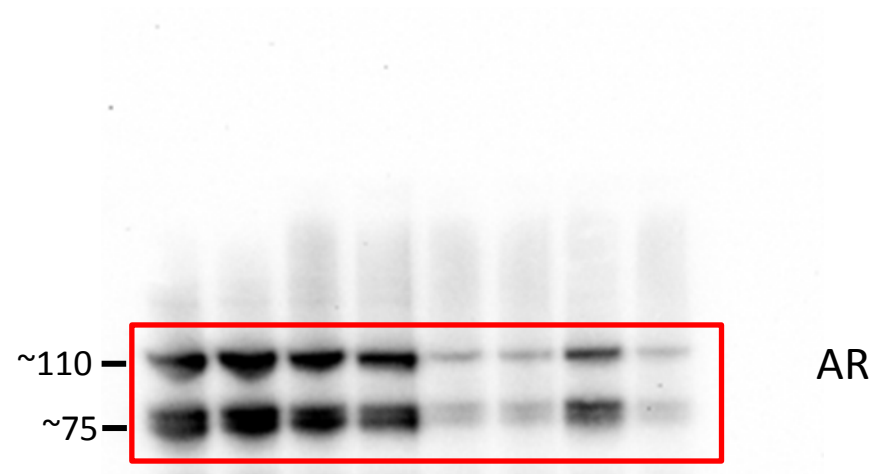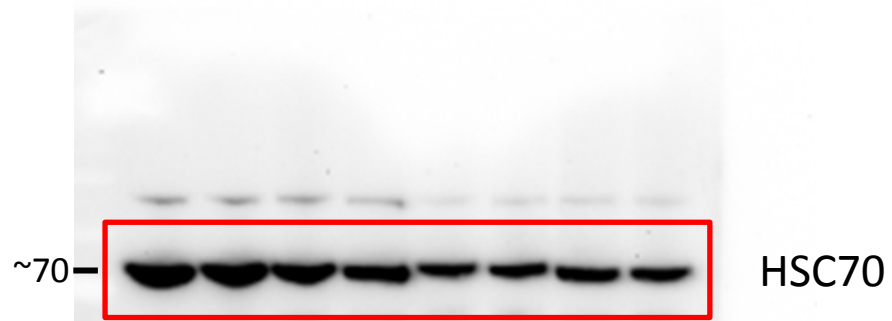

Appendix Figure S20

Supplement: Supplementary file 3 — Source Data for Expanded View and Appendix [file EMMM-10-e8347-s005.zip › Appendix_Figure_2.pdf]

~110 —

~75 —

AR

~70 —

HSC70

~35 —

SPRY2

~70 —

HSC70

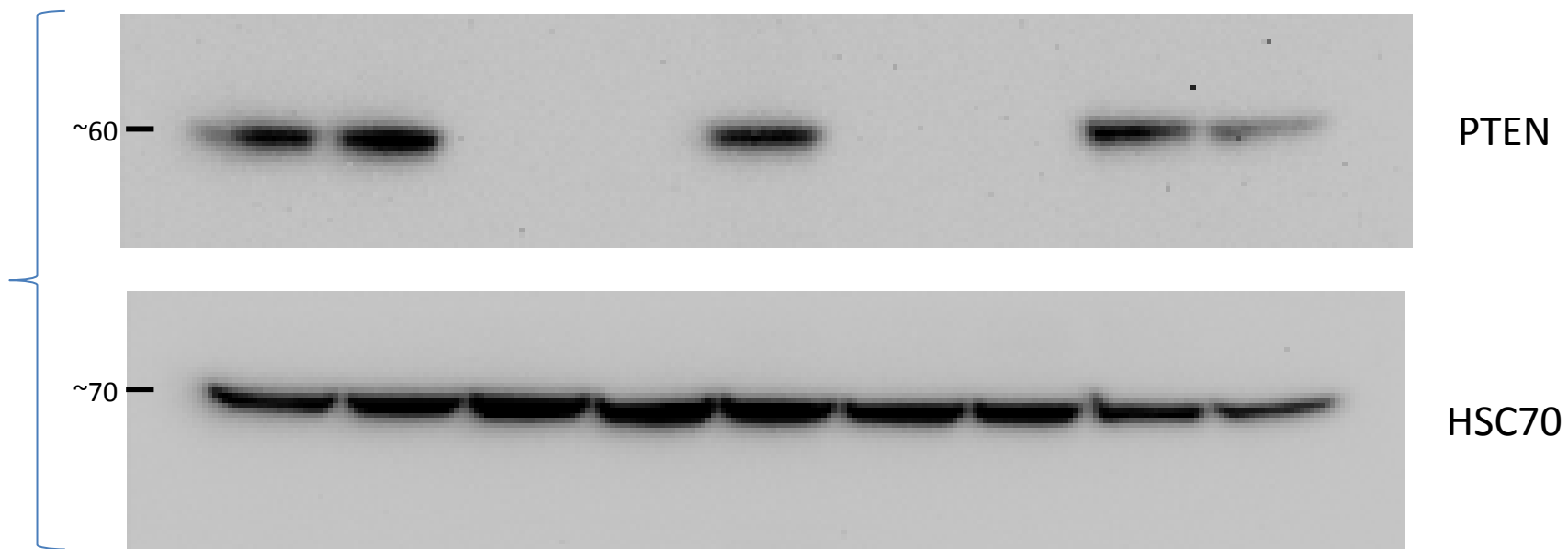

Supplement: Supplementary file 3 — Source Data for Expanded View and Appendix [file EMMM-10-e8347-s005.zip › EV1.pdf]

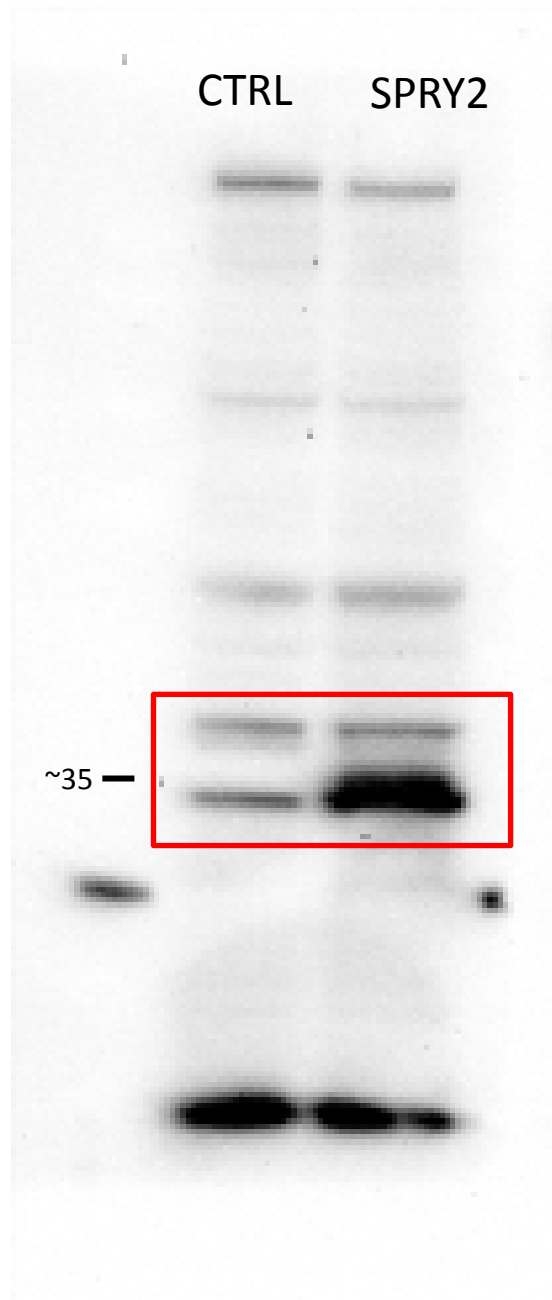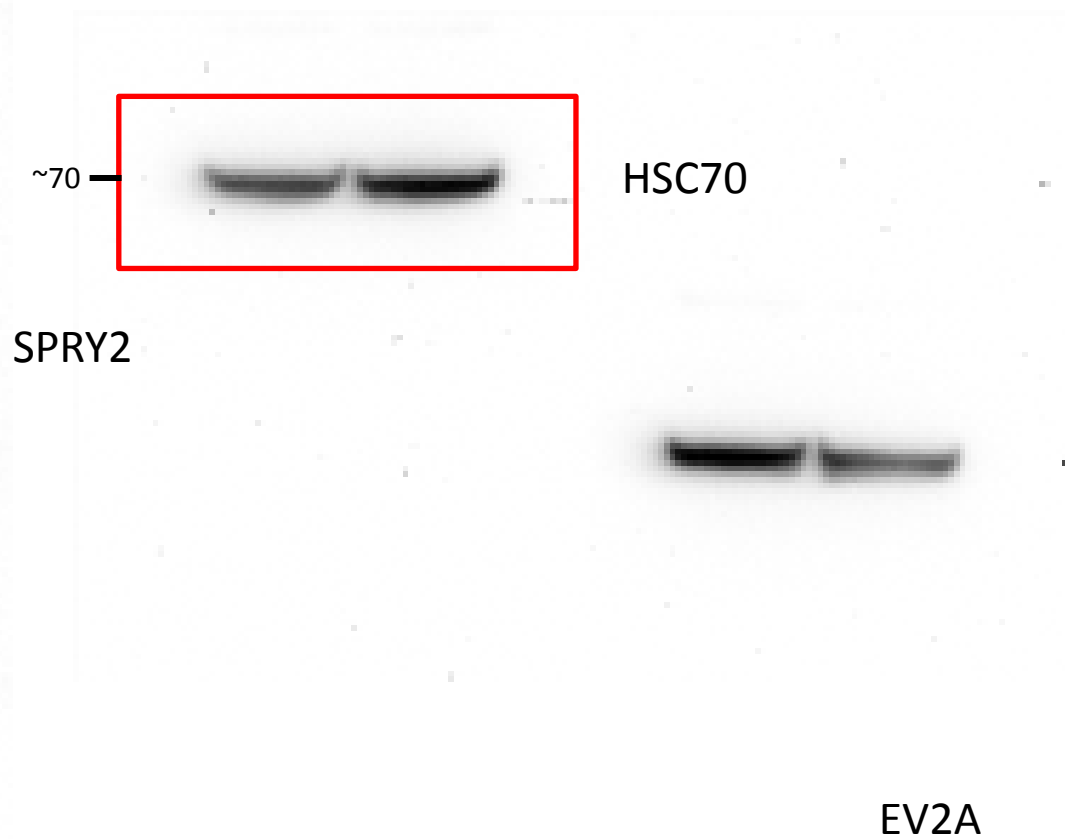

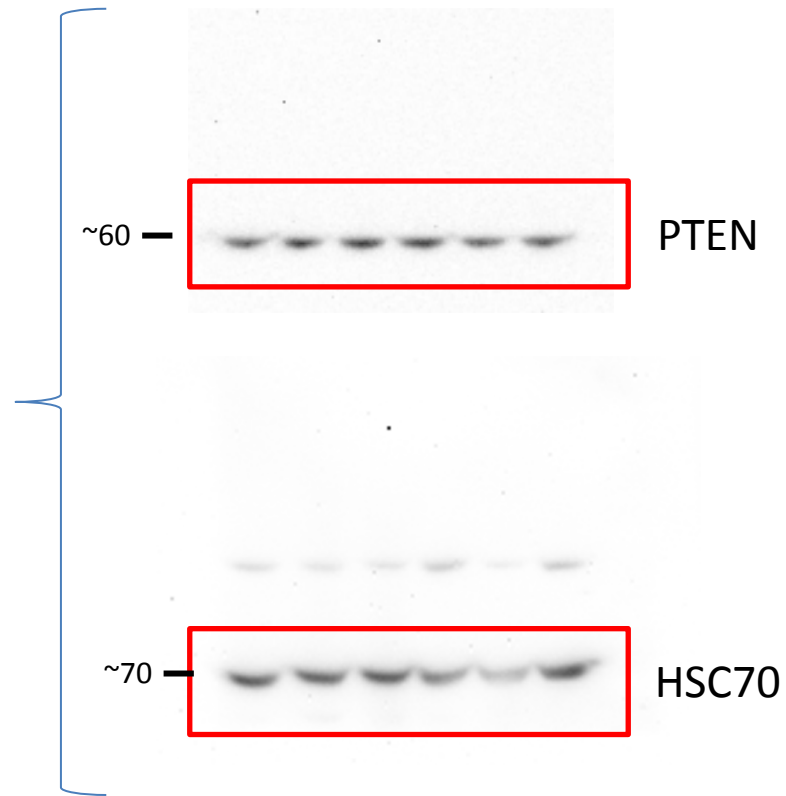

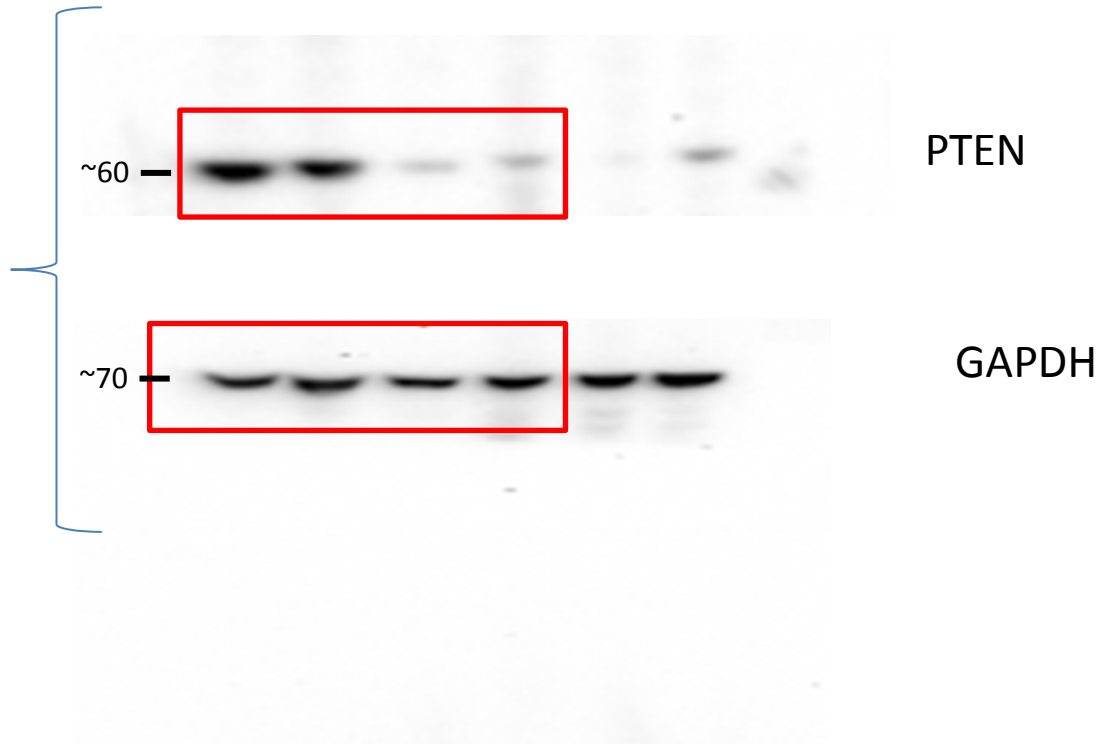

EV2N

Supplement: Supplementary file 3 — Source Data for Expanded View and Appendix [file EMMM-10-e8347-s005.zip › EV2.pdf]

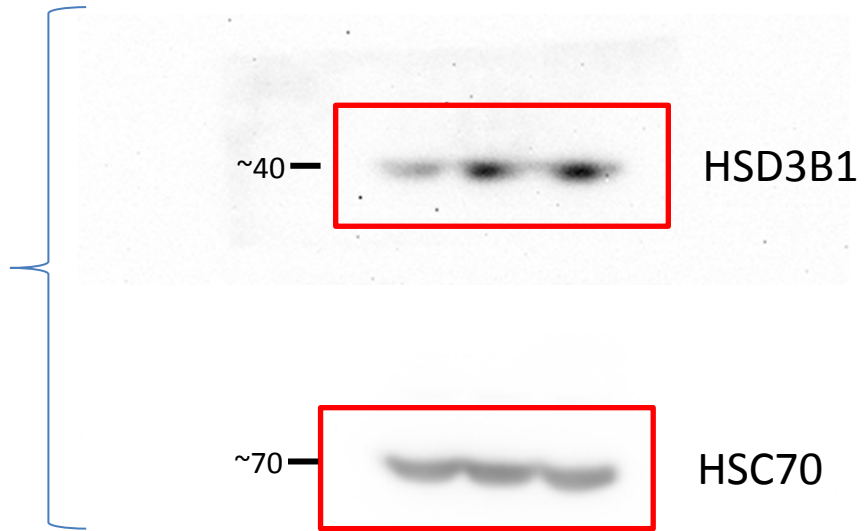

EV3D

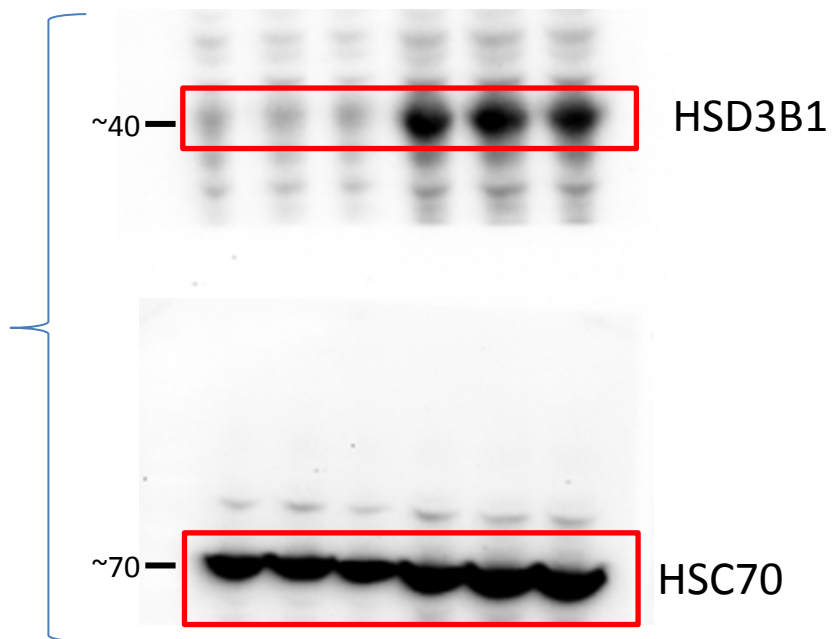

EV3F

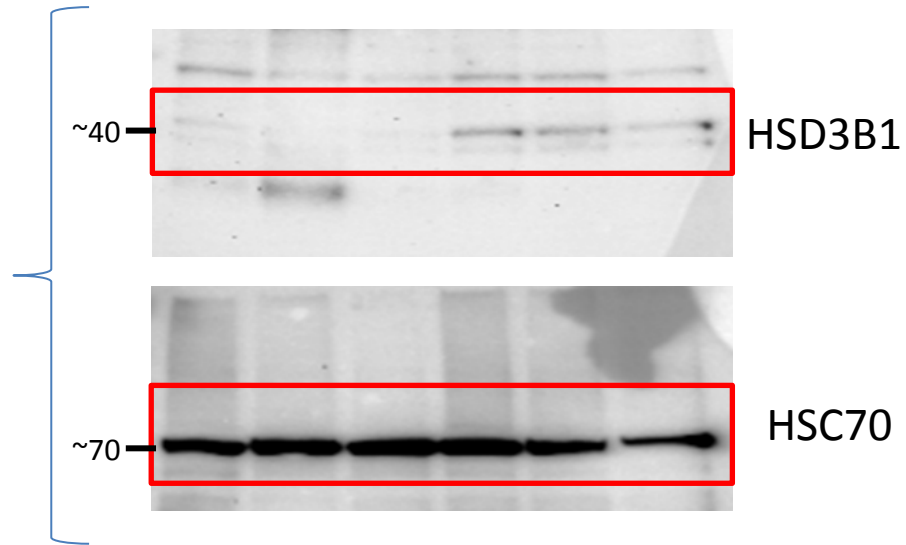

EV3H

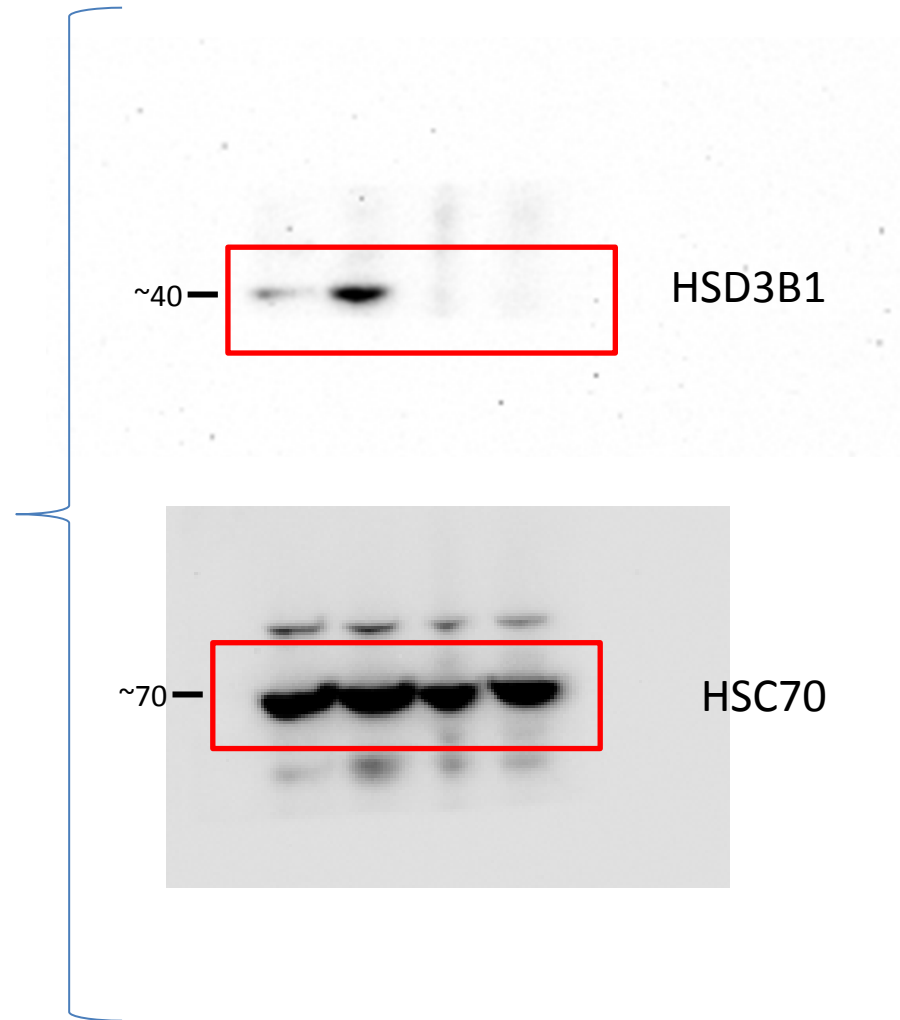

EV3K

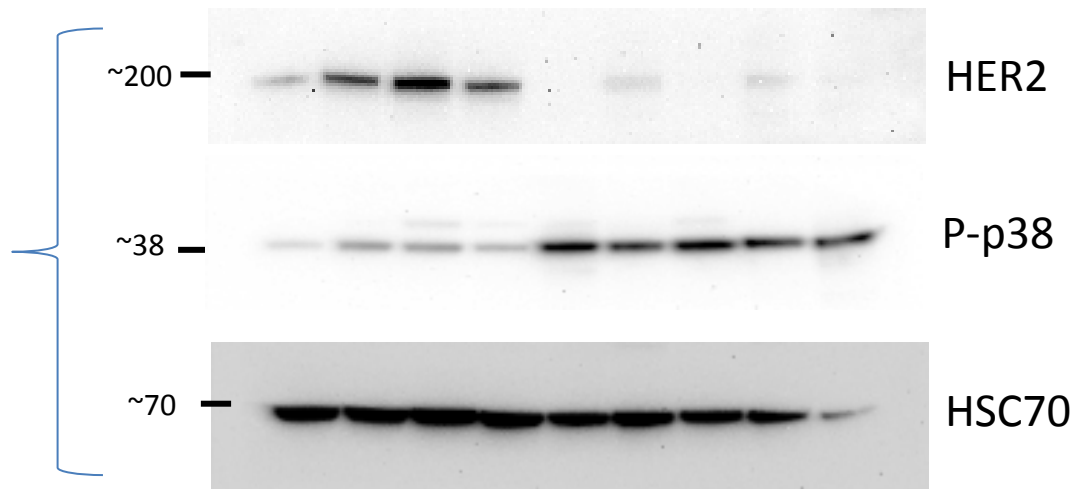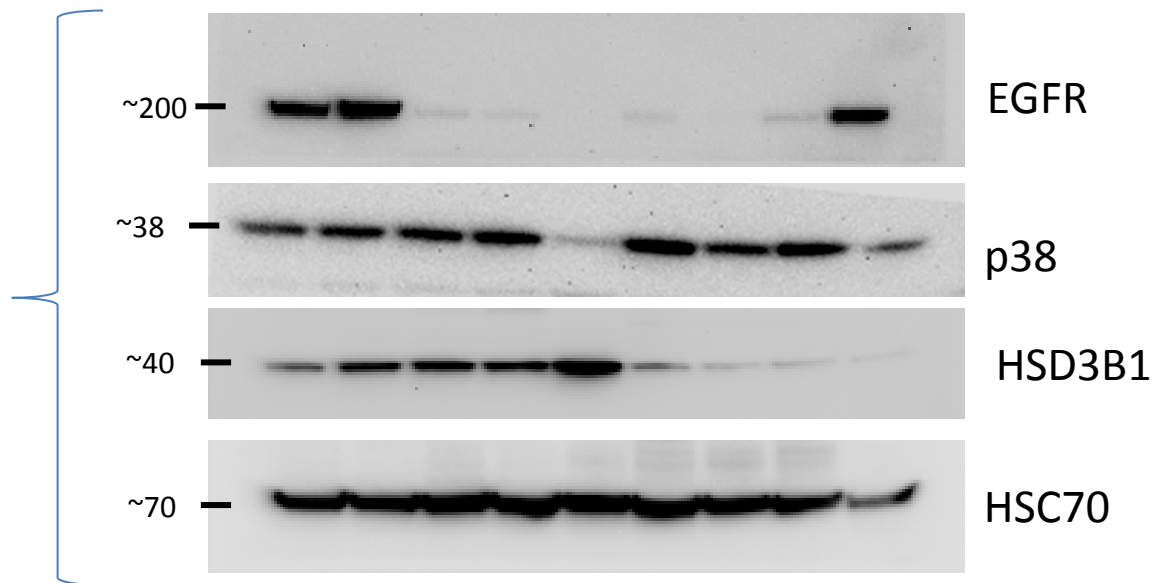

EV3N

Supplement: Supplementary file 3 — Source Data for Expanded View and Appendix [file EMMM-10-e8347-s005.zip › EV3.pdf]

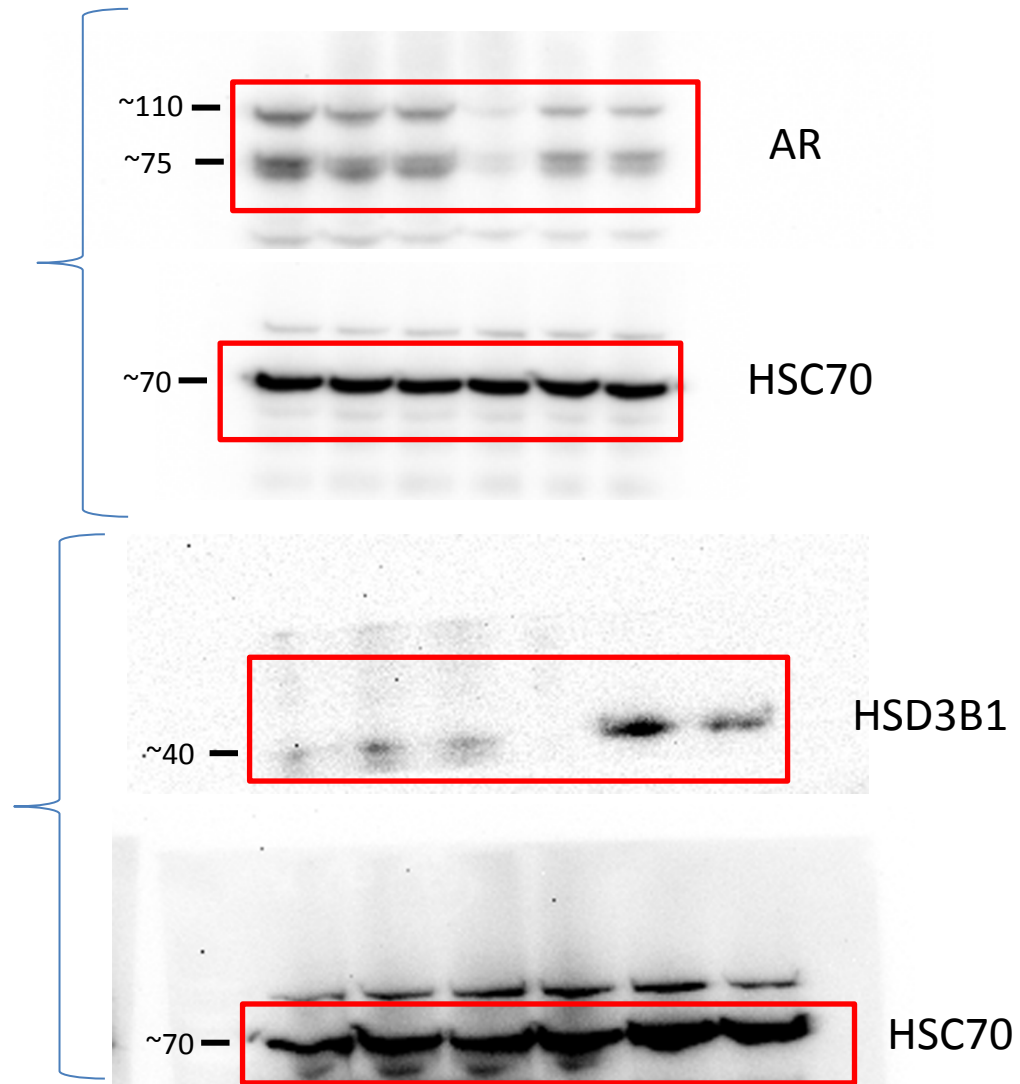

Fig 2A

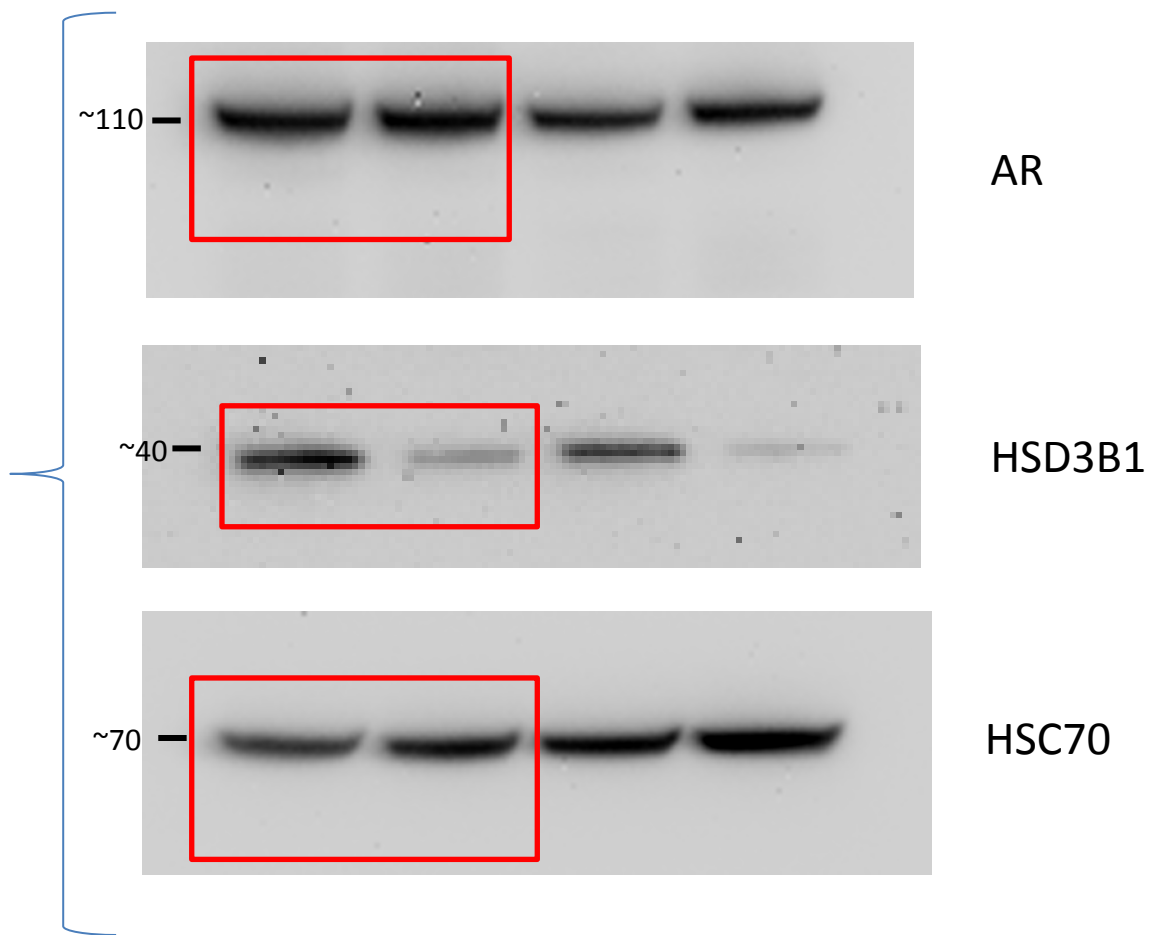

Fig 2I

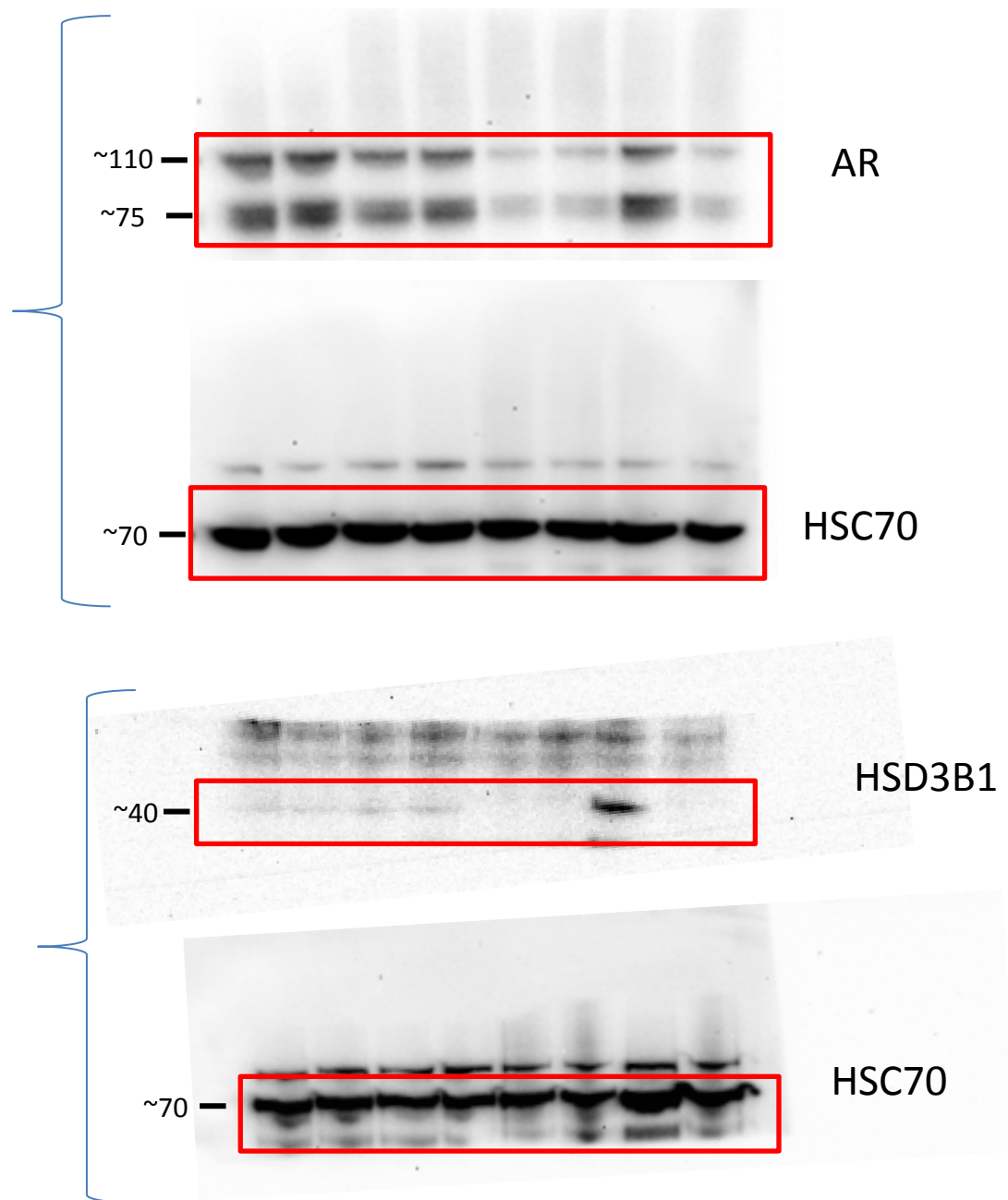

Fig 2N

Supplement: Supplementary file 5 — Source Data for Figure 2 [file EMMM-10-e8347-s003.pdf]

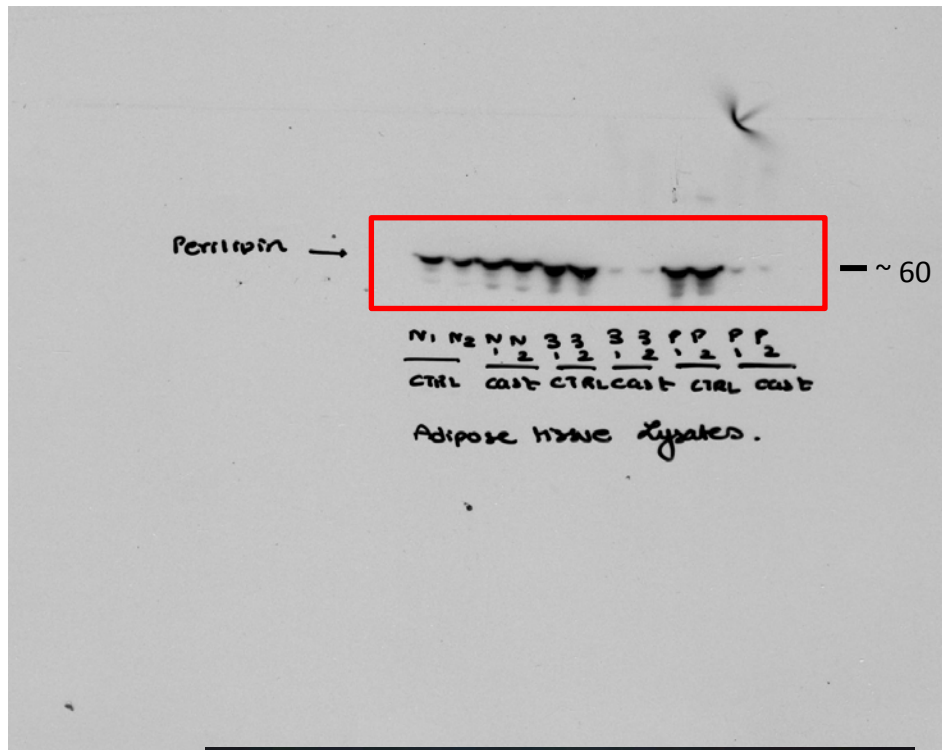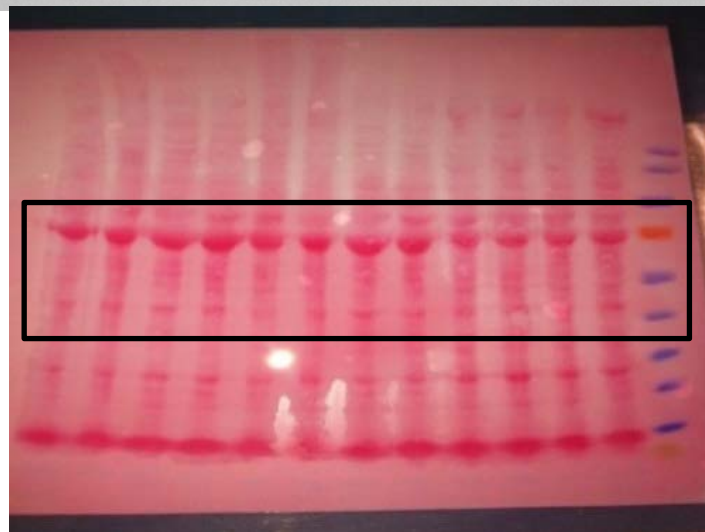

Fig 4K

Supplement: Supplementary file 6 — Source Data for Figure 4 [file EMMM-10-e8347-s004.pdf]
